# Multiple illumination learned spectral decoloring for quantitative optoacoustic oximetry imaging

**DOI:** 10.1117/1.JBO.26.8.085001

**Published:** 2021-08-04

**Authors:** Thomas Kirchner, Martin Frenz

**Affiliations:** University of Bern, Biomedical Photonics, Institute of Applied Physics, Bern, Switzerland

**Keywords:** quantitative photoacoustic imaging, photoacoustics, multiple illumination sensing, machine learning, blood oxygen saturation

## Abstract

**Significance:** Quantitative measurement of blood oxygen saturation (${\mathrm{sO}}_{2}$) with optoacoustic (OA) imaging is one of the most sought after goals of quantitative OA imaging research due to its wide range of biomedical applications.

**Aim:** A method for accurate and applicable real-time quantification of local ${\mathrm{sO}}_{2}$ with OA imaging.

**Approach:** We combine multiple illumination (MI) sensing with learned spectral decoloring (LSD). We train LSD feedforward neural networks and random forests on Monte Carlo simulations of spectrally colored absorbed energy spectra, to apply the trained models to real OA measurements. We validate our combined MI-LSD method on a highly reliable, reproducible, and easily scalable phantom model, based on copper and nickel sulfate solutions.

**Results:** With this sulfate model, we see a consistently high estimation accuracy using MI-LSD, with median absolute estimation errors of 2.5 to 4.5 percentage points. We further find fewer outliers in MI-LSD estimates compared with LSD. Random forest regressors outperform previously reported neural network approaches.

**Conclusions:** Random forest-based MI-LSD is a promising method for accurate quantitative OA oximetry imaging.

## 1 Introduction

A robust and accurate quantitative measurement of blood oxygen saturation (${\mathrm{sO}}_{2}$) with optoacoustic (OA) imaging, also called photoacoustic imaging, is one of the most sought after goals of quantitative OA imaging research due to its wide range of immediate applications. Usually, quantitative OA imaging research aims to achieve an absolute quantification of optical properties, such as the absorption coefficient $\mu_{a}$, from measured OA signals S(d,t) recorded at times t at detector position d.^1^^,^^2^ In brief, such a quantification of $\mu_{a}$ encompasses a solution of two ill-posed inverse problems. (1) The acoustic inverse problem from S(d,t) to an initial pressure spatial distribution $p_{0}(x)$. And (2), the optical inverse problem from $H(x_{0})=p_{0}(x_{0})/\Gamma (x_{0})=\phi (x_{0},\mu_{a}(x),\mu_{s}^{\prime }(x))\cdot \mu_{a}(x_{0})$ to $\mu_{a}(x_{0})$, at a location $x_{0}$, with the Grüneisen parameter Γ and the reduced scattering coefficient $\mu_{s}^{\prime }$. The fluence ϕ is dependent on unknowns such as the absorption and scattering in the tissue surrounding $x_{0}$. Quantitative OA imaging methods either depend on model-based inversion^2^^–^^7^ or data-driven approaches.^8^^–^^13^ These approaches perform well *in silico* but often struggle with the translation to real measurements in phantoms or *in vivo*.

In OA imaging, ${\mathrm{sO}}_{2}$ estimations are derived from multispectral OA measurements by first performing an acoustic reconstruction yielding images of the OA signal

$$S(x_{0},\lambda )=\Gamma (x_{0})\cdot A(x_{0})\cdot \phi (x_{0},\mu_{a}(x,\lambda ),\mu_{s}^{\prime }(x,\lambda ))\cdot \mu_{a}(x_{0},\lambda ),$$ (1)

for each measured wavelength λ, with $A(x_{0})$ being an unknown spatially varying factor introduced by the imperfectly solved acoustic ill-posed inverse problem (i.e., image reconstruction from data with limited frequency bandwidth and a limited probe aperture). Using a linear image reconstruction, the acoustic inverse problem can be assumed as wavelength independent. The spectral coloring^1^ due to the wavelength-dependent fluence variation causes the dominant distortion in any ${\mathrm{sO}}_{2}$ estimation made from multispectral signal stacks S(x,λ). This spectral coloring of OA signals needs to be corrected to yield accurate quantitative estimates of ${\mathrm{sO}}_{2}$. To address this need, we combine two approaches to quantitative OA imaging of ${\mathrm{sO}}_{2}$. (1) Multiple illumination (MI) sensing^14^—a method in which a sequence of OA measurements is acquired with a sequence of illuminations at different positions. Usually, effective attenuation of the illumination is then estimated with diffusion theory and then used for correcting spectral coloring. (2) Learned spectral decoloring (LSD)^15^—a data science method in which a machine learning algorithm is trained on Monte Carlo simulations of spectrally colored multispectral OA measurements to decolor real measurements.

Both these methods can yield promising results on their own but still suffer from a range of constraints, i.e., MI sensing implementations^16^ typically assume and use point illuminations, which enables the use of closed-form solutions of the diffusion approximation of light propagation^14^ but limits SNR due to the laser safety limit for skin.^17^ The resulting long acquisition times make this method difficult to translate to realistic macroscopic applications.^18^ Furthermore, MI sensing so far has theoretical limits in highly inhomogeneous scenes due to its reliance on the diffusion approximation. MI sensing implementations usually aim to estimate absolute values of $\mu_{a}$, which goes beyond what is needed for an estimation of ${\mathrm{sO}}_{2}$. LSD^15^^,^^19^ and similar spectral approaches^3^ currently yield accurate *in silico* estimations and plausible initial results in highly constrained settings, but they have insufficient input to robustly generalize these results over diverse geometries and applications.

We will investigate LSD as a method to analyze MI data. Both MI sensing and LSD are not yet thoroughly validated; partially due to a lack of stable and reliable ${\mathrm{sO}}_{2}$ phantoms. Even though substantial progress has been made in dynamic blood flow phantoms for OA imaging validation, these blood or red blood cell suspension phantoms require extensive fine tuning and even then yield reference values with limited accuracy.^20^ At best, a reference measurement of 2% to 4% is achievable with state-of-the-art blood flow phantoms.^21^^,^^22^ While validating quantitative OA oximetry methods, the validation phantoms are also often restricted to the extreme ${\mathrm{sO}}_{2}$ values of 0% and 100% because other values cannot be set reliably.^23^ This causes an incomplete range and therefore insufficient validation.

Rather than implement such an ${\mathrm{sO}}_{2}$ flow phantom, we used copper and nickel sulfate solutions in a relative copper sulfate model similar to work by Buchmann et al.^24^ to mimic absorption spectra of blood with different oxygen saturation. This allowed a reliable sub 1% error in our ground truth and allowed us to rapidly manufacture stable and highly reproducible phantoms with wide variations in optical properties to generate high quality test sets for spectral decoloring methods.

## 2 Materials and Methods

We investigated a method combining LSD and MI measurements. To that effect we

1. developed a system to perform real-time MI-multispectral OA imaging,
2. implemented modified LSD machine learning algorithms using MI,
3. used these algorithms to train on exclusively *in silico* data from Monte Carlo optical forward simulations with a relative copper sulfate model, and
4. validated and tested these machine learning models on comprehensive phantom measurements using the copper and nickel sulfate-based ${\mathrm{sO}}_{2}$ model.

### 2.1 Multiple Illumination Optoacoustic Imaging

**Fig. 1:**
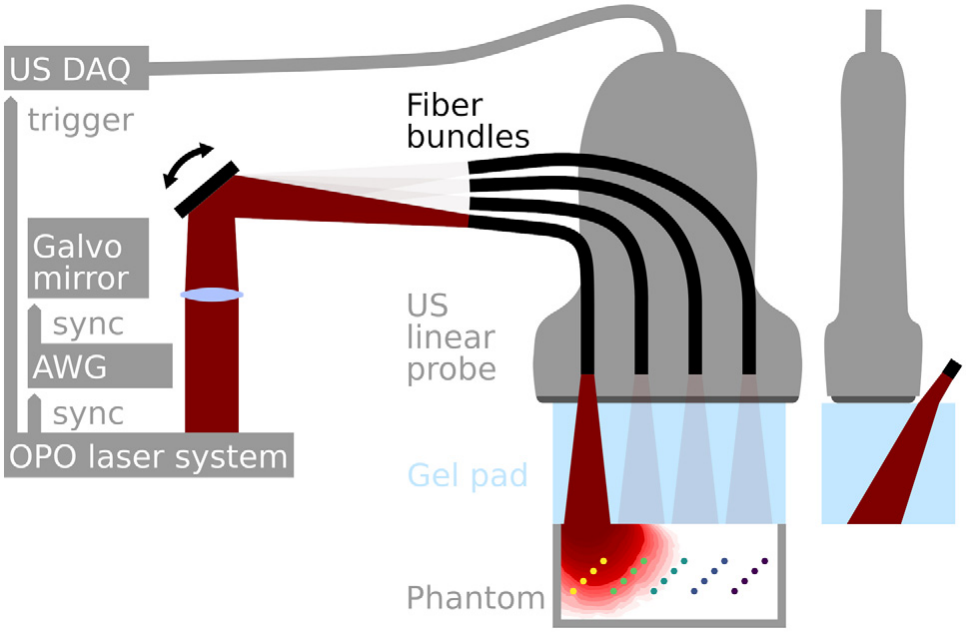
MI OA imaging setup. Illumination via fast tunable OPO laser sequentially illuminating fiber bundles using a galvo mirror system driven by an AWG. OA signals were measured with a linear array ultrasound (US) probe and recorded by a 64-channel US data acquisition (DAQ) system. An US gel pad is used to allow for in plane illumination.

Our MI OA imaging setup is shown in Fig. 1. It uses a fast wavelength-tunable optical parametric oscillator (OPO) laser system (prototype SpitLight, InnoLas Laser GmbH, Krailling, Germany) with 5-ns pulse duration and 100-Hz pulse repetition frequency. The laser pulses were sequentially coupled into four high power fiber bundles (FiberOptic P.+P. AG, Spreitenbach, Switzerland) with NA 0.22 fibers, each bundle with a 2-mm diameter. This was achieved using a galvo mirror system (GVS011/M, Thorlabs Inc., Newton, New Jersey, USA) driven by an arbitrary waveform generator (AWG) (TG5011, Aim-TTi, Cambridgeshire, UK), which was synchronized with the laser system. The fiber bundle output sides were arranged in a line array with 8 mm spacing. The illumination pulses were attenuated to have a maximum energy of 10 mJ per pulse at the fiber output. To comply with ANSI safety limits,^17^^,^^25^ the beams are widened to 7-mm full-width at half-maximum (FWHM) at the tissue or phantom surface. Illumination and acoustic detection ensue through 18-mm-thick ultrasound gel pad (Parker Laboratories Inc., Fairfield, New Jersey, USA). We measure the 64 center channels of a 128-element linear array transducer (L7-4, Advanced Technology Laboratories Inc., Bothell, Washington, USA) with a center frequency of 5 MHz, a pitch of 0.3 mm, and a fractional bandwidth of 80%. The number of acquisition channels was limited by our 64 channel US data acquisition system (V-1-64, Verasonics, Inc., Kirkland, Washington, USA). For this study, we used the full tuning range of our OPO and acquired OA measurements for 16 equidistant wavelengths from 680 to 980 nm in 20 nm steps, each for four illumination positions. After firing one pulse of one wavelength in each fiber bundle, the wavelength is tuned to the next in sequence. Using this 4×16 sequence, each MI and multispectral stack of OA images takes 640 ms to acquire. We generally recorded the raw data for 30 such stacks for each scan. Live beamforming and visualization with 25 fps was performed using custom MATLAB scripts but this live visualization was solely used for probe positioning and quality control (e.g., avoiding air inclusions under the gel pad).

### 2.2 Image Processing

The acoustic reconstruction of OA images for further analysis was performed using the OA image processing module from the Medical Imaging Interaction Toolkit (MITK).^26^ The raw data were beamformed using a delay and sum (DAS) algorithm, with a fixed speed of sound of $1480\;{\mathrm{ms}}^{-1}$ and a Hann apodization over an angle of ±30  deg. For noise reduction, the beamformed data were bandpassed. A B-mode image was formed using an envelope detection filter and downsampling the result to a 0.15-mm isometric resolution. The full image processing pipeline including all relevant parameters is part of the open source appendix (see the Code, Data, and Materials Availability section). The B-mode images were corrected for the mean laser pulse energy at a specific wavelength. This mean laser pulse energy correction was determined directly at the fiber bundles output before the experiments—averaging the pulse energy for 30 laser pulses of each wavelength. For a single wavelength, the variation of pulse energy was <3%; to reduce this noise component’s influence, we also averaged our OA measurements over 30 full stacks of measurements.

### 2.3 Phantoms

The phantoms used consisted of arrays of polythene tubing (Smiths Medical International Ltd., Kent, UK) with 0.58-mm inner diameter and 0.96-mm outer diameter. These tubes were filled with a relative copper sulfate model solution (as detailed in Sec. 2.3.1) and arranged as shown in Sec. 2.3.3. The relative copper (rCu) in this model is mimicking blood oxygenation (${\mathrm{sO}}_{2}$).

Selecting the same small size tubes allowed us to rapidly assemble and modify phantoms with many target structure locations. The small size of the tubes was also chosen because the rCu solution in the tube did not include a scattering agent.

For all the phantom experiments, the background scattering medium was a fat emulsion (SMOFlipid 20%, Fresenius Kabi, Switzerland) diluted to 1.5% fat content. To avoid errors introduced by interbatch variations in the scattering properties of stock fat emulsions, such as intralipid or SMOFlipid, the optical properties of the used stock emulsion were assessed with a time-correlated single photon counting (TCSPC) technique as detailed in Sec. 2.3.2.

#### 2.3.1 Relative copper sulfate model

**Fig. 2:**
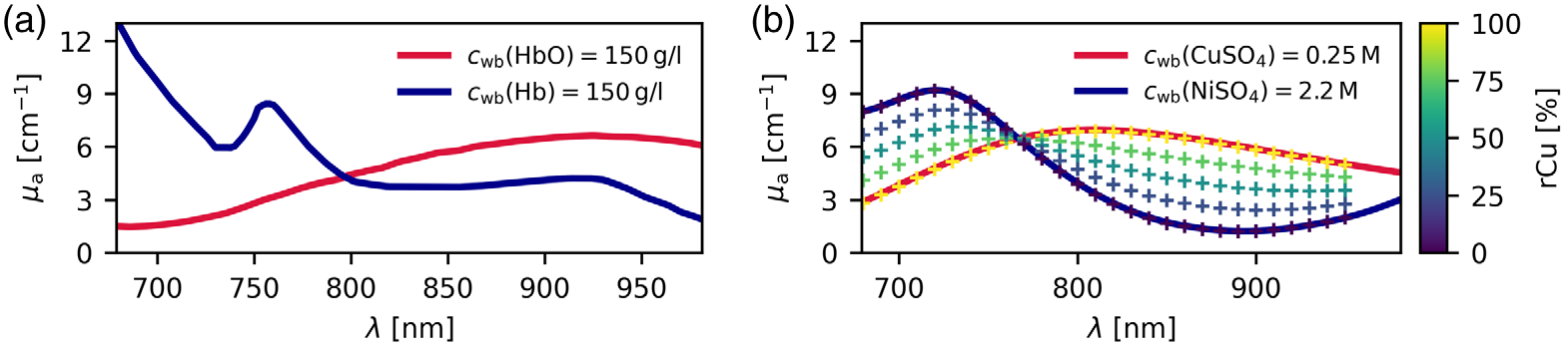
Absorption coefficient $\mu_{a}$ spectra. (a) Oxy- and deoxyhemoglobin at whole blood concentrations $c_{\mathrm{wb}}(\mathrm{HbT})=150\;{\mathrm{gl}}^{-1}$. (b) Copper and nickel sulfate in aqueous solution in whole blood equivalent solutions using a relative copper sulfate (rCu) model. The reference measurements of the five rCu mixtures used in our phantoms are plotted as “+.” Sulfate spectra were measured with a spectrophotometer.

The relative copper sulfate model solution was based on a 2.2-molar nickel sulfate (${\mathrm{NiSO}}_{4}$) water solution, produced using nickel(II) sulfate hexahydrate (>98%, Sigma-Aldrich), and on a 0.25 molar copper sulfate (${\mathrm{CuSO}}_{4}$) water solution, produced using copper(II) sulfate pentahydrate (>98%, Sigma-Aldrich).^27^ As shown in Fig. 2, these chromophores are mimicking the NIR absorption spectra of oxy- and deoxyhemoglobin in average whole blood with a hemoglobin concentration $c_{\mathrm{wb}}(\mathrm{HbT})=150\;{\mathrm{gl}}^{-1}$.^28^ Copper and nickel sulfate were also chosen for their temporal stability and resistance to bleaching.

The spectra of the sulfate solutions absorption coefficients $\mu_{a}$ in whole blood mimicking concentrations are defined as $c_{\mathrm{wb}}$ (${\mathrm{NiSO}}_{4}$) :=2.2 M and $c_{\mathrm{wb}}$ (${\mathrm{CuSO}}_{4}$) :=0.25 M. These solutions were measured using a 2-mm quartz cuvette (QS Hellma, Müllheim, Germany) in a UV–VIS–NIR spectrophotometer (Perkin Elmer Lambda 750, Waltham, Massachusetts, USA), in the range of 680 to 980 nm. The scattering in this wavelength range is negligible.^27^ The initial reference measurements were done in 2 nm steps, with a 10-s integration time and using a photomultiplier tube sensor. The absorption spectroscopy measurements were repeated on the solutions after 70 days to verify their stability over time. Whenever new batches of the sulfate solutions were produced, their absorption spectra were checked against the spectra of the first batch. The solutions were corrected when they deviated from the reference spectra by more than 1%.

The relative copper (rCu) in this model aims to mimic blood oxygenation (${\mathrm{sO}}_{2}$) and is therefore similarly defined as

$$\mathrm{rCu}=\frac{c_{r}({\mathrm{CuSO}}_{4})}{c_{r}({\mathrm{CuSO}}_{4})+c_{r}({\mathrm{NiSO}}_{4})},$$ (2)

with the respective concentrations of the sulfate solutions relative to their blood mimicking base solutions

$$c_{r}({\mathrm{CuSO}}_{4})=\frac{c({\mathrm{CuSO}}_{4})}{c_{\mathrm{wb}}({\mathrm{CuSO}}_{4})}\;\text{and}\;c_{r}({\mathrm{NiSO}}_{4})=\frac{c({\mathrm{NiSO}}_{4})}{c_{\mathrm{wb}}({\mathrm{NiSO}}_{4})}.$$ (3)

For comparison, the definition of blood oxygen saturation is

$${\mathrm{sO}}_{2}=\frac{c({\mathrm{HbO}}_{2})}{c({\mathrm{HbO}}_{2})+c(\mathrm{Hb})}.$$ (4)

While of course not following hemoglobin spectra exactly, this sulfate model is a good qualitative fit to hemoglobin and is much easier to accurately control and reproduce than the saturation of oxygen in hemoglobin. It is highly stable over time; i.e., over 70 days only changes <1% in absorption were observed. Mimicking the blood volume fraction (BVF) in tissue, we define a sulfate volume fraction (SVF) in our model as $\mathrm{SVF}=c_{r}({\mathrm{CuSO}}_{4})+c_{r}({\mathrm{NiSO}}_{4})$. The SVF within the blood vessel mimicking tubing was always 100% mimicking whole blood, whereas the SVF in the background was varied as detailed in Sec. 2.3.3.

#### 2.3.2 Optical property reference measurements of phantoms

In the background medium, the scattering comparable to tissue (i.e., $\mu_{s}^{\prime }=15\;{\mathrm{cm}}^{-1}$ at 750 nm) was obtained using a 1.5% fat emulsion (diluted from SMOFlipid 20%, Fresenius Kabi, Switzerland).

To ensure a reproducible and tissue mimicking scattering, the background medium was analyzed with TCSPC spectroscopy. The TCSPC instrument used for the spectral analysis of the emulsions optical properties consisted of a white light supercontinuum laser (SuperK Extreme, NKT Photonics, Birkerød, Denmark) with ≈100  ps pulse duration (varying with wavelength), running at 39 MHz with <4  mW laser output. This white light was filtered by a tunable filter (SuperK Varia, NKT Photonics, Birkerød, Denmark), which was tuned in a range from 600 to 840 nm in 20 nm steps, with a bandwidth of 10 nm; 840 nm being the maximum of the tunable filter’s range. A single-photon avalanche diode (MDP PDM Series, Micro Photon Devices, Bolzano, Italy) was used to detect single photons. The diode has a prolonged dead time of ≈80  ns after a photon detection. Because of that, the photon detection rate was kept sufficiently low to make photon detection events during the dead time unlikely. We ensured a detection rate lower than $10^{5}\;s^{-1}$ (≪1/80  ns), making a correction for missed photons during the dead time unnecessary. The distributions of times of flight were recorded with single photon counting electronics (SPC-160, Becker & Hickl GmbH, Berlin, Germany). Source and detector fibers were fixed in blunted hypodermic needles for stability. The laser pulse shape, temporal dispersion in the optical fibers, and response of the detector were characterized in the overall instrument response function (IRF), yielding an FWHM of ≈140  ps overall, varying with wavelength. The source and detection fibers were placed perpendicular to the surface of the sample medium and immersed in the medium by 0.5 mm. To reduce the detection of early arriving photons, a carbon fiber mesh blocker was placed into the direct path, at a distance of 6 mm from the source fiber (dimension: 1 mm depth, 4 mm width, 0.4 mm thickness). We measured the SMOFlipid 1.5% medium in an 8 cm radius, 10 cm deep beaker, with the fibers at the center. This is a sufficiently large volume to be approximated as a semi-infinite medium for the analytic diffusion model. The resulting media were both measured with a source detector separation ρ=20  mm, for each wavelength until at least $10^{7}\text{ photons}$ were detected. For some wavelengths, the laser needed to be attenuated to keep the photon detection rate below $10^{5}\;s^{-1}$. This acquisition protocol ensured a high signal-to-noise ratio (SNR) and allowed us to fit our diffusion model only to late arriving photons where the diffusion approximation is more accurate. For the phantom experiments, two bottles of a new batch of SMOFlipid were used—both batches and bottles were measured independently prior to experiments to avoid hidden variations in the background medium.

An analytic diffusion model^29^ with an extrapolated boundary condition for a semi-infinite medium^30^^,^^31^ was convolved with the corresponding IRF for each wavelength λ. The results were then fitted to the measured histograms of the single photon arrival times, yielding a series of tuples ($\mu_{s}^{\prime \mathrm{SPC}}(\lambda )$, $\mu_{a}^{\mathrm{SPC}}(\lambda )$). Our tunable filter was limited in range to a maximum wavelength 840 nm but we needed credible $\mu_{s}^{\prime }$ values up to 980 nm for the optical forward simulations. Therefore, a generic tissue model [Eq. (5)] from the mcxyz framework^32^ was used to expand and define the scattering properties within the optical forward simulation:

$$\mu_{s}^{\prime }(\lambda )=\mu_{s500}^{\prime }\cdot (f_{\mathrm{ray}}\cdot {\left(\lambda /500\;\mathrm{nm}\right)}^{-4}+(1-f_{\text{ray}})\cdot {\left(\lambda /500\;\mathrm{nm}\right)}^{-b_{\mathrm{mie}}}),$$ (5)

**Fig. 3:**
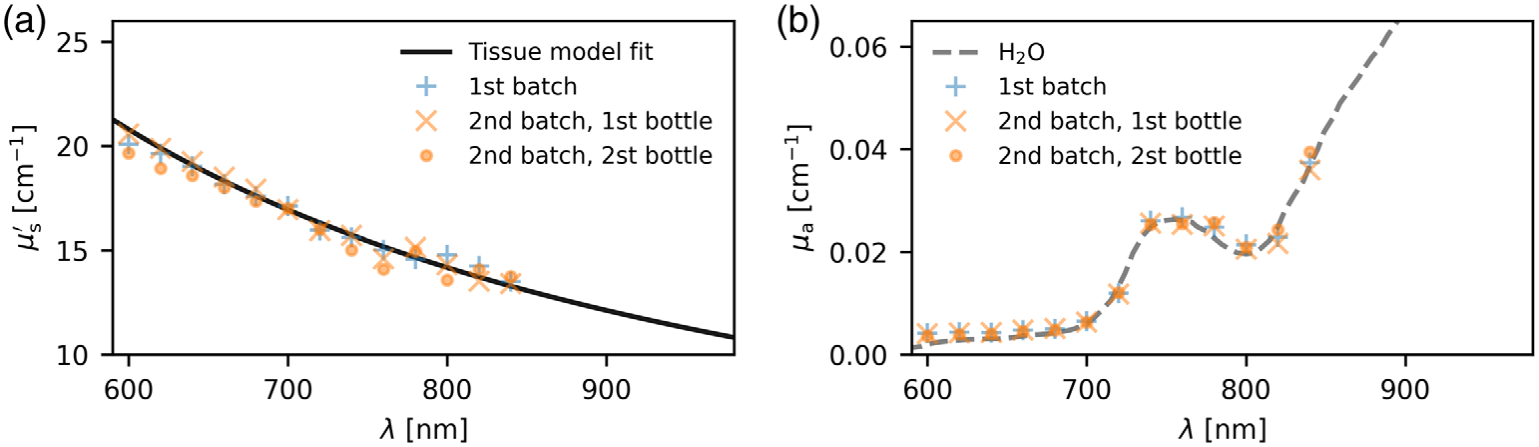
Optical properties of the uncolored phantom background medium. Single data points are diffusion model results from measurements with the TCSPC spectroscopy instrument of a 1.5% fat emulsion (diluted from SMOFlipid 20%). Each data point corresponds to a fit on a TCSPC histogram of at least $10^{7}\text{ photons}$ collected over at least 100 s. Validation phantoms cf. Fig. 4(a) were constructed with the “second batch, first bottle.” A generic tissue scattering model [Eq. (5)] fitted to this first bottle measurement was used to set the background scattering properties (a) for the Monte Carlo simulations. The “second bottle” was used for the background media in the test phantoms. The absorption results (b) are shown together with a literature spectrum of water absorption^33^ (dashed line).

With $\mu_{s500}^{\prime }=42.4\;{\mathrm{cm}}^{-1}$ the initial guess for $\mu_{s}^{\prime }$ at 500 nm, $f_{\text{ray}}=0.62$ the initial guess for fraction of Rayleigh scattering at 500 nm, and $b_{\mathrm{mie}}=1.0$ the initial guess for the scatter power for Mie scattering. This was fitted to the TCSPC data with a least squares fit—the entire data processing pipeline with all parameters is part of the open source code supplement (see the Code, Data, and Materials Availability section). The resulting fits are shown in Fig. 3.

#### 2.3.3 Phantom data sets

**Fig. 4:**
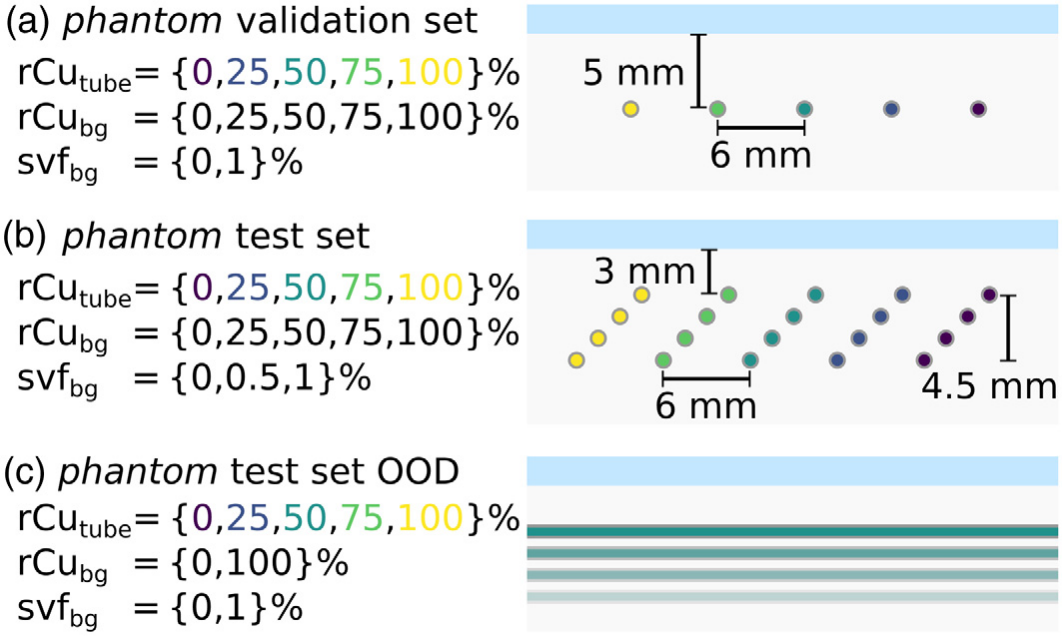
Cross sections of the phantom data sets, with denoted parameters: relative copper sulfate model in the tubes ${\mathrm{rCu}}_{\text{tube}}$, in the background medium ${\mathrm{rCu}}_{\mathrm{bg}}$ and sulfate model volume fraction in the background medium ${\mathrm{SVF}}_{\mathrm{bg}}$. (a) The validation phantoms with five single tubes. (b) The main test phantoms. (c) The test phantoms in longitudinal scan direction and thereby somewhat OOD of the training data. The shown two-dimensional cross sections correspond to the imaging plane. In sets A and B, the tubes run perpendicular to the imaging plane. Phantom test C has the same geometry as set B, with the imaging plane rotated by 90 deg to yield longitudinal scans instead of transversal scans of the tubes.

Three sets of phantoms (A,B,C) were produced, with different layout as shown in Fig. 4. All phantoms use polythene tubing filled with the relative copper sulfate model solution as target structures. The phantom backgrounds consist of a 1.5% fat emulsion with added sulfates.

**Fig. 5:**
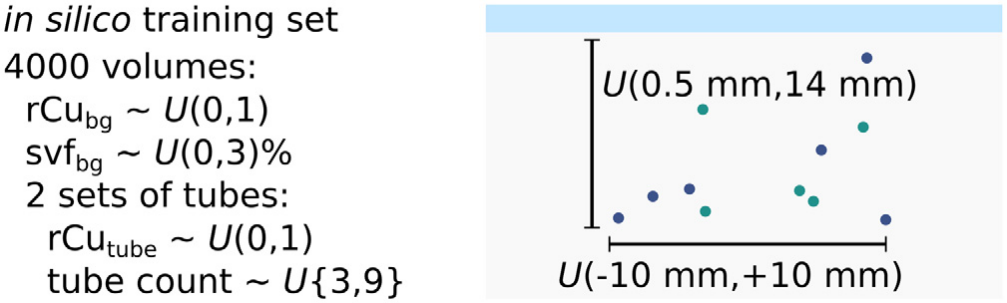
The *in silico* training data set consists of 4000 volumes, simulated with Monte Carlo simulations, modeling the geometry of the real MI setup. An additional 1000 volume test set is kept separate. Each volume has two sets of tubes each with a random number of tubes, uniformly distributed as specified. Tube and background relative copper (rCu) as well as background SVF are also drawn from uniform random distributions U. The ${\mathrm{sO}}_{2}$ training and test sets are simulated identically, substituting rCu absorption spectra for hemoglobin spectra, cf. Fig. 2.

Phantom layout A was measured as a validation data set for hyperparameter tuning of the machine learning models and validation of image reconstruction as well as parameter tuning in the Monte Carlo simulations. Layouts B and C were exclusively measured as test data sets. Phantom test set B is expected to be within the distribution of the simulation parameters (cf. Fig. 5). Phantom test set C, however, consists only of longitudinal scans w.r.t. the tube orientation. Because the orientation of the illumination positions changes with the imaging plane, set C was illuminated along the tubing. The measurements in set C are therefore expected to be out-of-distribution (OOD) with respect to the Monte Carlo simulated training sets. As detailed in the next section, the simulations were exclusively performed for transversal orientation of the tubing.

The phantom data sets contain 164 multispectral MI OA scans from 115 scan configurations as follows:

A. 30 scan configurations as laid out in Fig. 4(a): six phantom configurations, one with only a 1.5% SMOFlipid background solution and five with an added 1% SVF background with relative copper ${\mathrm{rCu}}_{\mathrm{bg}}$ set to {0, 25, 50, 75, 100}%. On each of these six configurations, five MI multispectral scans were performed centering the transducer on each of the tubes with ${\mathrm{rCu}}_{\text{tube}}=\{0,25,50,75,100\}\%$.
B. 55 scan configurations as laid out in Fig. 4(b): eleven phantom configurations, one with only a 1.5% SMOFlipid background solution, five with an added 1% SVF background with ${\mathrm{rCu}}_{\mathrm{bg}}=\{0,25,50,75,100\}\%$, and five with a 0.5% SVF. On these 11 phantom configurations, MI multispectral scans were performed centering on each of the five four-tube-arrays with ${\mathrm{rCu}}_{\text{tube}}=\{0,25,50,75,100\}\%$. For each four-tube-array, two regions of interest (ROI) (one containing the two lower and one the two upper tubes) were analyzed separately. The imaging plane was positioned for transversal scans of the tubes.
C. 30 scan configurations as laid out in Fig. 4(c): three phantom configurations, one with only a 1.5% SMOFlipid background solution, two with an added 1% SVF background with ${\mathrm{rCu}}_{\mathrm{bg}}=\{0,100\}\%$. On these three phantom configurations, MI multispectral scans were performed with each of the five shallowest tubes, and each of the five deepest tubes of the four-tube-arrays in the imaging plane, with ${\mathrm{rCu}}_{\text{tube}}=\{0,25,50,75,100\}\%$. The imaging plane was positioned for longitudinal scans of the tubes.

All scan configurations were scanned for 19.2 s yielding 30 MI and multispectral sequences. Due to the limited field of view of our US system (parallel read-out of 64 channels on a 19.2-mm linear array), we repositioned the probe between acquisitions—i.e., measuring five scan positions for phantom geometries A and B. The center of the linear transducer was always placed above the center of the targeted tubes. Scans with technical difficulties such as frame drops or wrong positioning were discarded in postprocessing. This affected one of the 115 scan configurations: the ${\mathrm{rCu}}_{\text{tube}}=100\%$, ${\mathrm{rCu}}_{\mathrm{bg}}=50\%$, SVF=0.5% was discarded for erroneous positioning. All scans of phantom geometry C were performed twice. The SVF=0 scans on phantom geometry B were performed five times on different days as a baseline measurement. The total phantom data set consists of 164 scans.

It is important to note that both copper and nickel sulfate act as a demulsifier when mixed with the diluted SMOFlipid background or any other fat in water emulsion. Phases will form and the bulk optical properties will change significantly within tens of seconds. To avoid the forming of phases, the background medium with added sulfates was continuously stirred with a magnetic stirrer during all the measurements.

### 2.4 Optical Forward Simulations

As an optical forward model, we used GPU accelerated Monte Carlo simulations to generate ground truth multispectral stacks of the absorbed energy distributions H(x,λ). Figure 5 shows the layout of the Monte Carlo simulated volumes.

To further illustrate that the rCu model is comparable to ${\mathrm{sO}}_{2}$, we performed all simulations twice: once for rCu model absorbers and once for hemoglobin. The ${\mathrm{sO}}_{2}$ sets are simulated substituting sulfate absorption spectra for whole blood concentration hemoglobin spectra, cf. Fig. 2. Each simulated data set consists of a 4000-volume training set and a separate 1000 volume test set. For each volume, 16 wavelength and four positions of illumination were simulated, modeled on the real MI OA imaging sequences. The simulations were performed with the open source mcx toolkit,^34^ and we used the ippai framework for the illumination modeling and data organization. In all data sets, each volume has two sets of tubes with the tube count drawn from a discrete uniform distribution U{3,9}, uniformly distributed in the volume as specified in Fig. 5. Tube and background rCu or ${\mathrm{sO}}_{2}$ are drawn from a continuous uniform random distribution U(0,1). All tubes were set to a radius of 0.4 mm. The wavelength-dependent background scattering parameters were set to the tissue model results from the fit of the TCSPC measurements to Eq. (5). The background SVF or BVF was drawn from U(0,3)%. Each simulation was performed with $10^{8}$ launched photon packets.

Running these simulations on a high performance computing cluster, we used mostly 1080 GTX (NVIDIA, Santa Clara) GPUs, with which a single wavelength and single illumination position simulation took ∼2  min. All simulations for the test and training sets used a combined 2 years of GPU time (one for the rCu sets and one for the ${\mathrm{sO}}_{2}$ sets). This was made feasible by usually running 40 GPUs in parallel. It should be noted that this seemingly excessive simulation time was chosen after simulation results with $10^{7}$ photon packets proved too noisy. This was evaluated prior to the presented *in silico* data sets. Initial hyperparameter tuning was also performed on two *in silico* data sets, simulated with $10^{7}$ photon packets. These data sets are part of the supplemental data (see the Code, Data, and Materials Availability section).

### 2.5 Machine Learning Algorithms

The estimation of an ${\mathrm{sO}}_{2}$ or rCu value from a measured spectrum is a regression problem. The usual approach to this problem in OA imaging is linear spectral unmixing (LU).^26^^,^^35^ For one pixel, the OA signal spectrum S(λ) is measured at a set of wavelengths λ. This sampled OA signal spectrum S is then fitted to a linear combination of known absorption spectra. Here, LU is performed numerically using an iterative least squares solver implemented in Python’s scipy.optimize submodule. These LU estimations (${\mathrm{rCu}}_{\mathrm{est}}^{\mathrm{LU}}$) are given throughout the results section as a reference.

We also compare our results to LSD, a type of machine learning algorithm. LSD also aims to estimate ${\mathrm{sO}}_{2}$ or rCu from the same single illumination OA signal spectra S measured at wavelengths λ. Similar to prior implementations, our modified LSD models are machine learning algorithms that are trained on large amounts of simulated absorbed energy spectra labeled with ground truth rCu. Before training, each absorbed energy spectrum is normalized with its L1 norm to H^(λ). This normalization makes them equivalent to a normalized OA signal spectrum S^(λ). This is because we can assume that for a signal spectrum S at a position $x_{0}$

$$S(x_{0},\lambda )=\Gamma (x_{0})\cdot A(x_{0})\cdot H(x_{0},\lambda )$$ (6)

⇒S^(x0,λ)≈H^(x0,λ). (7)

Assuming a linear acoustic reconstruction such as DAS, $A(x_{0})$ is a spatially varying but wavelength-independent factor introduced by the imperfect acoustic reconstruction, the instrument response, and the calibration. $\Gamma (x_{0})$, as a material property is also independent of the illumination wavelength.^27^ The LSD model, which was trained on the *in silico* training set tuples (H^,rCutube), is then presented (1) unseen *in silico* test set spectra H^ or (2) spectra S^ from an unseen phantom data test set to estimate the corresponding ${\mathrm{rCu}}_{\mathrm{est}}^{\mathrm{LSD}}$.

Note that A actually does depend on the fluence distribution $\phi (x,\mu_{a}(x,\lambda ),\mu_{s}^{\prime }(x,\lambda ))$. A varying optical wavelength may lead to different acoustic spectra of the OA signal corresponding to the same structure, due to different spatial distributions in the absorbed energy. Our assumption is that this effect is small compared with the spectral coloring introduced directly by the fluence term in

$$H(x_{0},\lambda )=\phi (x_{0},\mu_{a}(x,\lambda ),\mu_{s}^{\prime }(x,\lambda ))\cdot \mu_{a}(x_{0},\lambda ).$$ (8)

**Fig. 6:**
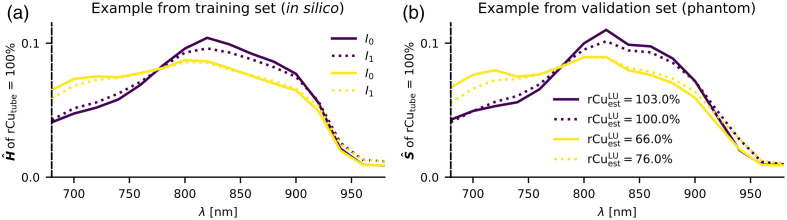
Examples for spectral coloring in L1 normalized spectra of an absorber with ${\mathrm{rCu}}_{\text{tube}}=100\%$. Comparison between (a) *in silico* absorbed energy spectra H^ and (b) phantom OA signal spectra S^. Spectra for two background media are shown: ${\mathrm{rCu}}_{\mathrm{bg}}=100\%$ (yellow), ${\mathrm{rCu}}_{\mathrm{bg}}=0\%$ (dark); with SVF=1%. The spectra for two illumination positions $I_{0}$ (line) and $I_{1}$ (dotted) are shown as an example for two of the four illuminations. Systematic changes in the spectral coloring can be seen for different background media and illumination positions. These changes are qualitatively similar for H^ and S^. On the validation phantom examples LU estimations for single spectra ${\mathrm{rCu}}_{\mathrm{est}}^{\mathrm{LU}}$ are listed—spectral coloring can cause large estimation errors relative to the ${\mathrm{rCu}}_{\text{tube}}=100\%$ ground truth.

For MI-LSD, we have multiple such normalized spectra S^ as input variables. For illustration, Fig. 6 shows spectra of the same pixel in an absorber with ${\mathrm{rCu}}_{\text{tube}}=100\%$ with two example illuminations $I_{0}$, $I_{1}$ and for two backgrounds with ${\mathrm{rCu}}_{\mathrm{bg}}=\{0\%,100\%\}$ and SVF=1%. The difference in background absorption causes a different spectral coloring but so does a variation of the illumination position. We hypothesize that training our machine learning algorithms on, i.e., four such spectra will allow us a more accurate and/or more robust estimation compared with LU and LSD.

Two types of machine learning algorithms were employed for spectral decoloring: feed forward neural networks (NN) and random forests (RF). Training of the MI-LSD models includes mirrored illumination positions for each volume as a minor data augmentation. Sorting the training data illumination position spectra stacks by their L1 norm before training was also investigated but did not prove beneficial on the validation data.

#### 2.5.1 Feedforward neural networks

Feedforward NNs were previously used for LSD implementations.^15^^,^^19^ We used this state-of-the-art NN architecture as a starting point and further tuned the hyperparameters on the training and validation sets. Doing so we mainly found the dropout layers of previous implementations to be counterproductive—dropout leading to a much lower precision on the validation set. The two final NNs used for both LSD and MI-LSD consisted of four hidden layers with twice the size of the input layer (16 for LSD and 64 for MI-LSD), all with leaky ReLu activation layers (and for comparison and dropout layers). For comparison to the previous implementation, additional results for a dropout in the dropout layers with probability p=0.2 are presented in Figs. S35–S50 and S66–S72 in the Supplementary Material. In the main results, no dropout was used (p=0). We segmented all vessels in the 4000 volume training set and trained on the segmented 1,052,152 simulated MI signal spectra for 100 epochs. As in the previous implementation, we used a batch size of $10^{5}$ and a learning rate of $10^{-2}\cdot 0.9^{\text{epoch}/2}$. All implementations are documented in the open source appendix. The trained models are also available in the open data appendix. We trained the algorithms on an RTX 2060 Super GPU (Nvidia, Santa Clara) and used the CPU for inference.

#### 2.5.2 Random forest regression

We also investigated RF regression,^36^ usually a highly accurate learning algorithm for regression problems with few dimensions.^8^ RFs are also usually less impacted by noise models. In particular, they should not overfit to noise.^36^ This should prove useful as we did not try to model a realistic wavelength-dependent noise. We used the Python scikit-learn v0.23 implementation of RF regressors using 100 trees with a maximum depth of 30 to limit memory consumption. Further parameters were set to default.

## 3 Results

We first show some qualitative comparisons between *in silico* rCu and phantom data and then present the performance of our trained RF and NN models on our *in silico* rCu test set and the two phantom test sets.

The hyperparameters of the machine learning models were tuned on the phantom validation set. The rCu machine learning models that performed best on our validation data were used to estimate rCu from the test sets. These models are presented in the results. For further information, all estimations for all models (on the validation set and for every single test measurement) can be found in the figures in the Supplementary Material; representative examples are shown here.

We trained the same RF and NN models, using the same hyperparameters, on an additional ${\mathrm{sO}}_{2}$ training set.

We compare MI-LSD with LSD and LU. Comparing a method based on a single measurement with a method based on multiple such measurements, the multiple measurement method should generally be more accurate simply due to an increase in SNR. To more fairly compare MI-LSD with the single spectrum methods such as LU and LSD, we estimated LSD and LU results on the reconstructed signals, averaged over the four illuminations. Using this averaged illumination spectrum as input for LU and LSD, we can compare methods for the same delivered energy during the same time—giving no method an inherent SNR advantage. LSD was also trained on *in silico* data using the same averaged illumination spectra from four simulated illuminations.

**Fig. 7:**
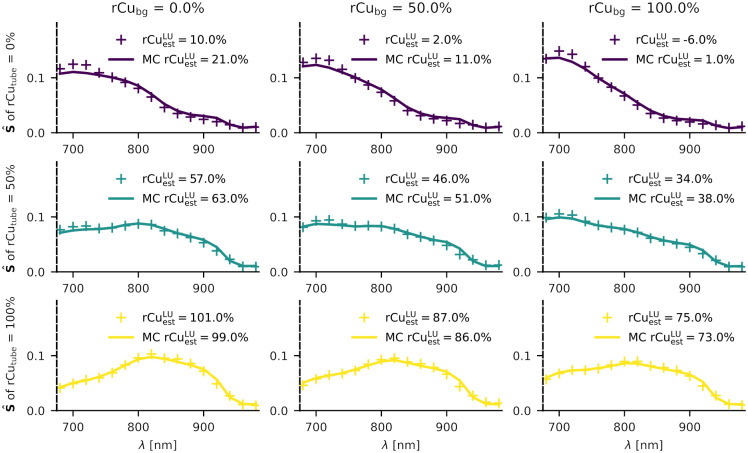
Qualitative comparison between spectra in a validation phantom (for SVF=0) and its digital twin from Monte Carlo (MC) simulations, showing the effects of various spectral coloring on the mean illumination spectra. Relative copper ${\mathrm{rCu}}_{\text{tube}}$ is varied in the target tube (up-down) and the background medium ${\mathrm{rCu}}_{\mathrm{bg}}$ (left-right). For reference, linear unmixing (LU) rCu estimates are given for each spectrum.

In addition to using the validation data set for hyperparameter tuning, we also qualitatively compared a set of our measurements with Monte Carlo simulations of one of the validation phantoms, creating an exact *in silico* representation of the light propagation in the validation phantom. Figure 7 serves as a qualitative (phantom to *in silico*) comparison for some of the averaged illumination spectra.

**Fig. 8:**
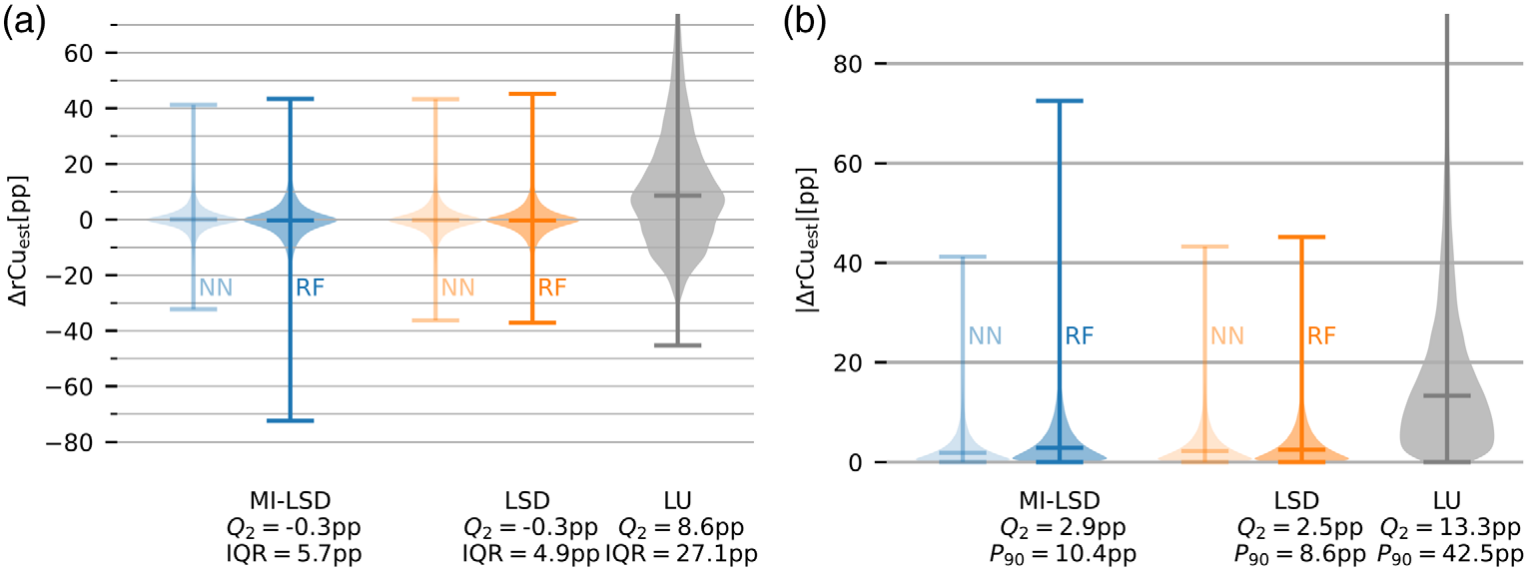
Error distributions of the *in silico* rCu test set cf. Fig. 5. (a) rCu estimation errors $\Delta {\mathrm{rCu}}_{\mathrm{est}}$ and (b) their absolutes. Blue shows the rCu estimators using MI-LSD, orange the estimators using LSD, and gray is the LU reference. Medians $Q_{2}$ of the error distributions are shown, together with interquartile ranges (IQR) and 90th percentiles $P_{90}$. The two feedforward NN models and the two RF models all have median absolute errors below 3 pp.

**Fig. 9:**
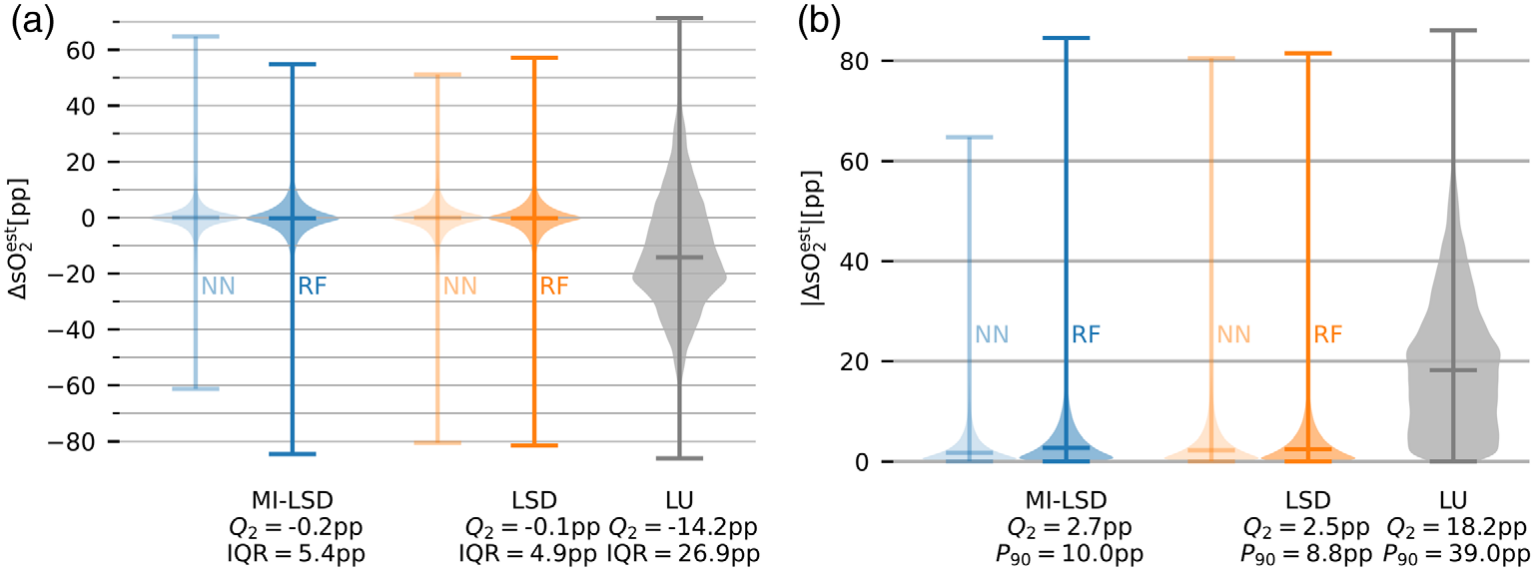
Error distributions of the *in silico* ${\mathrm{sO}}_{2}$ test set cf. Fig. 5. (a) ${\mathrm{sO}}_{2}$ estimation errors $\Delta {\mathrm{sO}}_{2}^{\mathrm{est}}$ and (b) their absolutes. Blue shows the ${\mathrm{sO}}_{2}$ estimators using MI-LSD, orange the estimators using LSD and gray is the LU reference. Medians $Q_{2}$ of the error distributions are shown, together with IQR and 90th percentiles $P_{90}$. The two feedforward NN models and the two RF models all have median absolute errors below 3 pp.

We report the estimation error distributions on the three distinct test sets. Reported are rCu estimation errors $\Delta {\mathrm{rCu}}_{\mathrm{est}}={\mathrm{rCu}}_{\mathrm{est}}-{\mathrm{rCu}}_{\text{tube}}$ and their absolutes $\left|\Delta {\mathrm{rCu}}_{\mathrm{est}}\right|$, with ${\mathrm{rCu}}_{\text{tube}}$ being the ground truth rCu in the tube. In the *in silico* test set, all selected models are in close agreement. As shown in Fig. 8, both LSD and MI-LSD estimations of rCu with both RFs and NNs yield median $Q_{2}$ absolute estimation errors of <3 percentage points (pp). The same can be seen in Fig. 9 for the *in silico* ${\mathrm{sO}}_{2}$ test set. As expected, estimation with all used models is very fast compared to LU. Inference on CPU for all the 266,105 samples in the *in silico* test sets took 1.6 s for RF MI-LSD, 1.3 s for RF LSD, 0.2 s for NN MI-LSD, 0.04 s for NN LSD, compared to 642 s for LU.

**Fig. 10:**
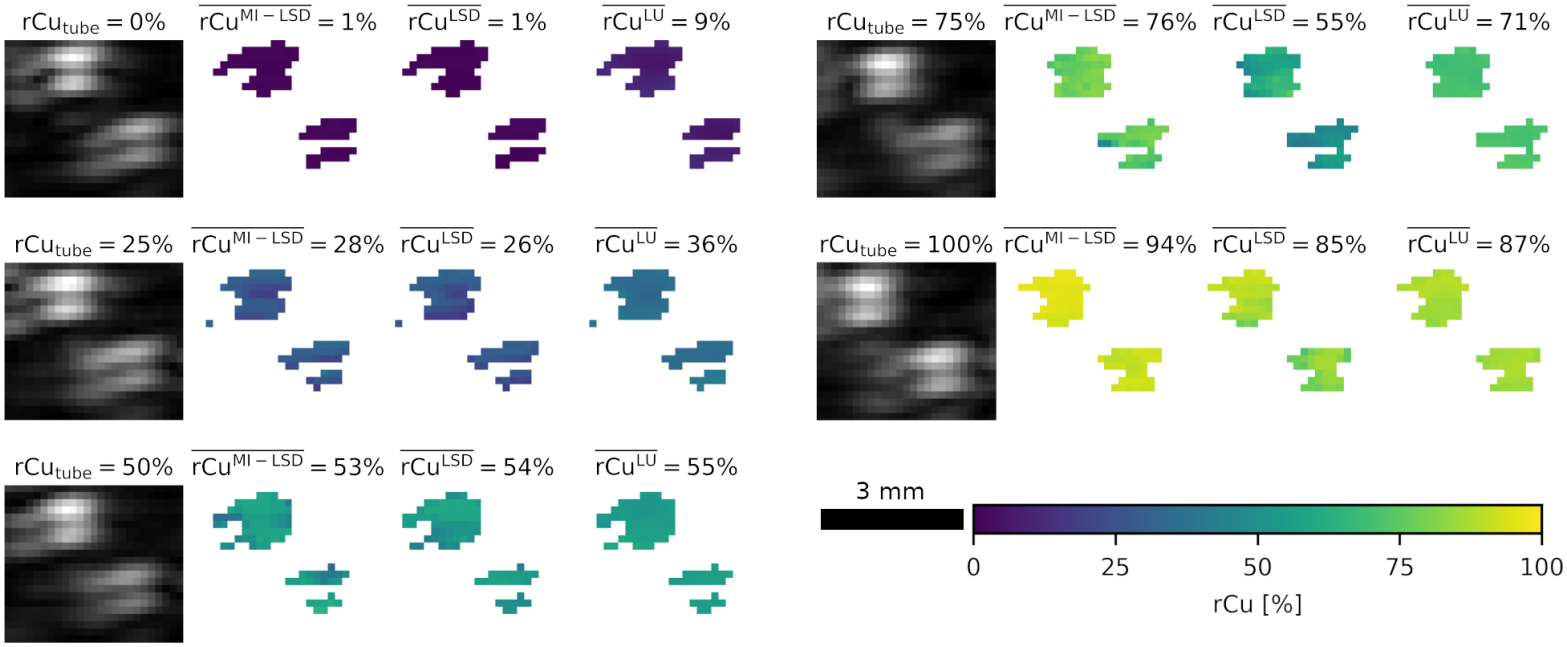
Example ROI in the phantom test set B with the estimation results for MI-LSD, LSD, and LU. Shown are the lower two tubes of a four tube phantom with SVF=0.5% and ${\mathrm{rCu}}_{\mathrm{bg}}=25\%$. To indicate the content of the ROI, the mean OA signal in the ROI is shown left, with the ground truth ${\mathrm{rCu}}_{\text{tube}}$ annotated. The brightness of the OA signal is independently and linearly autoleveled for each ROI. The mean rCu estimate $\overset{¯}{\mathrm{rCu}}$ over the ROI is noted for the three estimators.

From phantom test set B, tube signal was segmented by thresholding. In each reconstructed MI-multispectral OA image stack, two ROIs were chosen: one containing the two upper tubes and one containing the lower two tubes. One such lower tubes ROI is shown in Fig. 10. Each ROI has a fixed size of 3.75  mm×3.3  mm. The 15% highest mean (over all wavelengths and illuminations) OA signal pixels in each ROI were segmented as tube and rCu was estimated from the MI-multispectral OA signals in all pixels of these tube signal areas. The ROIs were thresholded separately to get an equal number of lower tube samples into the test set. A thresholding on the entire image or a larger ROI, using a lower cut-off percentage, would lead to more clutter and noise in the test set and the lower tubes being underrepresented in the test set.

**Fig. 11:**
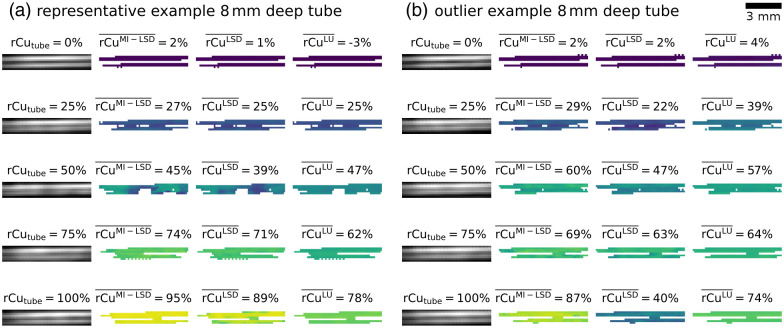
Example ROI 8 mm deep in the phantom test set C with estimation results for MI-LSD, LSD, and LU. Shown are two ranges of five imaged tubes with their ${\mathrm{rCu}}_{\text{tube}}$ annotated above their mean OA signal. The brightness of the OA signal is independently and linearly autoleveled for each ROI. The mean rCu estimate $\bar{\mathrm{rCu}}$ over the ROI is noted for the three estimators. The ROIs for two sets of phantoms are shown. (a) A representative result (${\mathrm{rCu}}_{\mathrm{bg}}=100\%$, 1% SVF background), (b) a result with outlier estimation errors (0% SVF background). LSD has highest estimation errors in deep vessels and in phantoms with no added sulfates in the background medium, i.e., in (b) for ${\mathrm{rCu}}_{\text{tube}}=100\%$.

From phantom test set C, the tube signal was segmented in a similar fashion: from each reconstructed MI-multispectral OA signal image stack an ROI of fixed size (7.5  mm×1.5  mm) was selected, containing either the upper tube or the lower tube. Two such lower tubes example ROIs are shown in Figs. 11(a) and 11(b) for varying reference ${\mathrm{rCu}}_{\text{tube}}$. Within these ROIs, the 50% highest mean (over all wavelengths and illuminations) OA signal pixels were segmented as tube. rCu was then estimated from the MI-multispectral OA signals in all pixels within these tube signal locations.

**Fig. 12:**
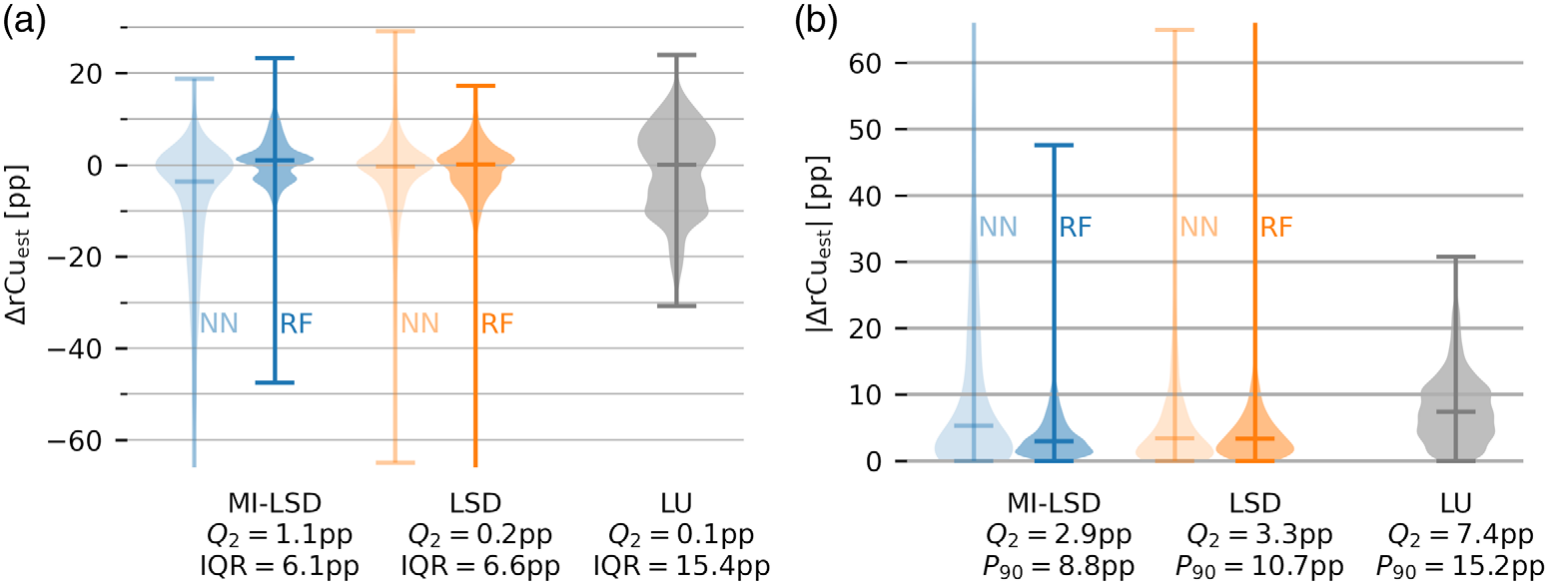
Error distributions of the phantom test set B [cf. Fig. 4(b)]. (a) rCu estimation errors $\Delta {\mathrm{rCu}}_{\mathrm{est}}$ and (b) their absolutes. Blue shows the rCu estimators using MI-LSD, orange the estimators using LSD, and gray is the LU reference. Medians $Q_{2}$ of the error distributions are shown, together with IQR and 90th percentiles $P_{90}$. The feedforward NN models performed similar to the RF models for the LSD method but underperformed for MI-LSD.

The estimated rCu image examples from the test sets are shown for the RF models, because with the exception of the *in silico* test set, NN models performed similarly or worse than RF models. For all estimated rCu images from all models, see the figures in the Supplementary Material. The error distributions for phantom test set B are shown in Fig. 12 and for phantom test set C in Fig. 12. Descriptive statistics of the relative error distributions in the estimated rCu data are reported in Table 1 for the two phantom test sets B and C.

**Table 1:** Relative rCu estimation errors ($\Delta {\mathrm{rCu}}_{\mathrm{est}}$) and absolute rCu estimation errors ($\left|\Delta {\mathrm{rCu}}_{\mathrm{est}}\right|$) for the RFs, NNs, and linear unmixing. Mean, median $Q_{2}$, first and third quartiles $Q_{1}$ and $Q_{3}$, and the 90th percentile $P_{90}$ are listed for the phantom test sets B (transversal tubes) and C (longitudinal tubes).

		Set	$\Delta {\mathrm{rCu}}_{\mathrm{est}}$ (pp)				$\left|\Delta {\mathrm{rCu}}_{\mathrm{est}}\right|$ (pp)				
			Mean	$Q_{1}$	$Q_{2}$	$Q_{3}$	Mean	$Q_{1}$	$Q_{2}$	$Q_{3}$	$P_{90}$
RF	MI-LSD	B	0.6	−2.7	1.1	3.4	4.1	1.4	2.9	5.3	8.8
		C	1.8	−3.1	1.7	6.3	5.6	2.1	4.5	7.9	12.4
	LSD	B	−1.9	−4.4	0.2	2.2	5.2	1.5	3.3	6.2	10.7
		C	−2.8	−5.3	0.6	2.6	7.1	1.7	3.9	7.9	13.7
NN	MI-LSD	B	−11.3	−18.4	−3.6	0.3	12.8	1.3	5.3	18.4	36.2
		C	−21.0	−38.2	−12.0	0.1	22.0	2.2	12.0	38.2	58.1
	LSD	B	−3.1	−5.9	−0.3	1.8	6.4	1.1	3.4	8.1	16.7
		C	−8.7	−15.1	−2.6	1.3	11.4	1.7	5.8	15.7	32.6
LU		B	−1.2	−8.8	0.1	6.6	8.2	4.0	7.4	11.1	15.2
		C	−1.0	−8.0	−0.5	6.3	8.7	3.4	7.2	12.5	18.2

## 4 Discussion

The qualitative comparison of the absorbed energy spectra from the Monte Carlo simulations and the phantom OA signal spectra reveals a general agreement between the simulations and the phantom results. The existing variations between the normalized spectra of the two domains are likely due to discrepancies in the simulation, e.g., the beam profiles and the optical properties of the gel pad. The gel pad for example is currently simulated as water but also has some low-level scattering properties, which was omitted in the simulation. In addition, the realistic laser noise was not simulated and the phantom positioning was only accurate to a millimeter. An acoustic forward simulation (e.g., using k-wave) was also not included in the simulation pipeline due to computational time constraints. While there are some acoustic artifacts (e.g., reflection artifacts) in the real OA image reconstructions, it is sensible to assume that they do not vary for different wavelength illumination, therefore, their effect on spectral coloring should be negligible. Variations in the Grüneisen parameter were also not part of the training set, even though it does vary significantly with rCu, because $\Gamma (c_{\mathrm{wb}}({\mathrm{NiSO}}_{4}))\approx 0.21$ and $\Gamma (c_{\mathrm{wb}}({\mathrm{CuSO}}_{4}))\approx 0.14$ at room temperature.^27^ This results in a systematically higher SNR for low rCu—an effect not present in ${\mathrm{sO}}_{2}$,^37^ which may explain why high rCu estimations are systematically worse in all of our phantom test sets. Laser noise levels are also wavelength dependent, which is reinforced by the pulse energy correction, e.g., resulting in a factor two SNR when measuring at 800 nm compared with 680 nm.

Our MI-LSD method with RF estimators was highly accurate with median absolute estimation errors of only 2.9 and 4.5 pp in the two phantom test sets, respectively. Our NN models, however, failed to give accurate estimates for MI-LSD. LSD estimates using NN were only improved over the LU reference and only in the phantom test set B. When testing on the OOD test set C, our NN models showed no clear improvement over LU. This leads us to the initial conclusion that the overly complex NN models are prone to overfitting to the *in silico* data, even when optimizing their hyperparameters with simple phantom data. The attempt to remedy this with dropout layers lead to overall inaccurate estimations.

**Fig. 13:**
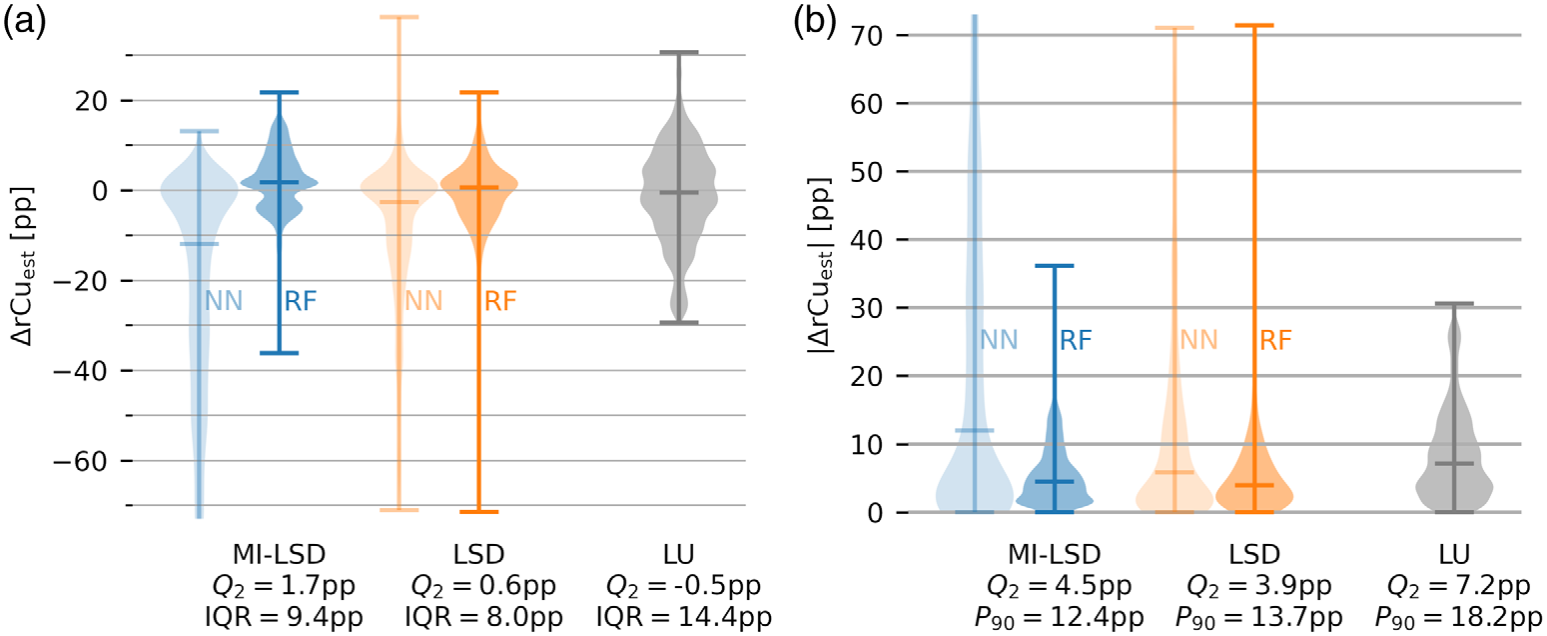
Error distributions of the phantom test set C [cf. Fig. 4(c)]. (a) rCu estimation errors $\Delta {\mathrm{rCu}}_{\mathrm{est}}$ and (b) their absolutes. Blue shows the rCu estimators using MI-LSD, orange the estimators using LSD, and gray is the LU reference. Medians $Q_{2}$ of the error distributions are shown, together with IQR and 90th percentiles $P_{90}$. The feedforward NN models performed similar to the RF models for the LSD method but underperformed for MI-LSD.

It is not surprising that the overall quantification performance was worse in deeper tubes. SNR in 8 mm deep tubes was very low, i.e., the longer distance illuminations with 980 nm light often yielded no detectable OA signal. This is due to background water absorption in combination with the high scattering, even when adding no sulfates to the background medium. We therefore also investigated omitting these higher wavelengths—training and testing with fewer wavelengths from 680 to 920 nm spaced 20 nm. This yielded obviously worse model performance overall, which either suggests that (these) 13 wavelengths are insufficient for accurate estimation or suggests that spectral coloring due to water absorption can be useful for a pixelwise correction of spectral coloring, as it can give implicit information on the optical path length. For further investigation, it may be useful to add explicit information on the pixel position to the input features. It may also be interesting to perform similar experiments with a wider range of and/or more lower wavelength measurements and then optimize the wavelength selection on these oversampled multispectral sequences. This was not done in this initial proof-of-concept work because it risks overoptimizing on unrealistic aspects of the rCu model (e.g., the difference in Grüneisen parameter of the two sulfate solutions) or setup specific aberrations (e.g., wavelength-dependent SNR). Simulating more wavelengths also prolongs the already computationally expensive, one GPU year, simulation time for the necessary training data (Fig. 13).

**Fig. 14:**
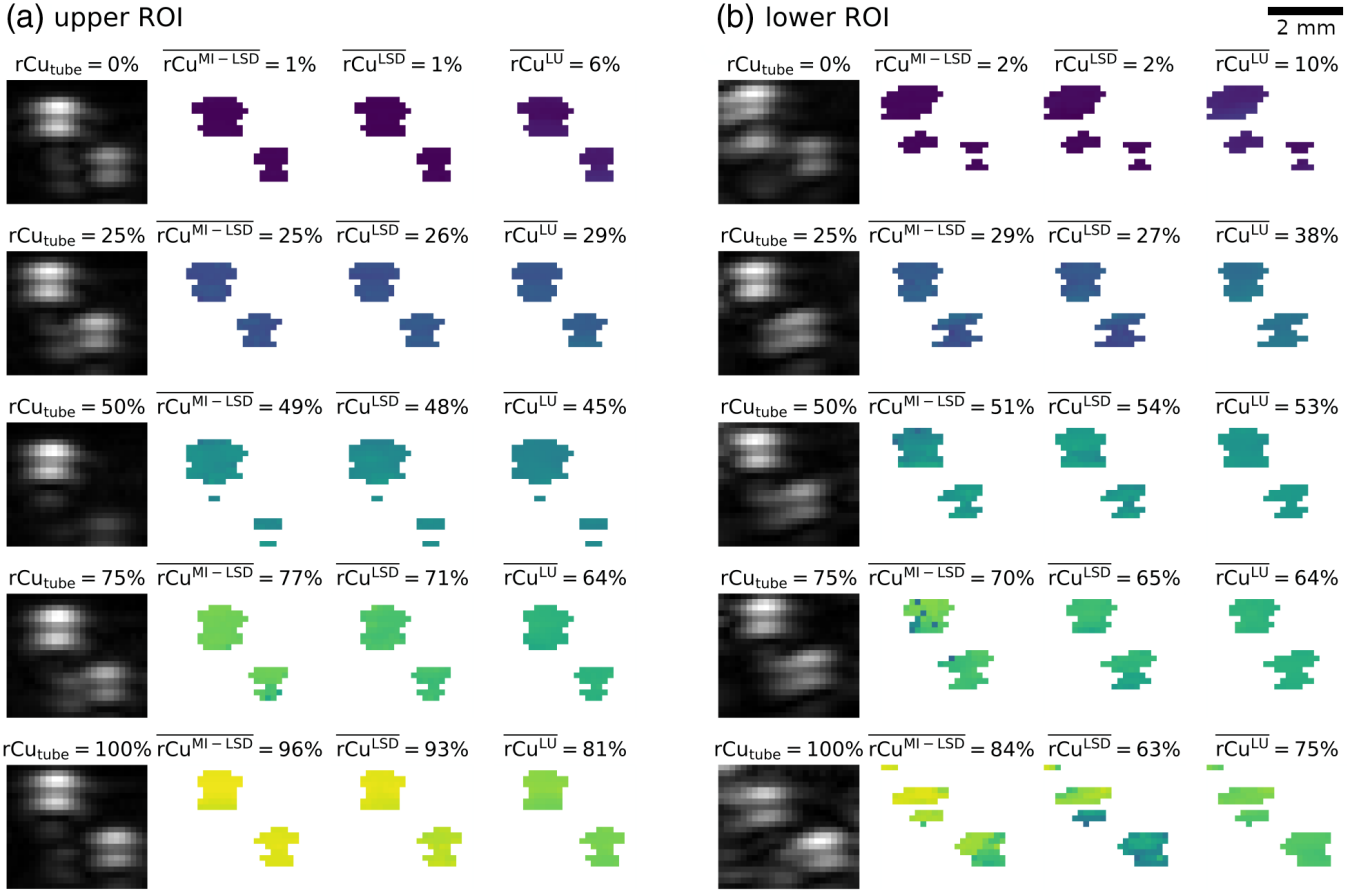
Worst estimation example in phantom test set B: deep tubes (b) compared with more shallow tubes (a) in the same phantom with an SVF=0%. Estimation results for MI-LSD, LSD, and LU. To indicate the content of the ROI, the mean OA signal in the ROI is shown left, with the ground truth ${\mathrm{rCu}}_{\text{tube}}$ annotated. The brightness of the OA signal is independently and linearly autoleveled for each ROI. The mean rCu estimate $\overset{¯}{\mathrm{rCu}}$ over the ROI is noted for the three estimators. Shallow tubes can be estimated very accurately while lower SNR in deep, high rCu tubes correlates to poor estimation accuracy.

A final somewhat surprising observation was that estimation of both LSD and to a lesser extent MI-LSD is poorest in phantoms with no added sulfates in the background medium. Figure 14(b) shows the worst estimation results in the lower tubes of phantom test set B—combining three detrimental circumstances: (1) great depth, (2) high rCu, and (3) only spectral coloring of water. Though even in this worst case, MI-LSD is more accurate than the LSD or LU estimations.

One of the main shortcomings of the presented phantom validation is that it did not model melanin absorption in skin. Spectral coloring by melanin still causes large errors in standard of care pulse oximetry devices^38^^,^^39^ and needs to be addressed for quantitative OA imaging. We were, however, not able to reproducibly include a skin mimicking layer with varying melanin absorption in our liquid phantoms—future work will address this, using solid, layered gel wax phantoms.^40^

We showed a proof-of-concept setup with comparably poor image quality due to the US DAQ and transducer. An *in vivo* applicable system should make use of state-of-the-art US components and further engineering improvements to sensitivity and SNR, as this currently further limits the achievable estimation accuracy for deep ROI using our setup. Wavelength selection and illumination geometry are suitable but their optimal choice was not the aim of this work. Lastly, while the rCu model is a very useful tool for the investigation and thorough validation of a quantitative OA oximetry method and while the MI-LSD approach shows similar results for *in silico* ${\mathrm{sO}}_{2}$, an explicit translation to actual ${\mathrm{sO}}_{2}$ estimation *in vivo* must be the next step. One of the main challenges for this translation will be the adequate modeling of additional chromophore distributions, such as melanin. Melanin will strongly affect both overall SNR and spectral coloring.

## 5 Conclusions

We presented MI-LSD, a quantitative OA oximetry method using MIs and machine learning; and presented a real-time MI OA imaging setup with a linear ultrasound transducer. We used this setup to image 115 phantom configurations by employing a highly reliable, reproducible, and easily scalable phantom model.

MI-LSD with RFs was able to accurately and quickly estimate blood oxygen saturation modeled by copper and nickel sulfate. Compared with LU, MI-LSD approximately halved the magnitude of the relative estimation error, achieving median absolute estimation errors of only 2.9 and 4.5 pp in our two phantom test sets, respectively. To investigate such ML regression methods, thorough phantom validation is critical, as *in silico* tests do not give sufficient data to validate a method, and *in vivo* measurements lack a reliable ground truth. This is further illustrated by the fact that previously reported LSD NN models, which were only validated on *in silico* data, slightly outperformed RF models on *in silico* data (as was previously reported) but underperformed RF models in phantom tests while simply breaking on OOD phantom data.

The results of this study give us a high degree of confidence that the domain gap from *in silico* spectral decoloring to real data can be bridged using MI-LSD, paving the way to a clinical application of quantitative OA oximetry imaging.

## 6 Code, Data, and Materials Availability

The code for the methods as well as the experiments was implemented in Python 3.7 and is fully open source, available at github:thkirchner/PA-MI-LSD. All training, validation, and test data sets generated in this work are openly available at doi:10.5281/zenodo.4549631. The raw Monte Carlo simulation results and raw OA scans are too large for upload – 3 TB – but available from the authors upon reasonable request.
