# Supplementary material for: Multiple illumination learned spectral decoloring for quantitative optoacoustic oximetry imaging
Source: J Biomed Opt. 2021 Aug 4;26(8):085001. doi: 10.1117/1.JBO.26.8.085001 (PMC8336722; doi:10.1117/1.JBO.26.8.085001)
Supplement: Supplementary file 1 [file JBO_026_085001_SD001.pdf]

Supplement – Quantitative photoacoustic oximetry imaging by multiple illumination learned spectral decoloring

Thomas Kirchner and Martin Frenz
Biomedical Photonics, Institute of Applied Physics, University of Bern, Bern, Switzerland.

This supplement presents all rCu estimation results for all data sets and for all models – random forests (RF) and neural networks (NN) with and without dropout. It also reports complete rCu estimation errors(ΔrCu^{est}) and absolute rCu estimation errors($|\Delta rCu^{est}|$) over all separate data sets in error distribution figures. The examples shown in the main manuscript are figure 12 right, 57 right, 54 right and 4.

Contents

1 in silico Sets **3**

2 Phantom Test Set B **4**
2.1 Random Forest (RF) 5
2.2 Feed Forward Neural Network (NN) – without dropout 13
2.3 Feed Forward Neural Network (NN) – with dropout 21

3 Phantom Test Set C **29**
3.1 Random Forest (RF) 29
3.2 Feed Forward Neural Network (NN) – without dropout 33
3.3 Feed Forward Neural Network (NN) – with dropout 36

4 Phantom Validation Set (A) **40**
4.1 Random Forest (RF) 40
4.2 Feed Forward Neural Network (NN) – without dropout 42
4.3 Feed Forward Neural Network (NN) – with dropout 44

List of Figures

1 Error distribution *in silico training* set 3
2 Error distribution *in silico test* set 4
3 Error distribution transversal phantom *test* set 4
4 RF rCu estimates baseline 0 5
5 RF rCu estimates baseline 1 6
6 RF rCu estimates baseline 2 6
7 RF rCu estimates baseline 3 7
8 RF rCu estimates baseline 4 7
9 RF rCu estimates for background rCu 100%, svf=0.5% 8
10 RF rCu estimates for background rCu 75%, svf=0.5% 8
11 RF rCu estimates for background rCu 50%, svf=0.5% 9
12 RF rCu estimates for background rCu 25%, svf=0.5% 9
13 RF rCu estimates for background rCu 0%, svf=0.5% 10
14 RF rCu estimates for background rCu 100%, svf=1% 10
15 RF rCu estimates for background rCu 75%, svf=1% 11
16 RF rCu estimates for background rCu 50%, svf=1% 11
17 RF rCu estimates for background rCu 25%, svf=1% 12
18 RF rCu estimates for background rCu 0%, svf=1% 12
19 NN (no dropout) error distribution transversal phantom *test* set 13
20 NN (no dropout), rCu estimates baseline 0 13
21 NN (no dropout), rCu estimates baseline 1 14
22 NN (no dropout), rCu estimates baseline 2 14
23 NN (no dropout), rCu estimates baseline 3 15
24 NN (no dropout), rCu estimates baseline 4 15
25 NN (no dropout), rCu estimates for background rCu 100%, svf=0.5% 16
26 NN (no dropout), rCu estimates for background rCu 75%, svf=0.5% 16
27 NN (no dropout), rCu estimates for background rCu 50%, svf=0.5% 17
28 NN (no dropout), rCu estimates for background rCu 25%, svf=0.5% 17
29 NN (no dropout), rCu estimates for background rCu 0%, svf=0.5% 18
30 NN (no dropout), rCu estimates for background rCu 100%, svf=1% 18

31 NN (no dropout), rCu estimates for background rCu 75%, svf=1% 19
32 NN (no dropout), rCu estimates for background rCu 50%, svf=1% 19
33 NN (no dropout), rCu estimates for background rCu 25%, svf=1% 20
34 NN (no dropout), rCu estimates for background rCu 0%, svf=1% 20
35 NN (with dropout) error distribution transversal phantom *test* set 21
36 NN (with dropout), rCu estimates baseline 0 21
37 NN (with dropout), rCu estimates baseline 1 22
38 NN (with dropout), rCu estimates baseline 2 22
39 NN (with dropout), rCu estimates baseline 3 23
40 NN (with dropout), rCu estimates baseline 4 23
41 NN (with dropout), rCu estimates for background rCu 100%, svf=0.5% 24
42 NN (with dropout), rCu estimates for background rCu 75%, svf=0.5% 24
43 NN (with dropout), rCu estimates for background rCu 50%, svf=0.5% 25
44 NN (with dropout), rCu estimates for background rCu 25%, svf=0.5% 25
45 NN (with dropout), rCu estimates for background rCu 0%, svf=0.5% 26
46 NN (with dropout), rCu estimates for background rCu 100%, svf=1% 26
47 NN (with dropout), rCu estimates for background rCu 75%, svf=1% 27
48 NN (with dropout), rCu estimates for background rCu 50%, svf=1% 27
49 NN (with dropout), rCu estimates for background rCu 25%, svf=1% 28
50 NN (with dropout), rCu estimates for background rCu 0%, svf=1% 28
51 Error distribution longitudinal phantom *test* set 29
52 RF error distribution longitudinal phantom *test* set 29
53 RF rCu estimates for longitudinal svf=0% 30
54 RF rCu estimates for longitudinal svf=0% 30
55 RF rCu estimates for longitudinal svf=1.0%, background rCu=0% 31
56 RF rCu estimates for longitudinal svf=1.0%, background rCu=0% 31
57 RF rCu estimates for longitudinal svf=1.0%, background rCu=100% 32
58 RF rCu estimates for longitudinal svf=1.0%, background rCu=100% 32
59 NN (no dropout) error distribution longitudinal phantom *test* set 33
60 NN (no dropout) rCu estimates for longitudinal svf=0% 33
61 NN (no dropout) rCu estimates for longitudinal svf=0% 34
62 NN (no dropout) rCu estimates for longitudinal svf=1.0%, background rCu=0% 34
63 NN (no dropout) rCu estimates for longitudinal svf=1.0%, background rCu=0% 35
64 NN (no dropout) rCu estimates for longitudinal svf=1.0%, background rCu=100% 35
65 NN (no dropout) rCu estimates for longitudinal svf=1.0%, background rCu=100% 36
66 NN (with dropout) error distribution longitudinal phantom *test* set 36
67 NN (with dropout) rCu estimates for longitudinal svf=0% 37
68 NN (with dropout) rCu estimates for longitudinal svf=0% 37
69 NN (with dropout) rCu estimates for longitudinal svf=1.0%, background rCu=0% 38
70 NN (with dropout) rCu estimates for longitudinal svf=1.0%, background rCu=0% 38
71 NN (with dropout) rCu estimates for longitudinal svf=1.0%, background rCu=100% 39
72 NN (with dropout) rCu estimates for longitudinal svf=1.0%, background rCu=100% 39
73 Error distributions *validation* phantom 40
74 RF Error distribution *validation* phantom 40
75 RF estimates on validation phantom, rCu = (100, 75, 50, 25)% 41
76 RF estimates on validation phantom, rCu = 0% 42
77 NN (no dropout) Error distribution *validation* phantom 42
78 NN (no dropout) estimates on validation phantom, rCu = (100, 75, 50, 25)% 43
79 NN (no dropout) estimates on validation phantom, rCu = 0% 44
80 NN (with dropout) Error distribution *validation* phantom 44
81 NN (with dropout) estimates on validation phantom, rCu = (100, 75, 50, 25)% 45
82 NN (with dropout) estimates on validation phantom, rCu = 0% 46

List of Tables

1 Relative rCu estimation errors($\Delta \mathrm{rCu}^{\mathrm{est}}$) and absolute rCu estimation errors($|\Delta \mathrm{rCu}^{\mathrm{est}}|$) for the random forests (RF), neural networks (NN) and linear unmixing. Mean, median Q_2, 1st and 3rd quartiles Q_1 and Q_3, and the 90 percentile P_{90} are listed for the phantom test sets B (transversal tubes) and C (longitudinal tubes). 3

		set	ΔrCu^{est} [p.p.]				$\|\Delta$rCu$^{est}\|$ [p.p.]				
			mean	Q_1	Q_2	Q_3	mean	Q_1	Q_2	Q_3	P_{90}
RF	MI-LSD	B	0.6	-2.7	1.1	3.4	4.1	1.4	2.9	5.3	8.8
		C	1.8	-3.1	1.7	6.3	5.6	2.1	4.5	7.9	12.4
	LSD	B	-1.9	-4.4	0.2	2.2	5.2	1.5	3.3	6.2	10.7
		C	-2.8	-5.3	0.6	2.6	7.1	1.7	3.9	7.9	13.7
NN	MI-LSD	B	-11.3	-18.4	-3.6	0.3	12.8	1.3	5.3	18.4	36.2
		C	-21.0	-38.2	-12.0	0.1	22.0	2.2	12.0	38.2	58.1
	LSD	B	-3.1	-5.9	-0.3	1.8	6.4	1.1	3.4	8.1	16.7
		C	-8.7	-15.1	-2.6	1.3	11.4	1.7	5.8	15.7	32.6
LU		B	-1.2	-8.8	0.1	6.6	8.2	4.0	7.4	11.1	15.2
		C	-1.0	-8.0	-0.5	6.3	8.7	3.4	7.2	12.5	18.2

Table 1: Relative rCu estimation errors(ΔrCu^{est}) and absolute rCu estimation errors($|\Delta$rCu$^{est}|$) for the random forests (RF), neural networks (NN) and linear unmixing. Mean, median Q_2, 1st and 3rd quartiles Q_1 and Q_3, and the 90 percentile P_{90} are listed for the phantom test sets B (transversal tubes) and C (longitudinal tubes).

1 in silico Sets

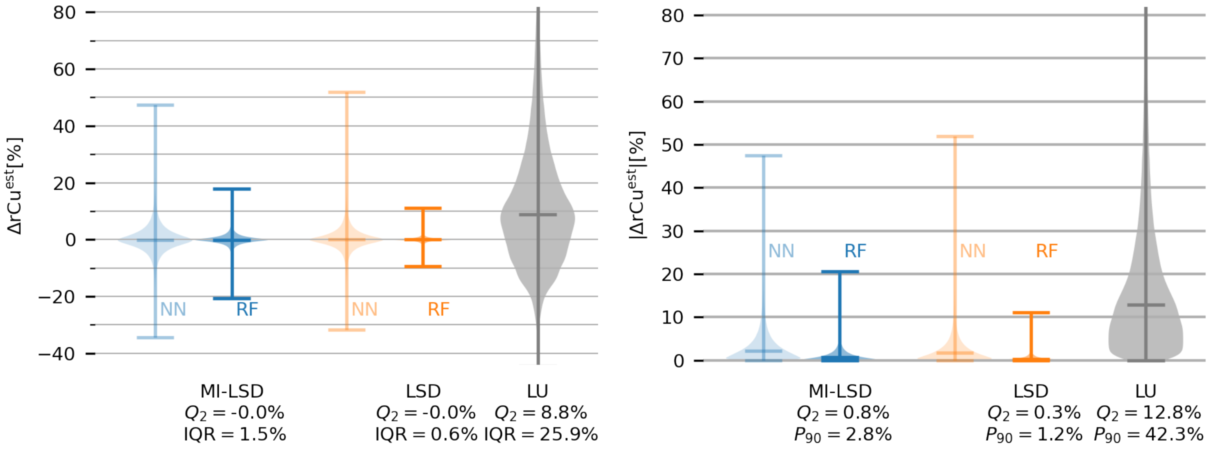

Figure 1: Estimation error distribution on the *in silico training* set. rCu estimation errors (ΔrCu^{est}) are shown left, their absolutes right. Blue shows the rCu estimators trained with multiple illumination learned spectral decoloring (MI-LSD), orange the estimators trained with learned spectral decoloring (LSD) and gray is the linear spectral unmixing (LU) reference. Listed: Medians Q_2, interquartile ranges (IQR) and 90 percentiles P_{90}.

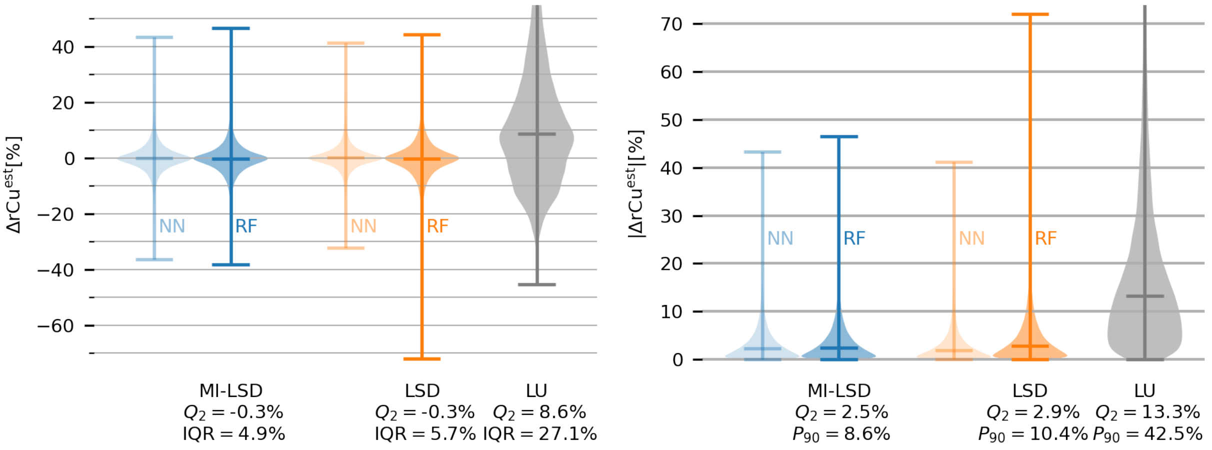

Figure 2: Estimation error distribution on the *in silico test* set. rCu estimation errors (ΔrCu^{est}) are shown left, their absolutes right. Blue shows the rCu estimators trained with multiple illumination learned spectral decoloring (MI-LSD), orange the estimators trained with learned spectral decoloring (LSD) and gray is the linear spectral unmixing (LU) reference. Listed: Medians Q_2, interquartile ranges (IQR) and 90 percentiles P_{90}.

2 Phantom Test Set B

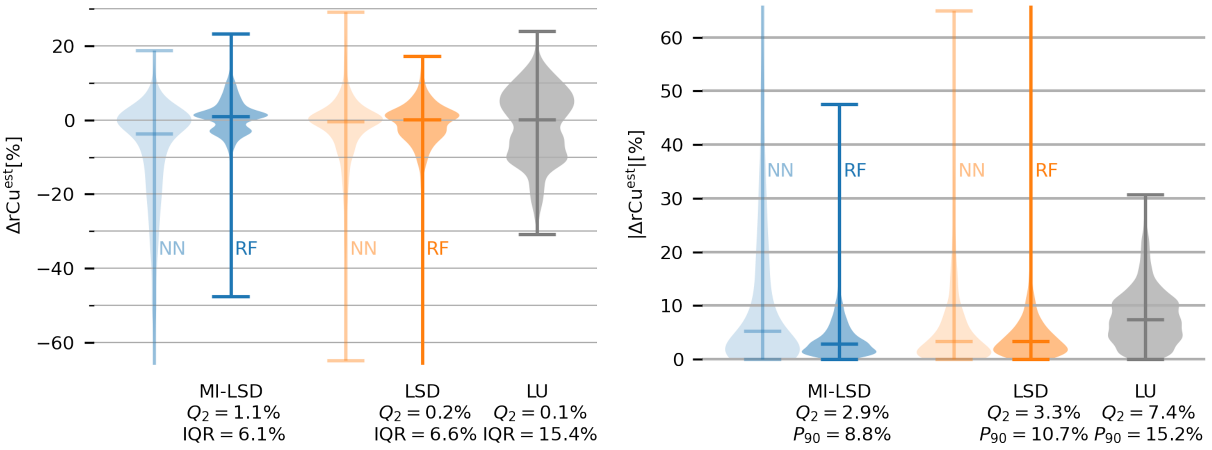

Figure 3: Estimation error distribution on the transversal phantom *test* set. rCu estimation errors (ΔrCu^{est}) are shown left, their absolutes right. Blue shows the rCu estimators trained with multiple illumination learned spectral decoloring (MI-LSD), orange the estimators trained with learned spectral decoloring (LSD) and gray is the linear spectral unmixing (LU) reference. Listed: Medians Q_2, interquartile ranges (IQR) and 90 percentiles P_{90}.

2.1 Random Forest (RF)

2.1.0.1 Baseline – 0% sulfate volume fraction (svf)

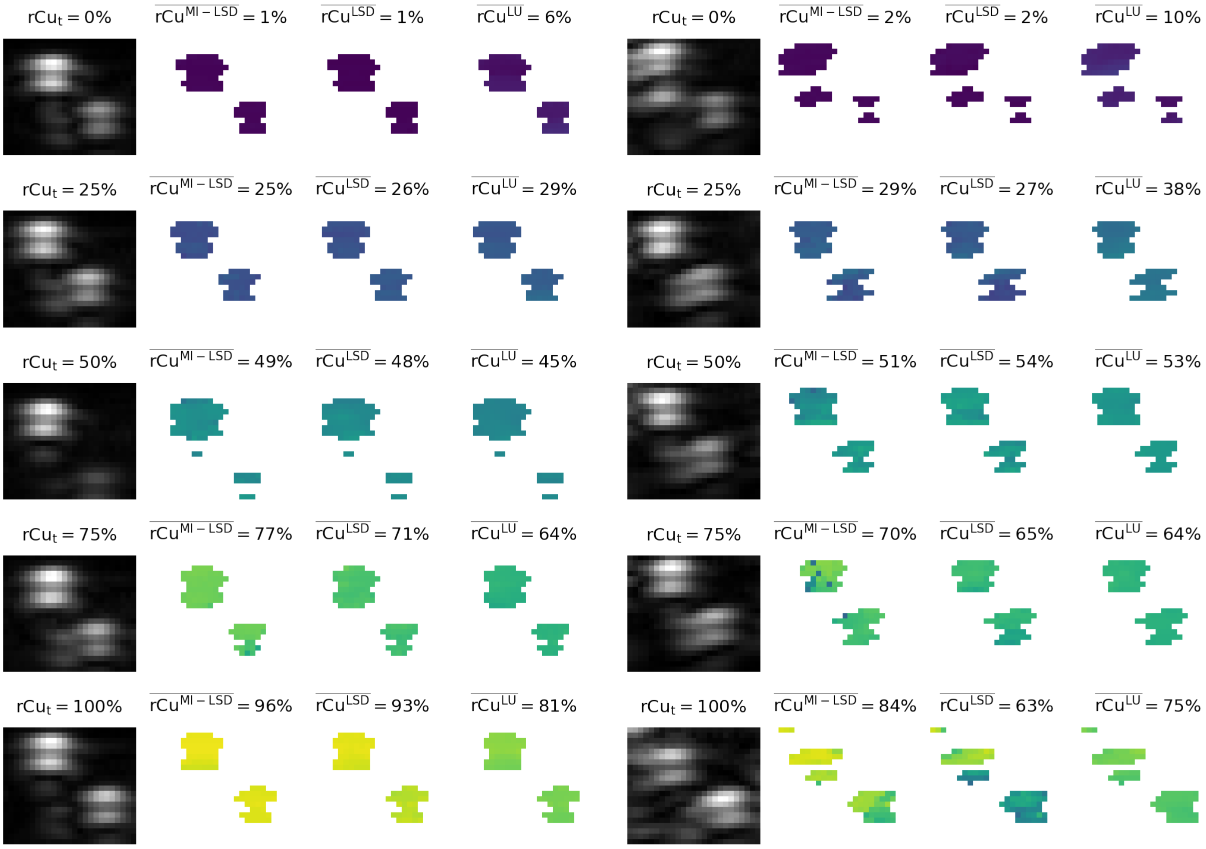

Figure 4: Baseline 0, RF – mean rCu estimates for Left: upper, Right: lower tubes. Showing mean signal and MI-LSD, LSD and LU estimates

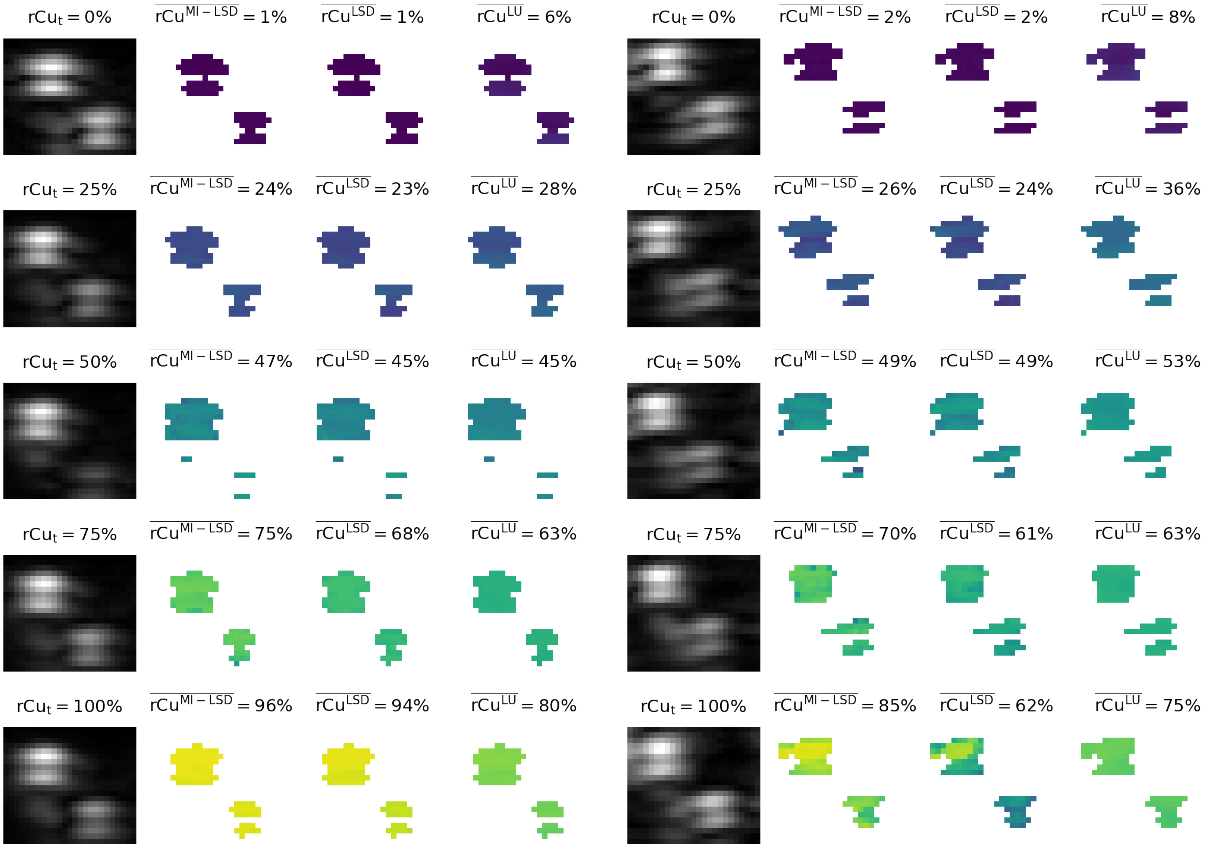

Figure 5: Baseline 1, RF – mean rCu estimates for Left: upper, Right: lower tubes. Showing mean signal and MI-LSD, LSD and LU estimates

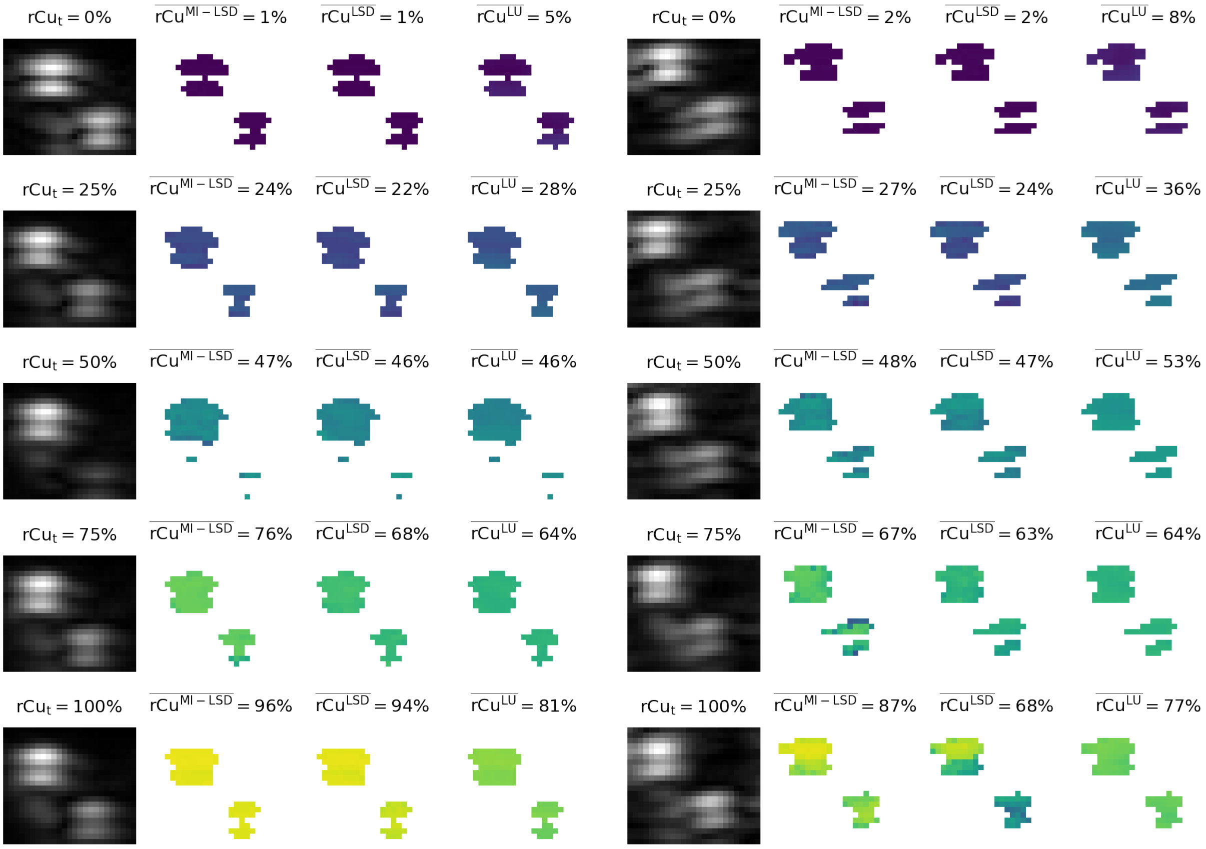

Figure 6: Baseline 2, RF – mean rCu estimates for Left: upper, Right: lower tubes. Showing mean signal and MI-LSD, LSD and LU estimates

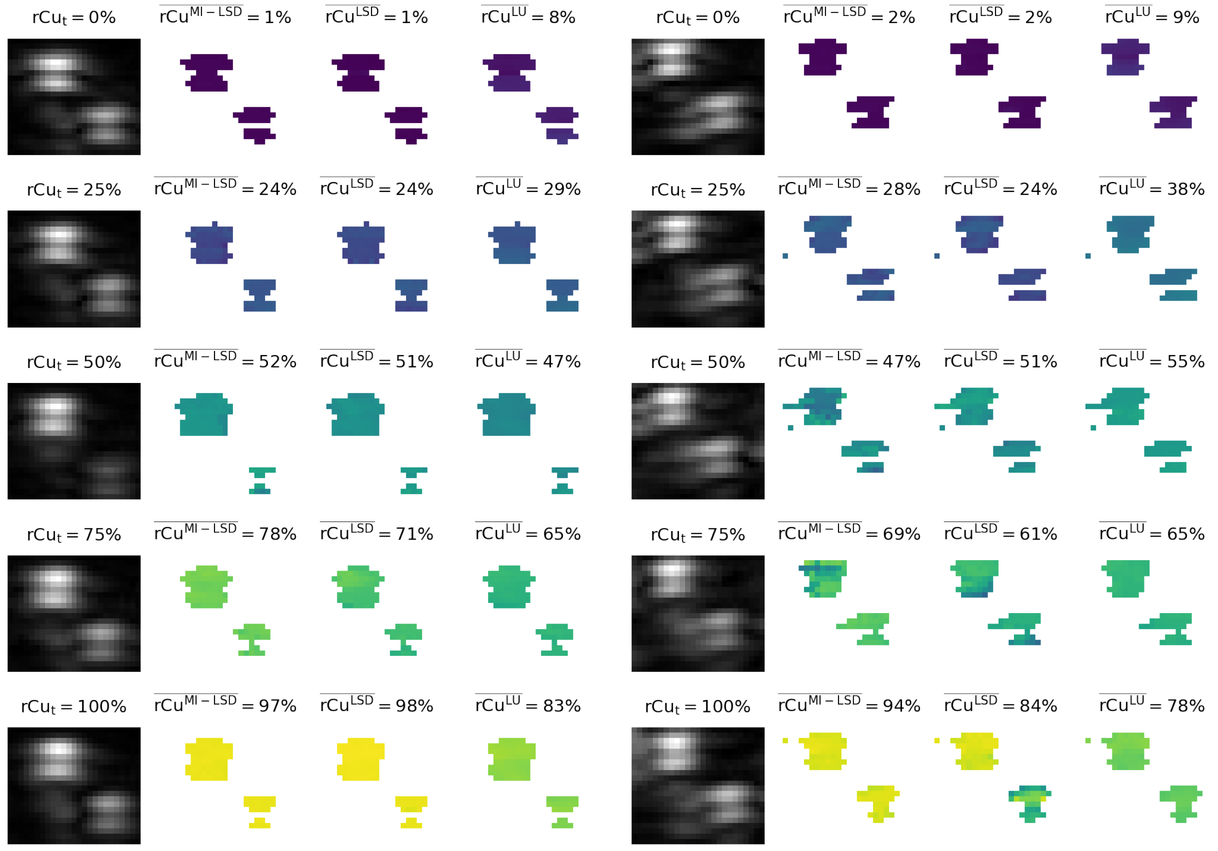

Figure 7: Baseline 3, RF – mean rCu estimates for Left: upper, Right: lower tubes. Showing mean signal and MI-LSD, LSD and LU estimates

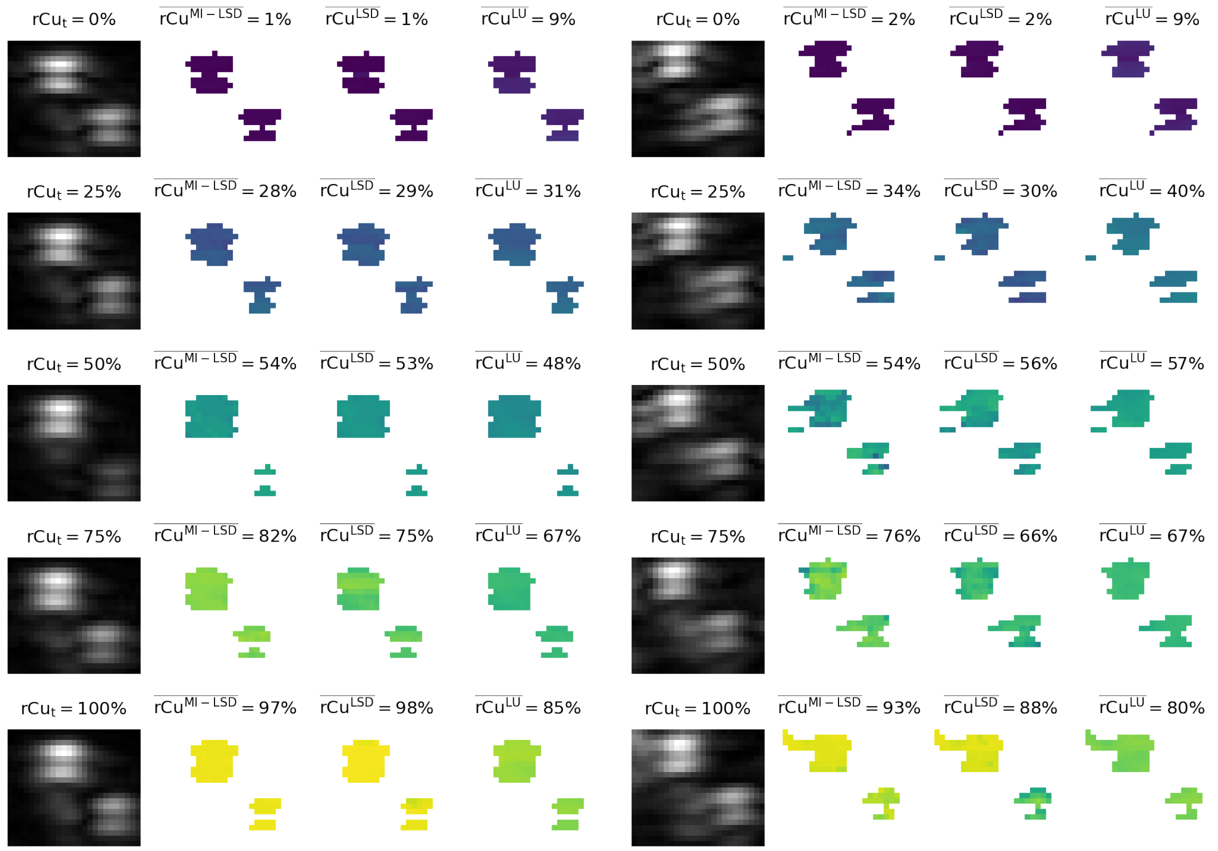

Figure 8: Baseline 4, RF – mean rCu estimates for Left: upper, Right: lower tubes. Showing mean signal and MI-LSD, LSD and LU estimates

2.1.0.2 0.5% sulfate volume fraction (svf)

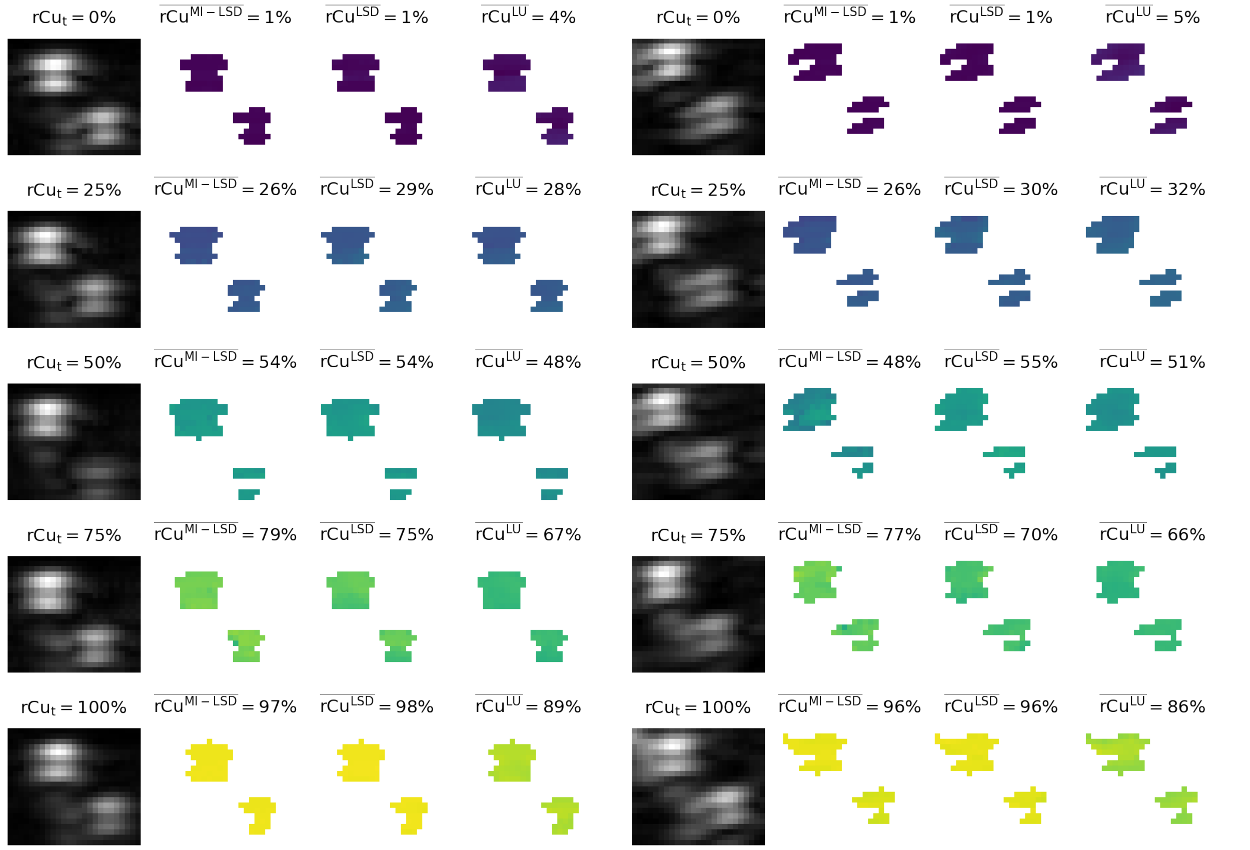

Figure 9: $rCu_{bg} = 100\%$, svf $= 0.5\%$, RF – mean rCu estimates for Left: upper, Right: lower tubes. Showing mean signal and MI-LSD, LSD and LU estimates

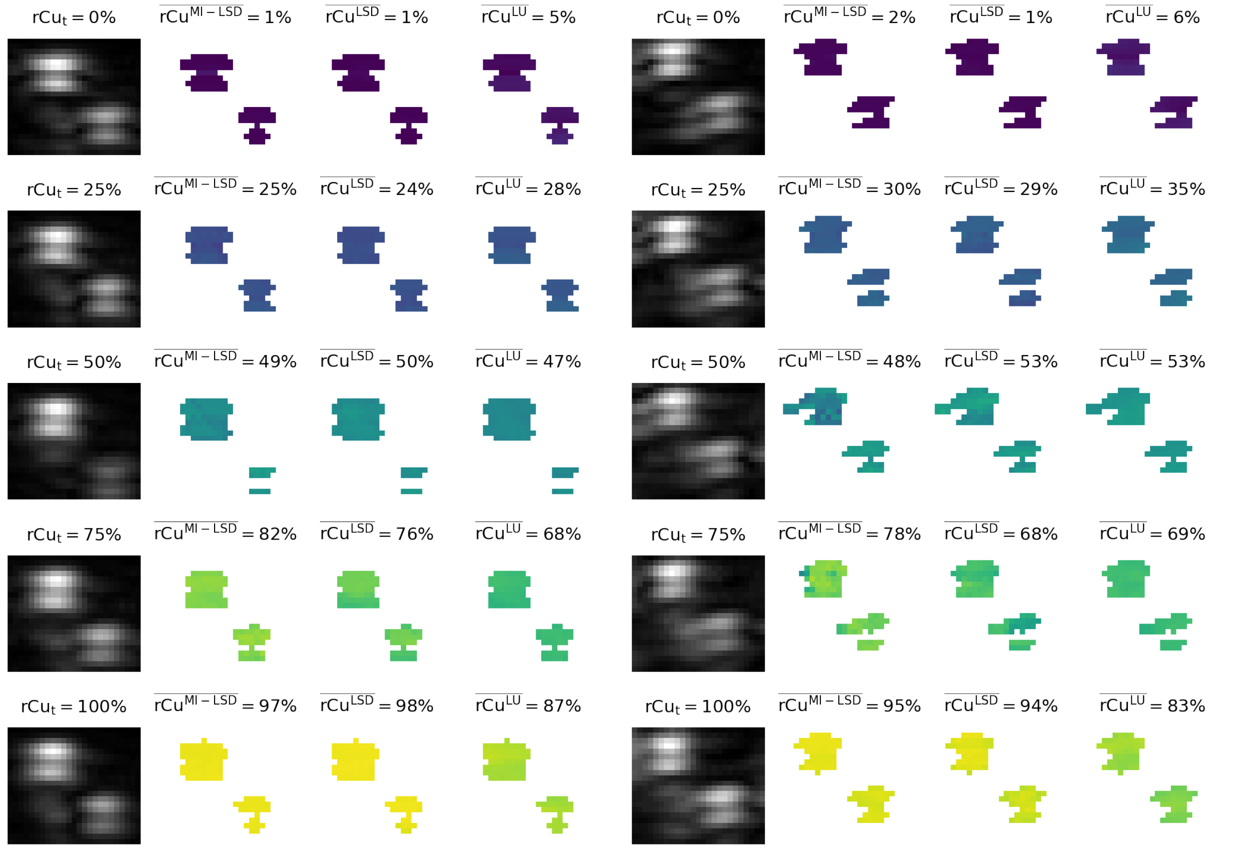

Figure 10: $rCu_{bg} = 75\%$, svf $= 0.5\%$, RF – mean rCu estimates for Left: upper, Right: lower tubes. Showing mean signal and MI-LSD, LSD and LU estimates

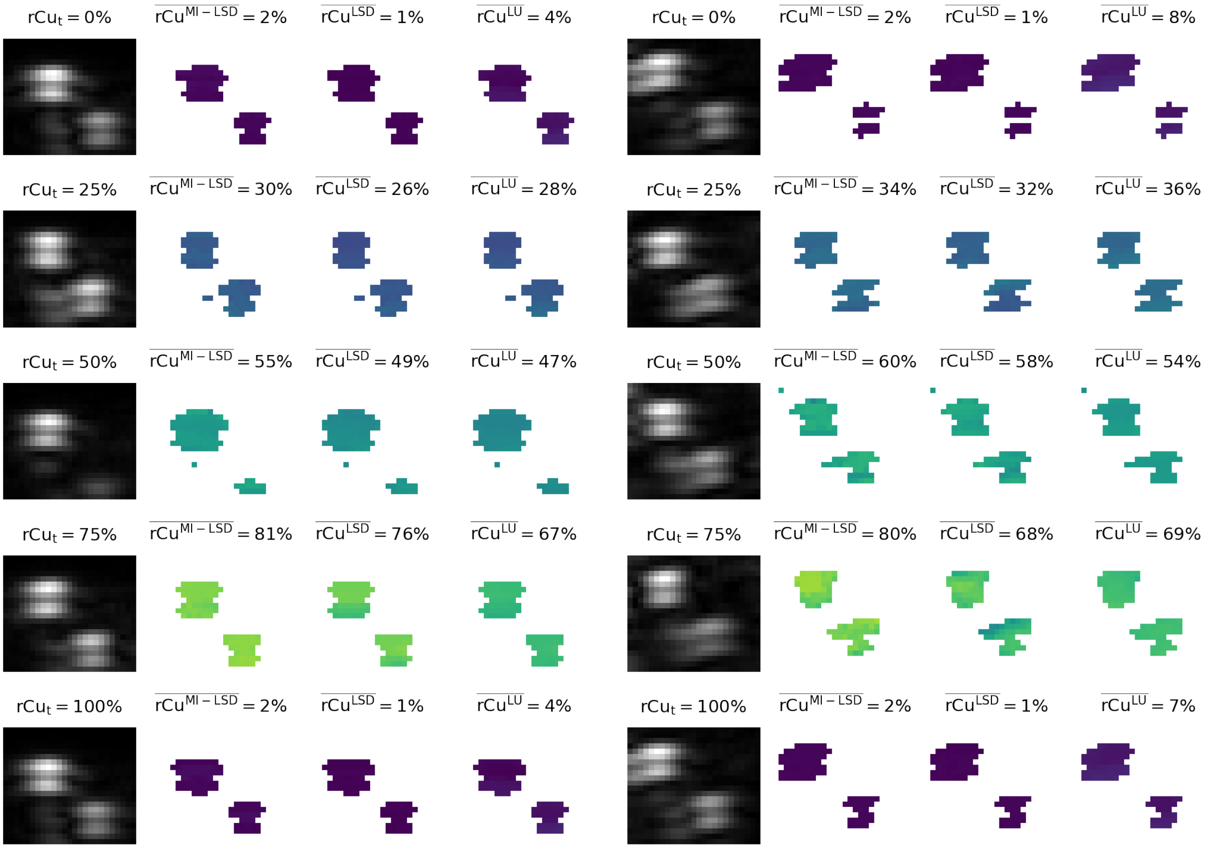

Figure 11: $rCu_{bg} = 50\%$, $svf = 0.5\%$, RF – mean rCu estimates for Left: upper, Right: lower tubes. Showing mean signal and MI-LSD, LSD and LU estimates *Note: Due to a phantom positioning oversight, the* $rCu_t = 100\%$ *measurement had an actual rCu of 0% and was omitted from further analysis.*

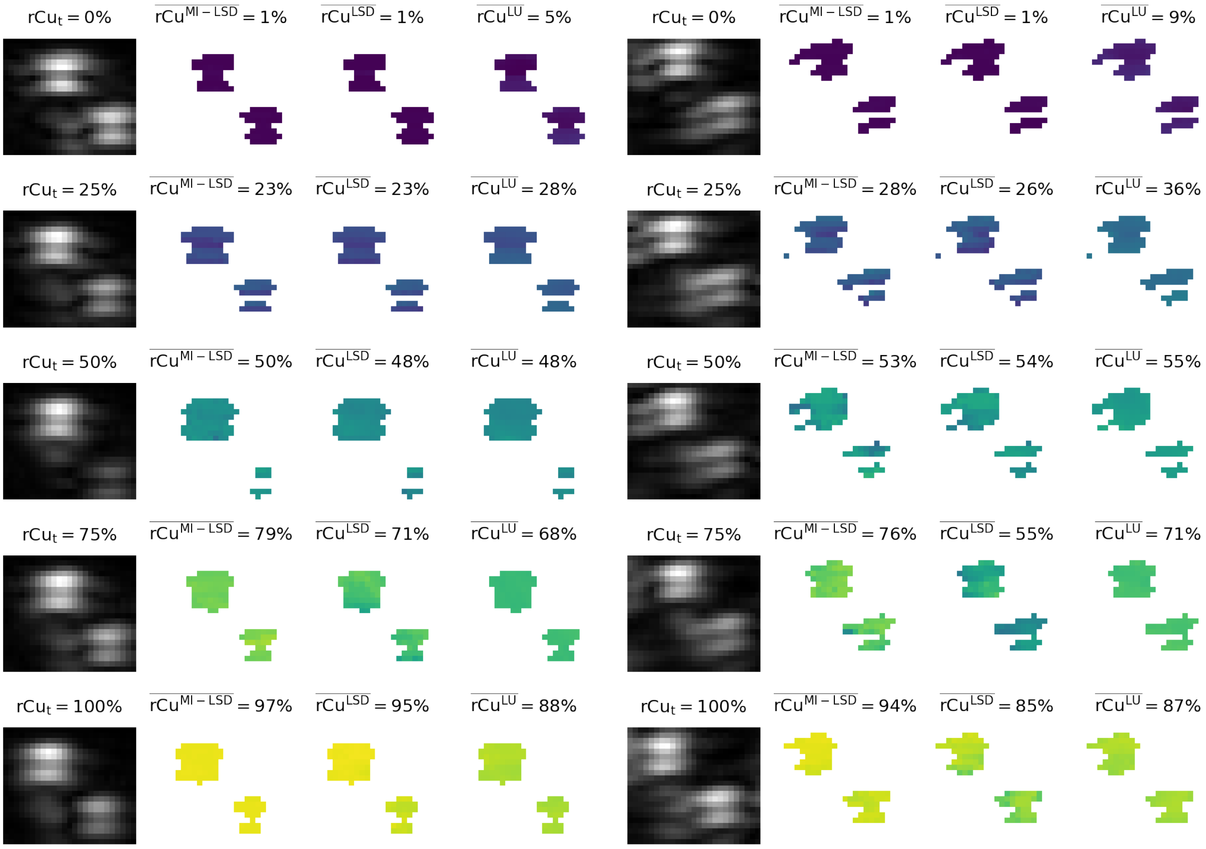

Figure 12: $rCu_{bg} = 25\%$, $svf = 0.5\%$, RF – mean rCu estimates for Left: upper, Right: lower tubes. Showing mean signal and MI-LSD, LSD and LU estimates

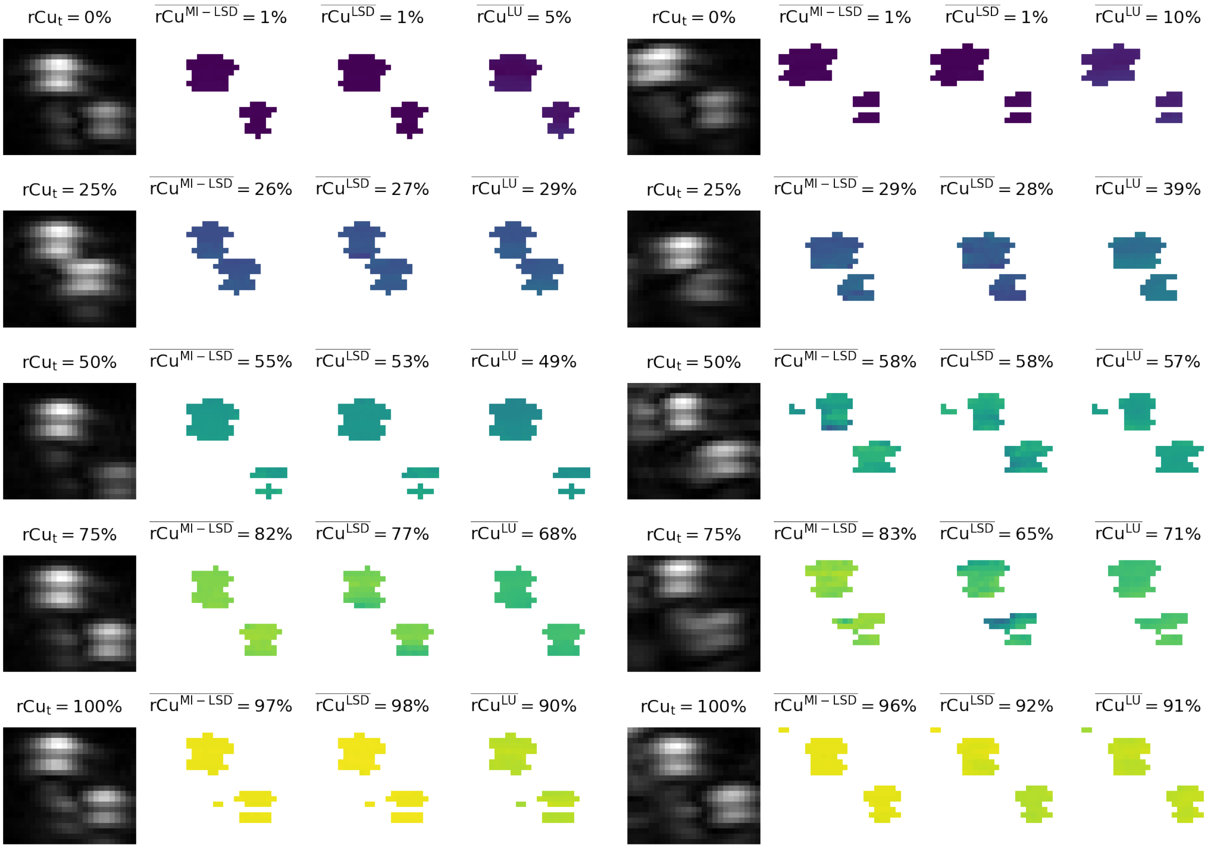

Figure 13: $rCu_{bg} = 0\%$, svf = 0.5%, RF – mean rCu estimates for Left: upper, Right: lower tubes. Showing mean signal and MI-LSD, LSD and LU estimates

2.1.0.3 1.0% sulfate volume fraction (svf)

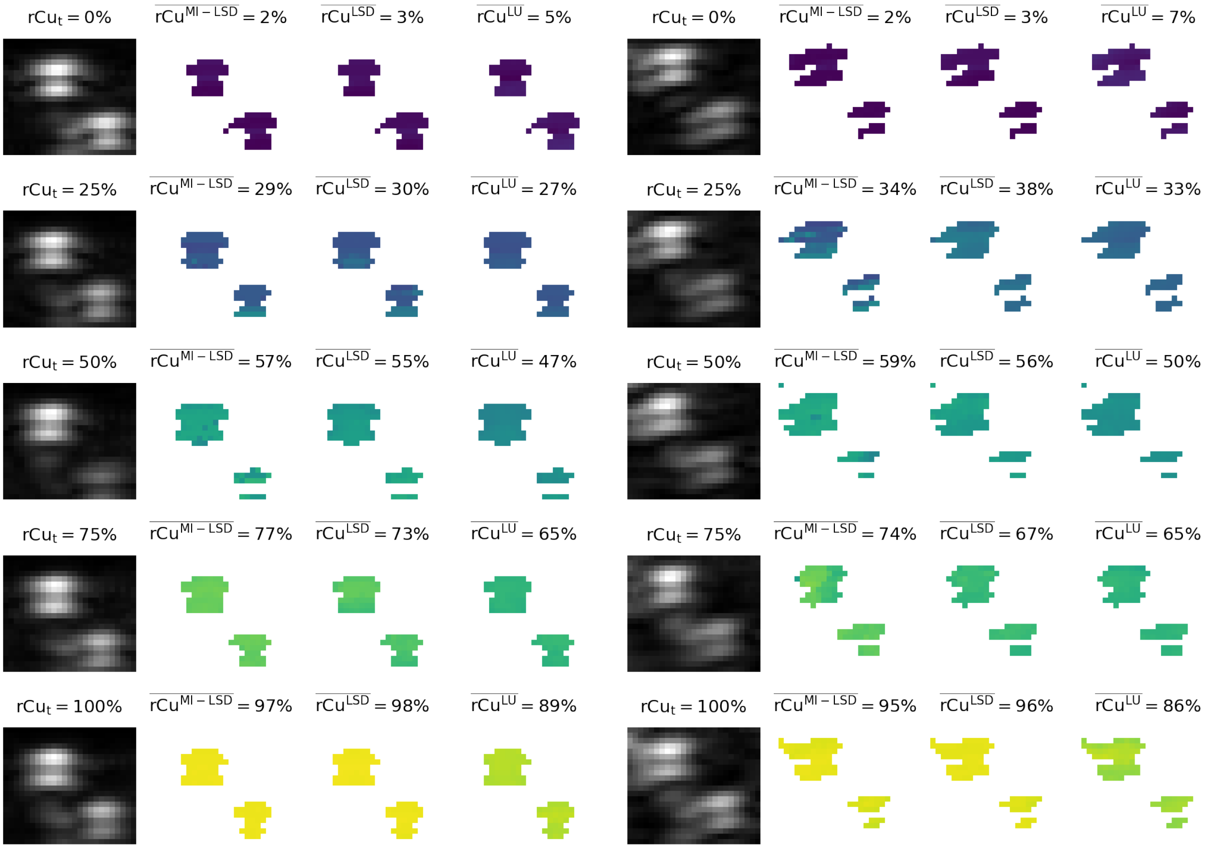

Figure 14: $rCu_{bg} = 100\%$, svf = 1%, RF – mean rCu estimates for Left: upper, Right: lower tubes. Showing mean signal and MI-LSD, LSD and LU estimates

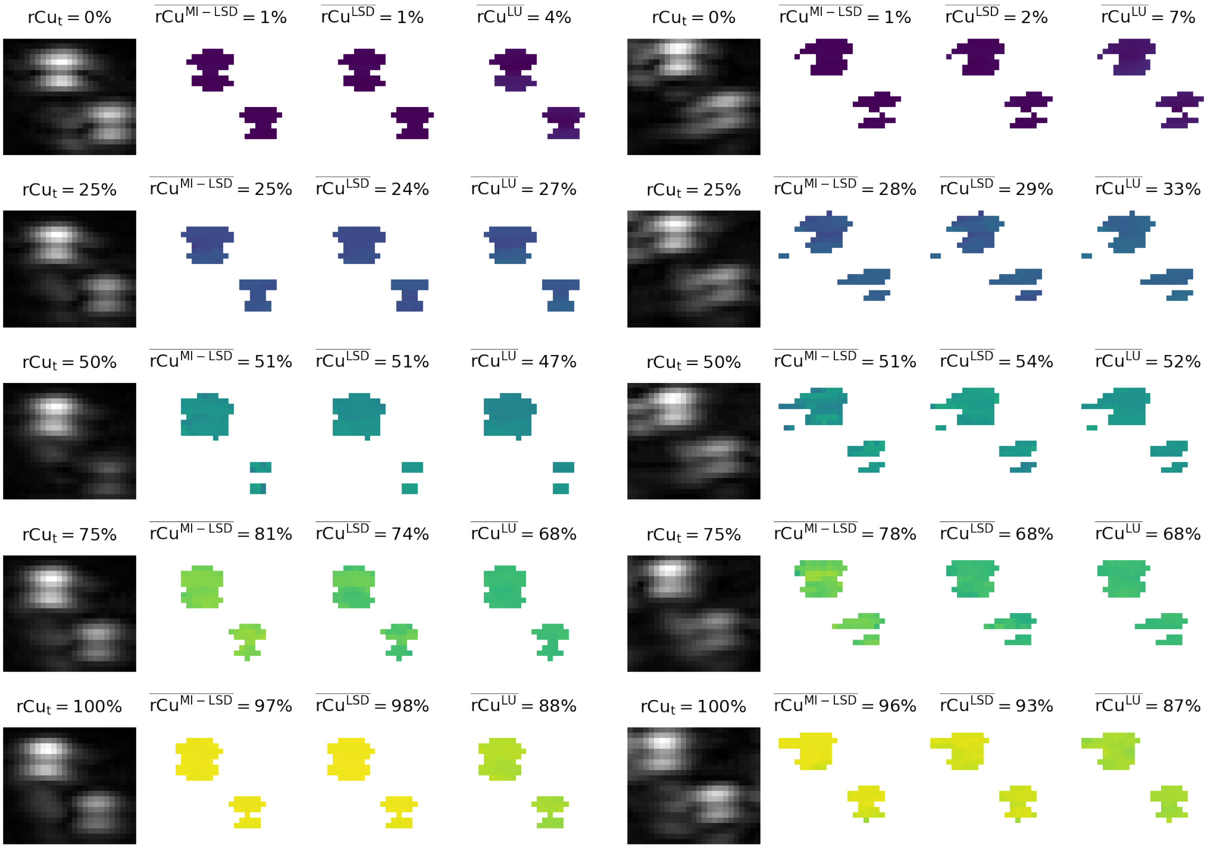

Figure 15: $rCu_{bg} = 75\%$, svf $= 1\%$, RF – mean rCu estimates for Left: upper, Right: lower tubes. Showing mean signal and MI-LSD, LSD and LU estimates

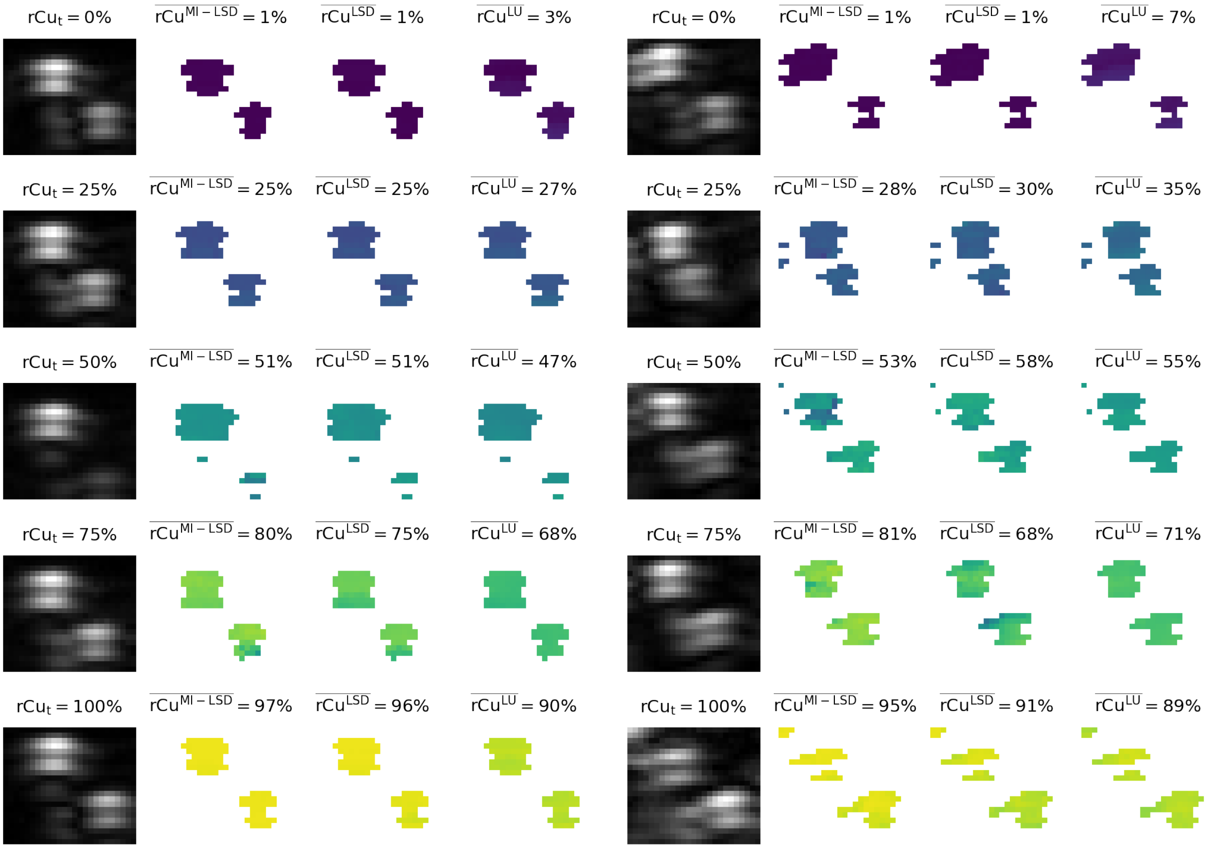

Figure 16: $rCu_{bg} = 50\%$, svf $= 1\%$, RF – mean rCu estimates for Left: upper, Right: lower tubes. Showing mean signal and MI-LSD, LSD and LU estimates

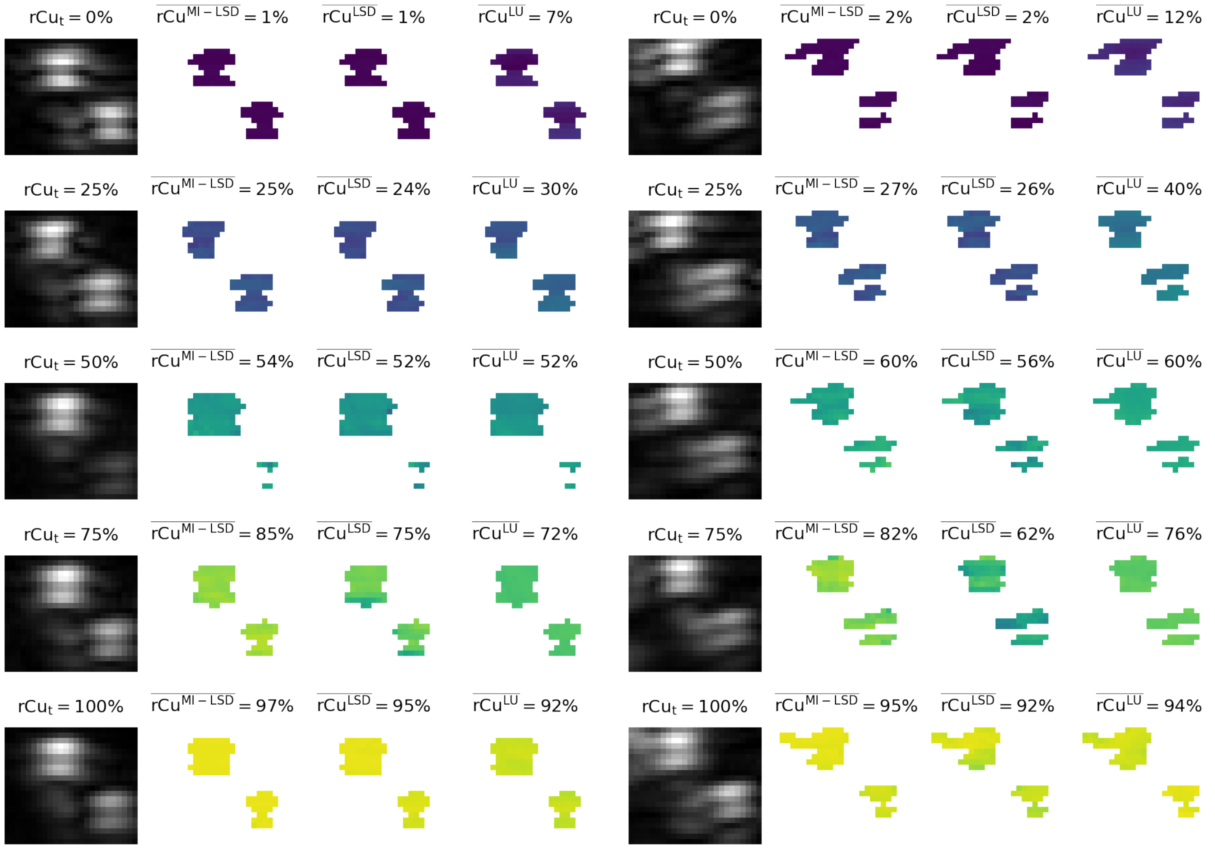

Figure 17: $\mathrm{rCu_{bg}} = 25\%$, svf $= 1\%$, RF – mean rCu estimates for Left: upper, Right: lower tubes. Showing mean signal and MI-LSD, LSD and LU estimates

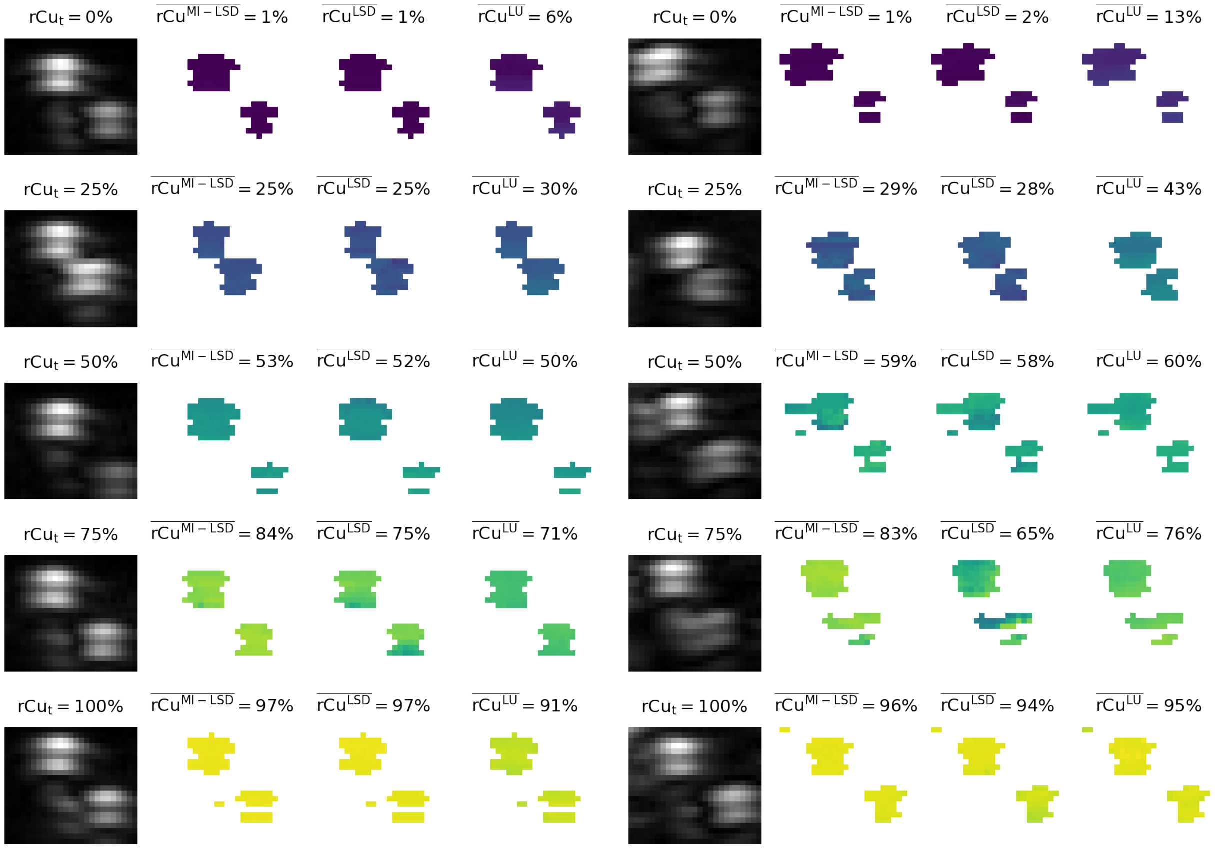

Figure 18: $\mathrm{rCu_{bg}} = 0\%$, svf $= 1\%$, RF – mean rCu estimates for Left: upper, Right: lower tubes. Showing mean signal and MI-LSD, LSD and LU estimates

2.2 Feed Forward Neural Network (NN) – without dropout

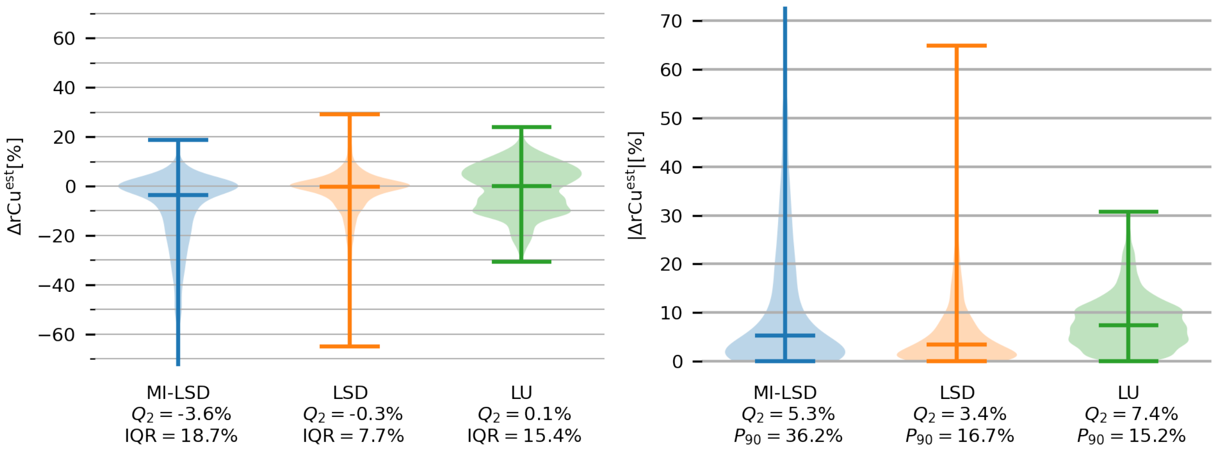

Figure 19: NN (no dropout) error distribution transversal phantom *test* set

2.2.0.1 Baseline – 0% sulfate volume fraction (svf)

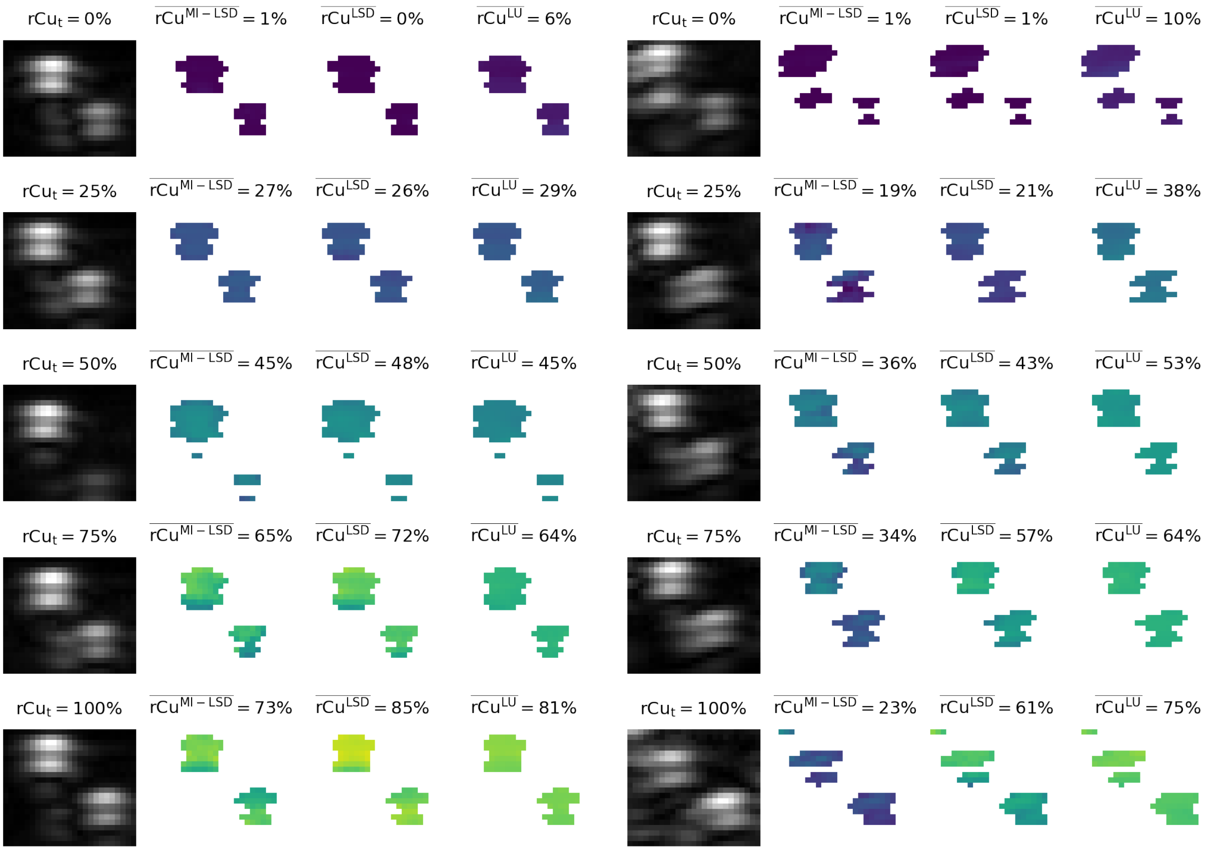

Figure 20: Baseline 0, NN no dropout – mean rCu estimates for Left: upper, Right: lower tubes. Showing mean signal and MI-LSD, LSD and LU estimates

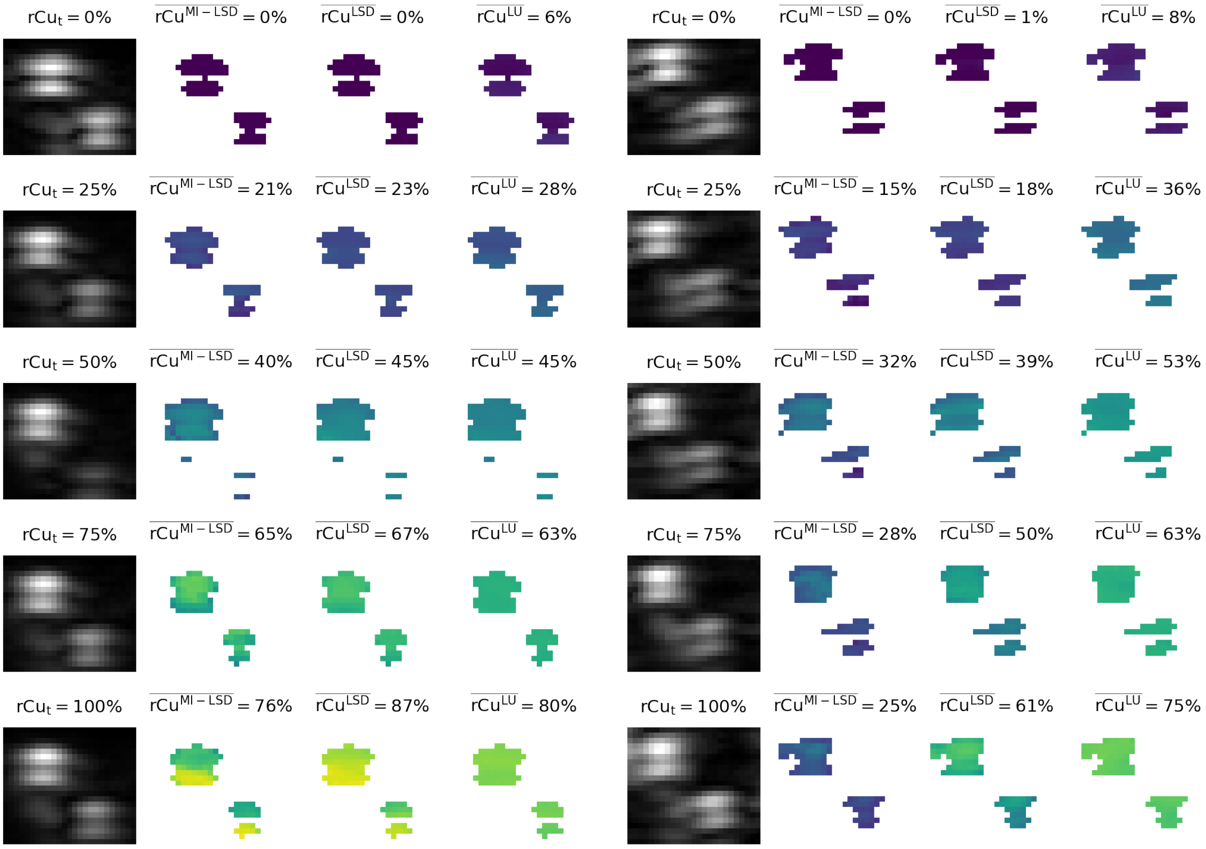

Figure 21: Baseline 1, NN no dropout – mean rCu estimates for Left: upper, Right: lower tubes. Showing mean signal and MI-LSD, LSD and LU estimates

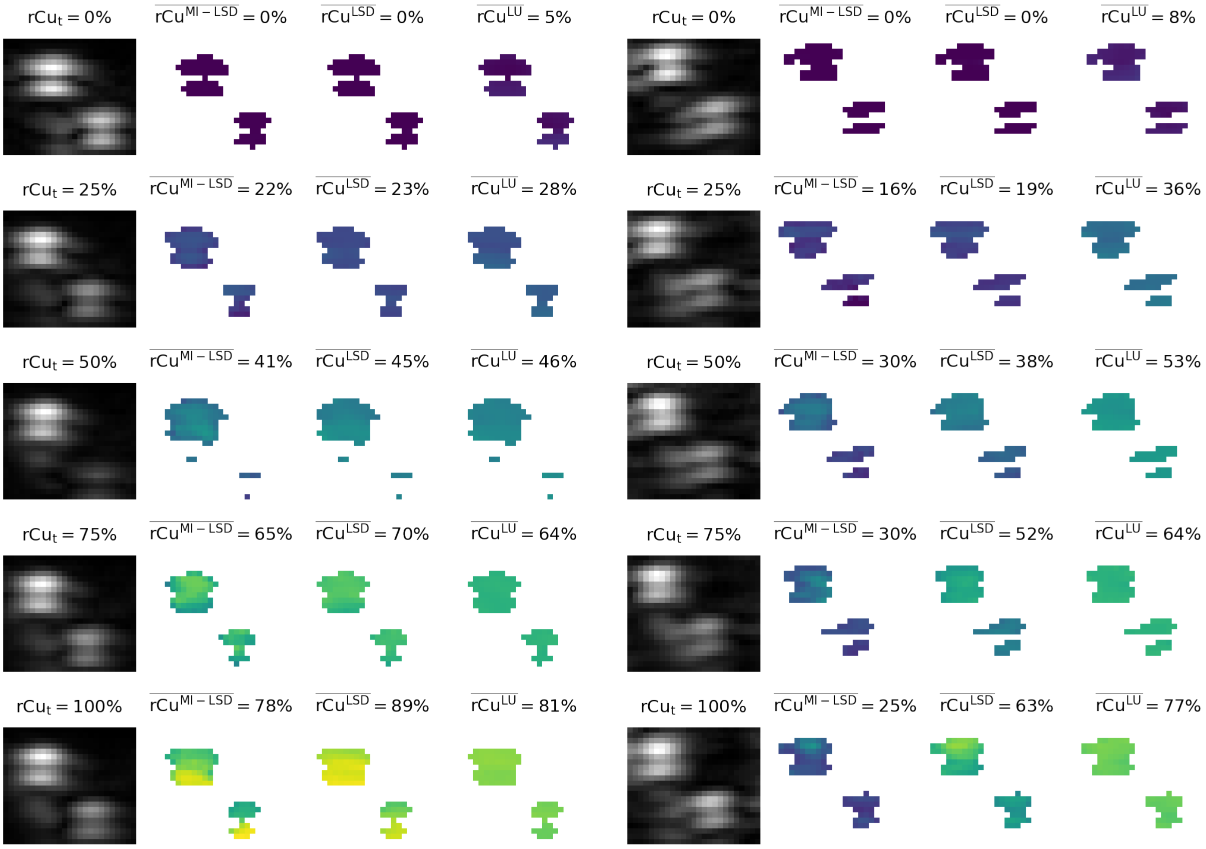

Figure 22: Baseline 2, NN no dropout – mean rCu estimates for Left: upper, Right: lower tubes. Showing mean signal and MI-LSD, LSD and LU estimates

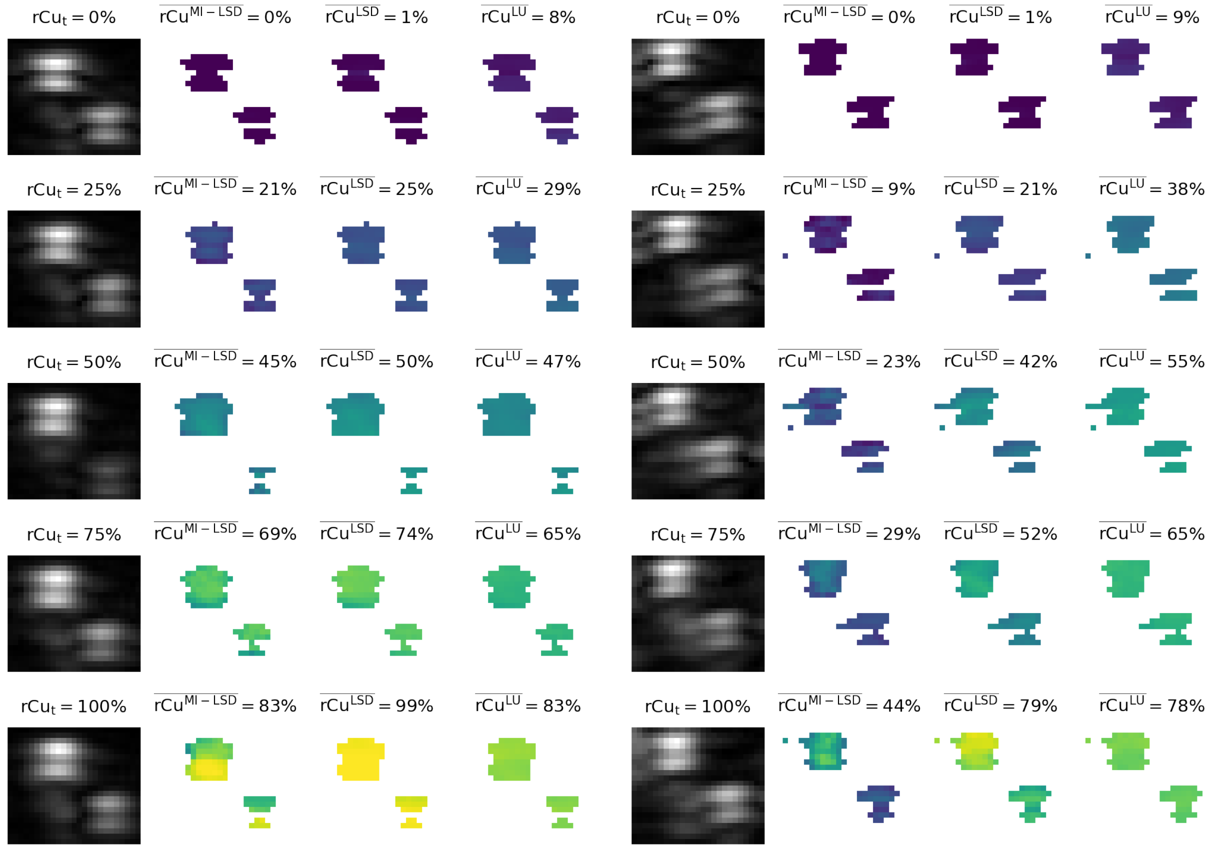

Figure 23: Baseline 3, NN no dropout – mean rCu estimates for Left: upper, Right: lower tubes. Showing mean signal and MI-LSD, LSD and LU estimates

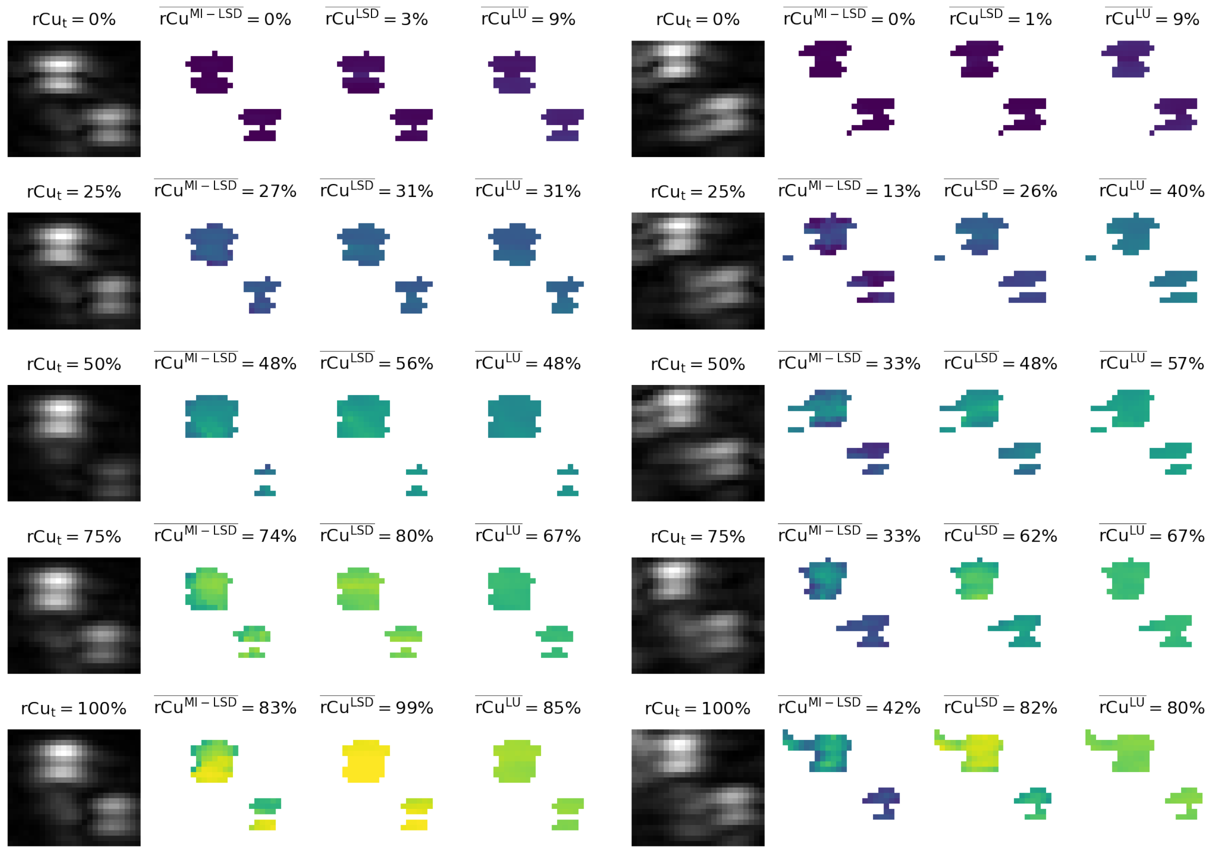

Figure 24: Baseline 4, NN no dropout – mean rCu estimates for Left: upper, Right: lower tubes. Showing mean signal and MI-LSD, LSD and LU estimates

2.2.0.2 0.5% sulfate volume fraction (svf)

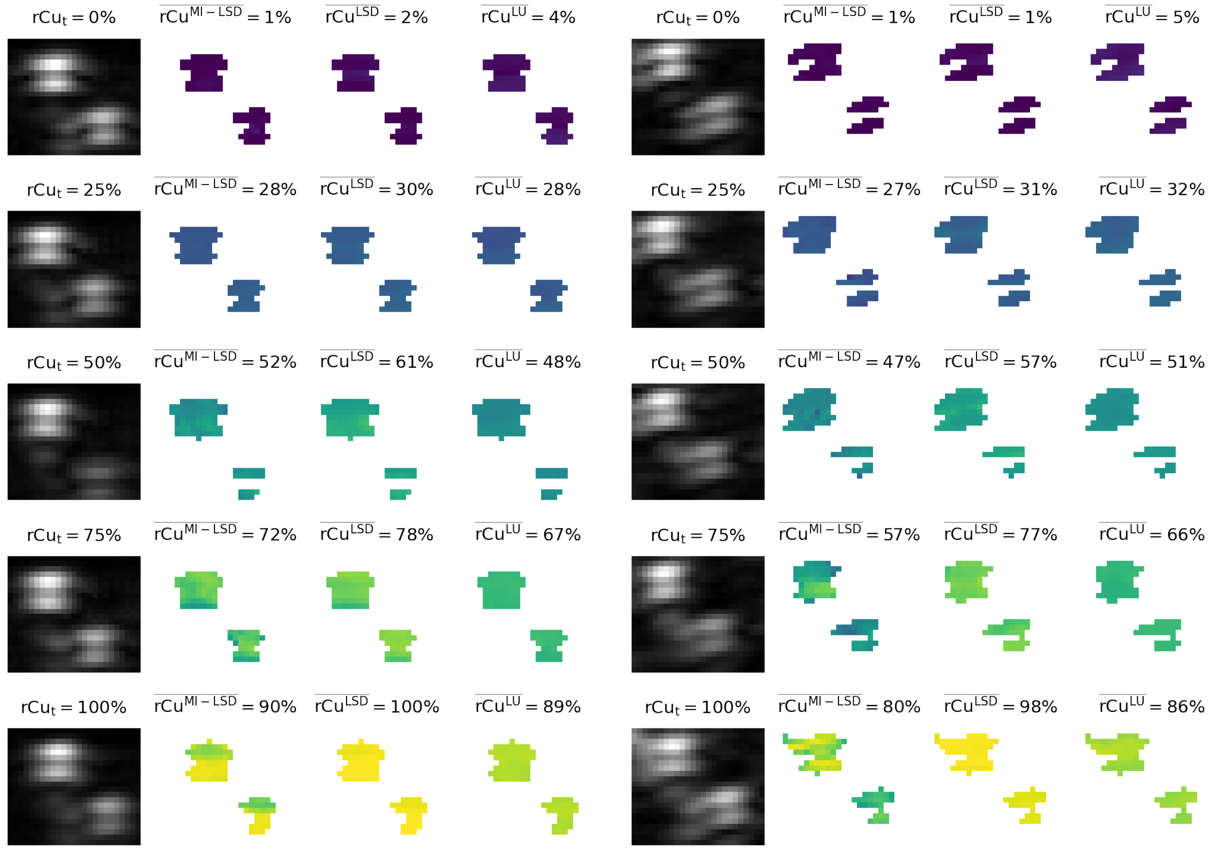

Figure 25: $rCu_{bg} = 100\%$, svf $= 0.5\%$, NN no dropout – mean rCu estimates for Left: upper, Right: lower tubes. Showing mean signal and MI-LSD, LSD and LU estimates

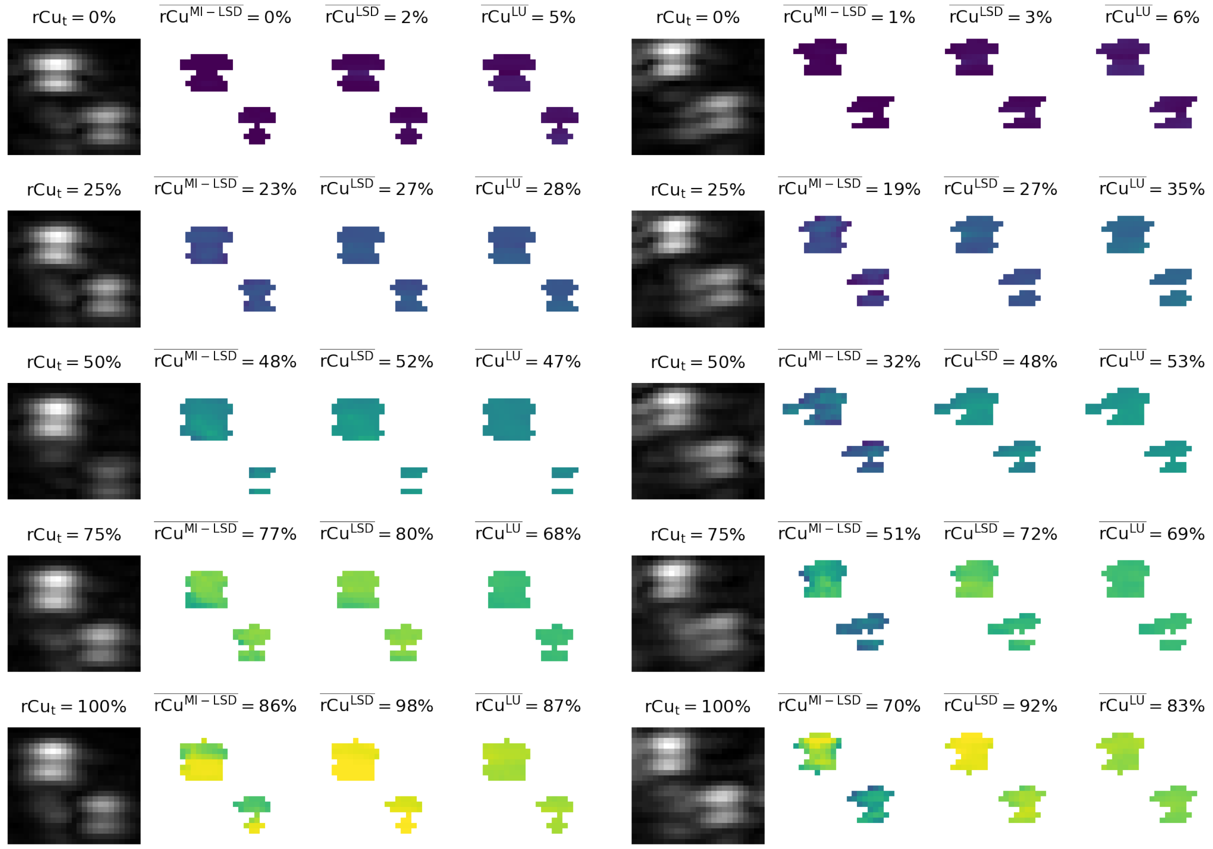

Figure 26: $rCu_{bg} = 75\%$, svf $= 0.5\%$, NN no dropout – mean rCu estimates for Left: upper, Right: lower tubes. Showing mean signal and MI-LSD, LSD and LU estimates

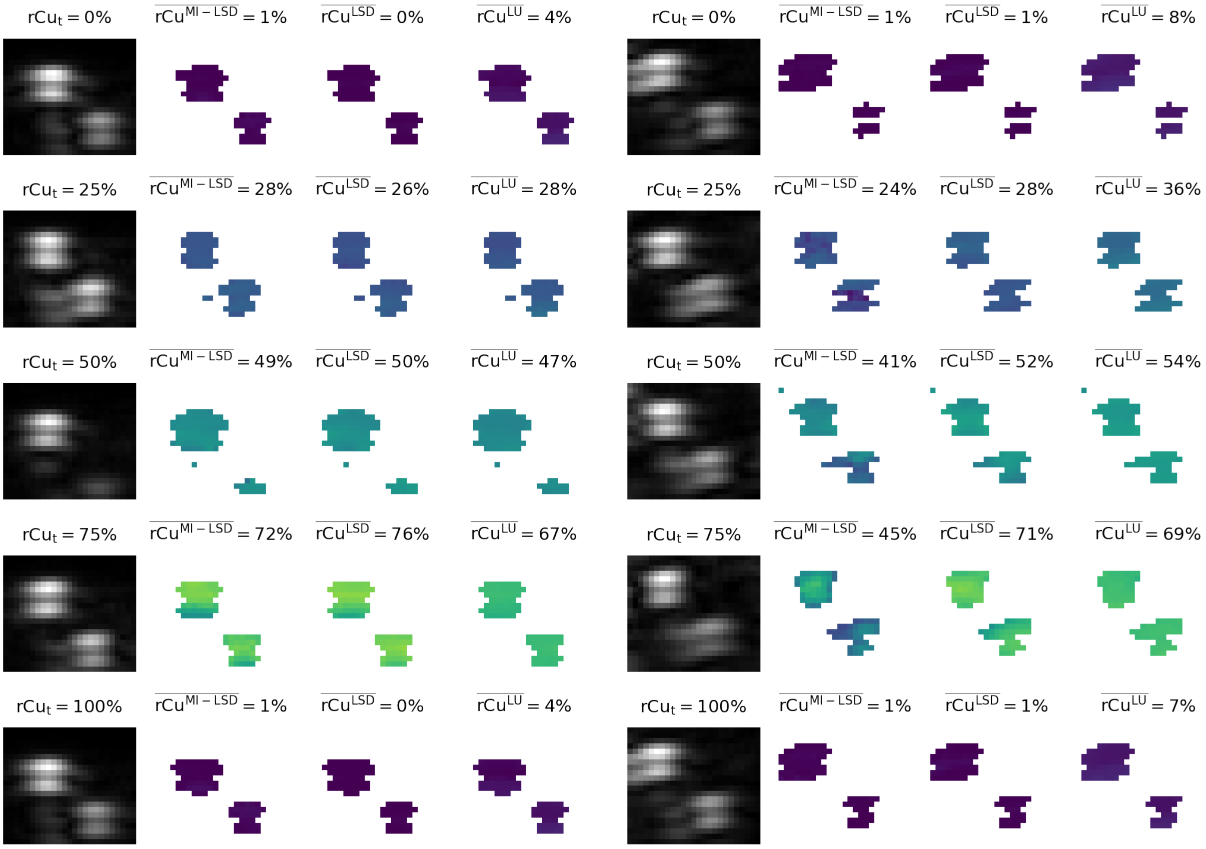

Figure 27: $\text{rCu}_{\text{bg}} = 50\%$, svf $= 0.5\%$, NN no dropout – mean rCu estimates for Left: upper, Right: lower tubes. Showing mean signal and MI-LSD, LSD and LU estimates *Note: Due to a phantom positioning oversight, the* $\text{rCu}_\text{t} = 100\%$ *measurement had an actual rCu of 0% and was omitted from further analysis.*

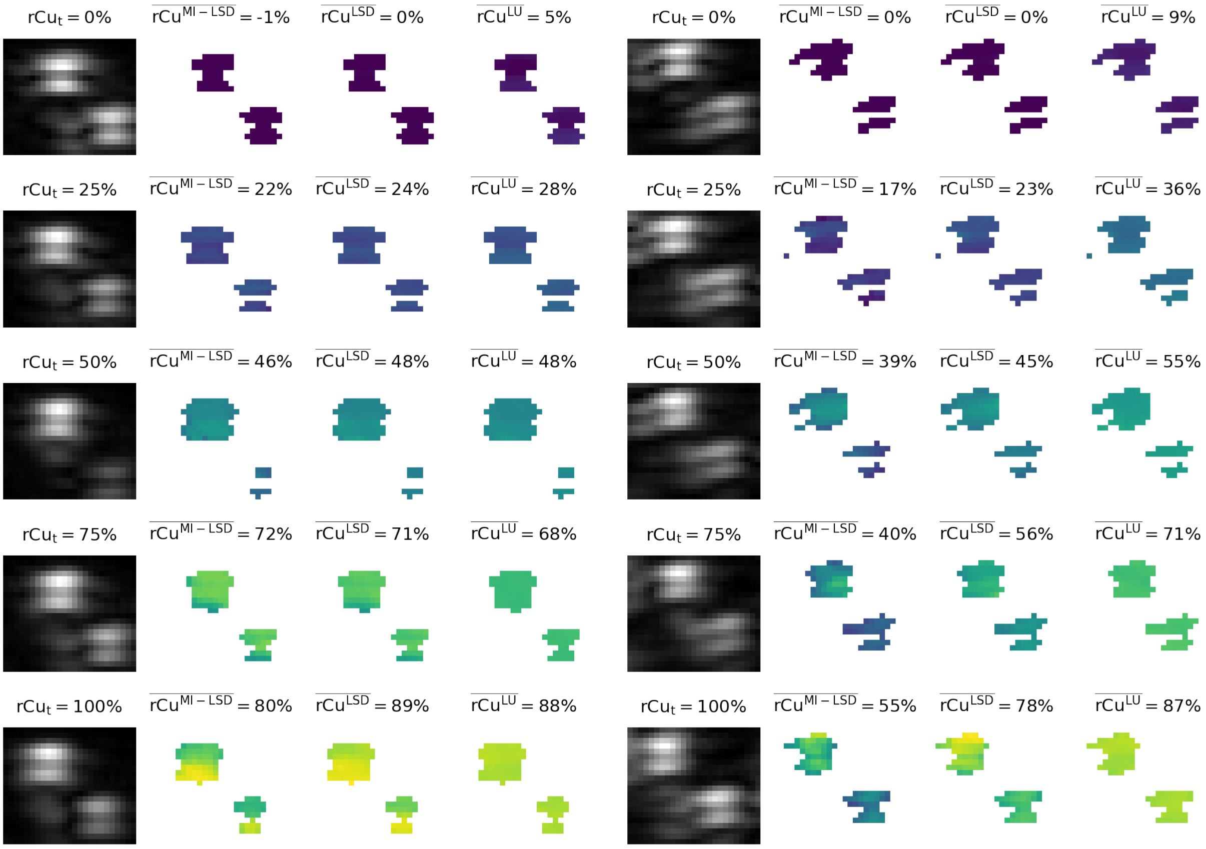

Figure 28: $\text{rCu}_{\text{bg}} = 25\%$, svf $= 0.5\%$, NN no dropout – mean rCu estimates for Left: upper, Right: lower tubes. Showing mean signal and MI-LSD, LSD and LU estimates

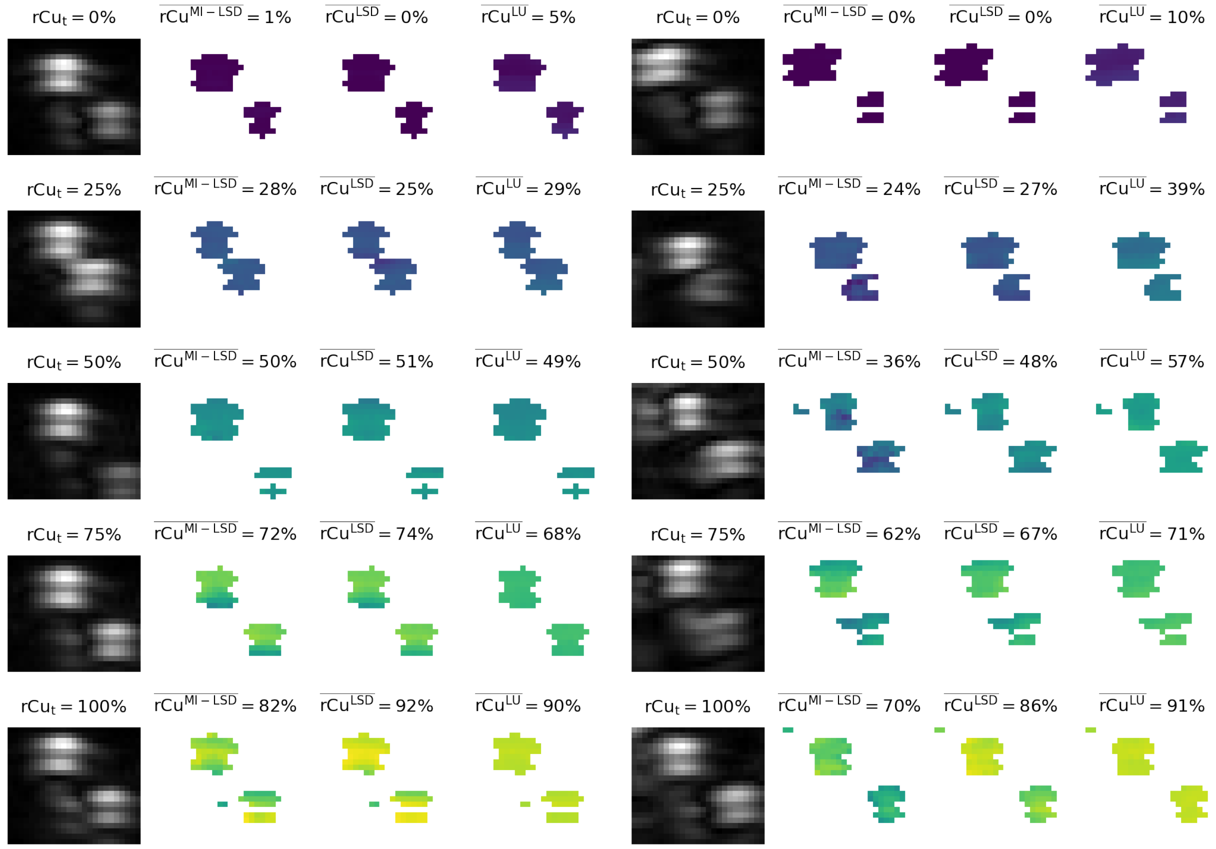

Figure 29: $rCu_{bg} = 0\%$, svf $= 0.5\%$, NN no dropout – mean rCu estimates for Left: upper, Right: lower tubes. Showing mean signal and MI-LSD, LSD and LU estimates

2.2.0.3 1.0% sulfate volume fraction (svf)

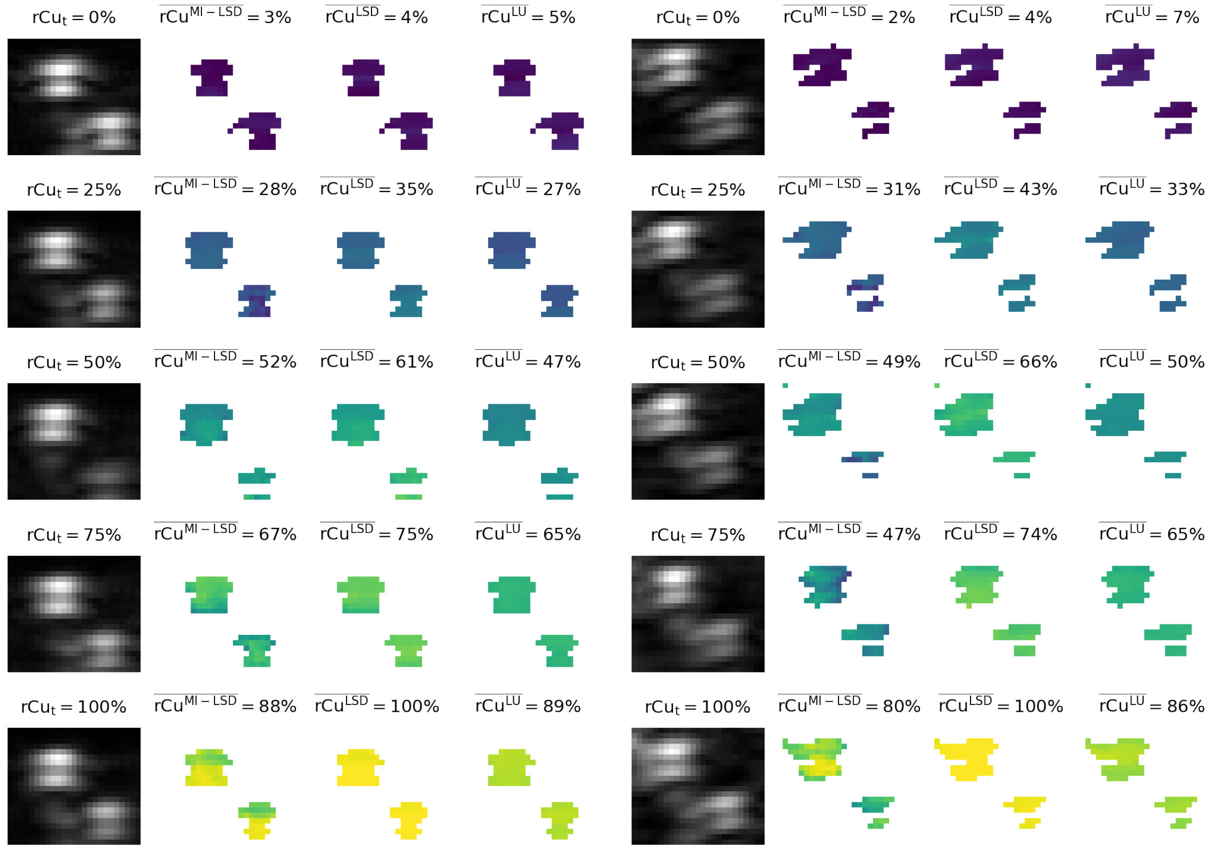

Figure 30: $rCu_{bg} = 100\%$, svf $= 1\%$, NN no dropout – mean rCu estimates for Left: upper, Right: lower tubes. Showing mean signal and MI-LSD, LSD and LU estimates

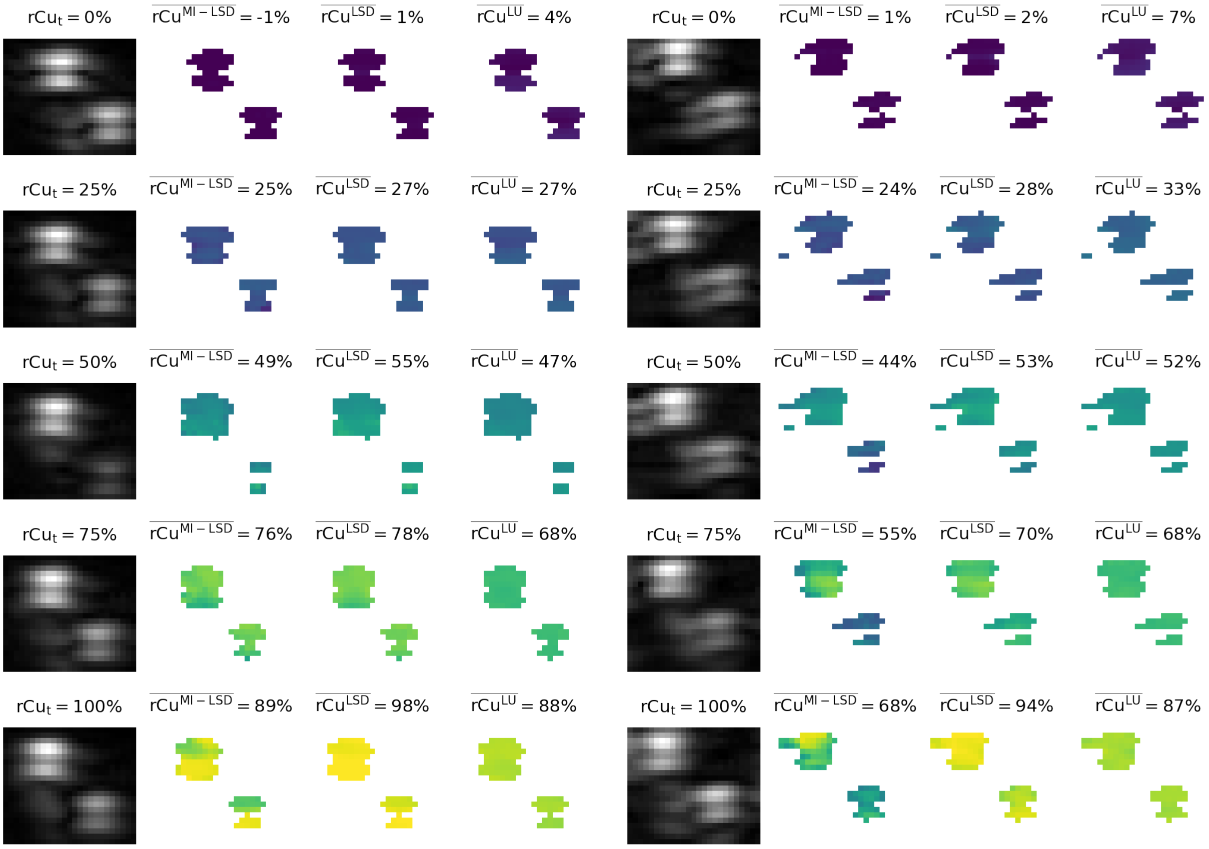

Figure 31: $rCu_{bg} = 75\%$, svf $= 1\%$, NN no dropout – mean rCu estimates for Left: upper, Right: lower tubes. Showing mean signal and MI-LSD, LSD and LU estimates

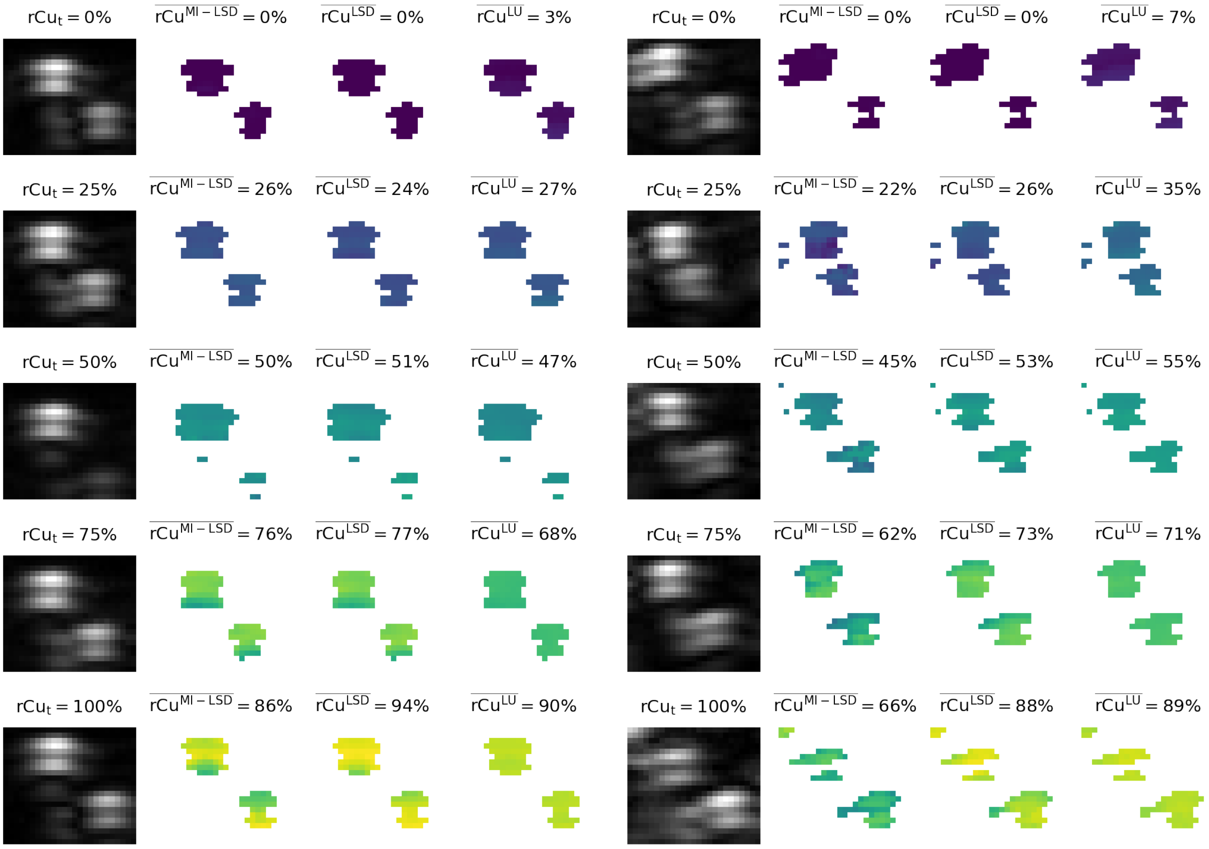

Figure 32: $rCu_{bg} = 50\%$, svf $= 1\%$, NN no dropout – mean rCu estimates for Left: upper, Right: lower tubes. Showing mean signal and MI-LSD, LSD and LU estimates

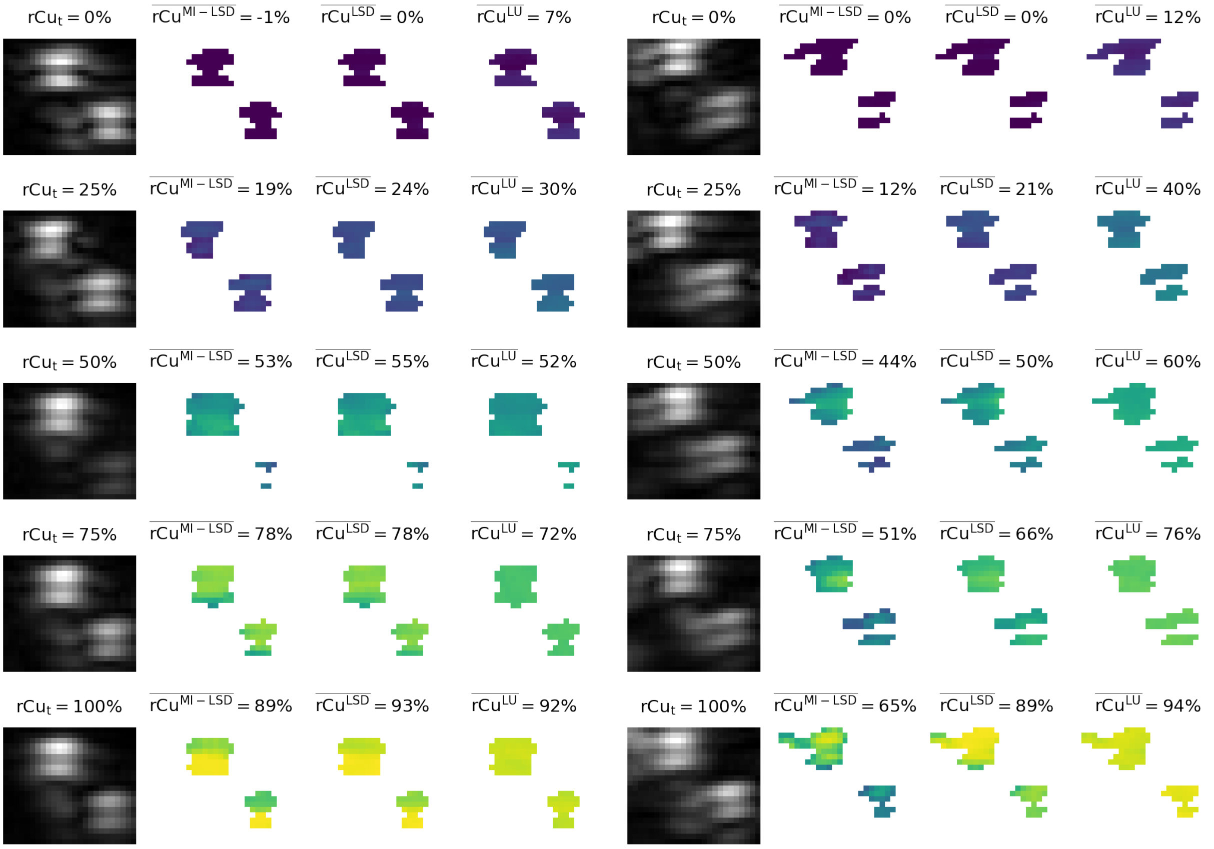

Figure 33: $\mathrm{rCu_{bg}} = 25\%$, svf $= 1\%$, NN no dropout – mean rCu estimates for Left: upper, Right: lower tubes. Showing mean signal and MI-LSD, LSD and LU estimates

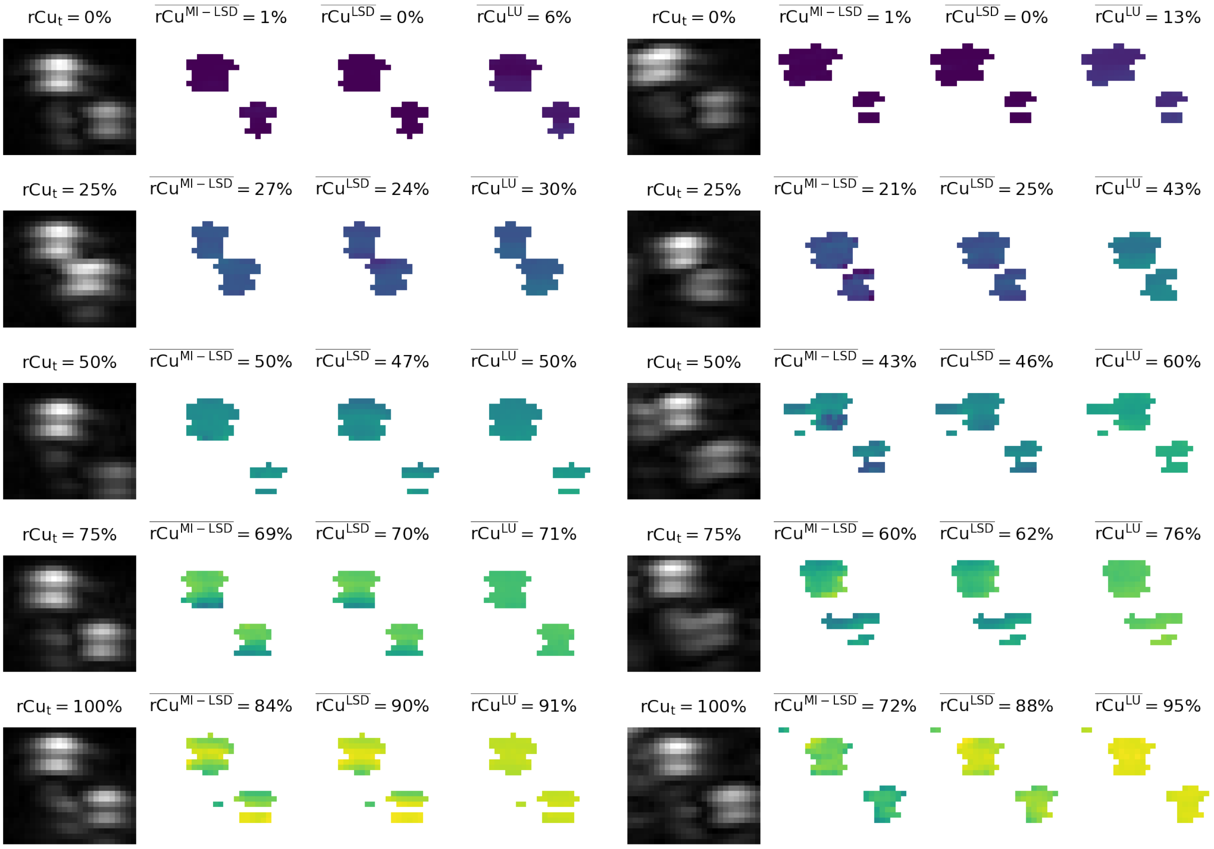

Figure 34: $\mathrm{rCu_{bg}} = 0\%$, svf $= 1\%$, NN no dropout – mean rCu estimates for Left: upper, Right: lower tubes. Showing mean signal and MI-LSD, LSD and LU estimates

2.3 Feed Forward Neural Network (NN) – with dropout

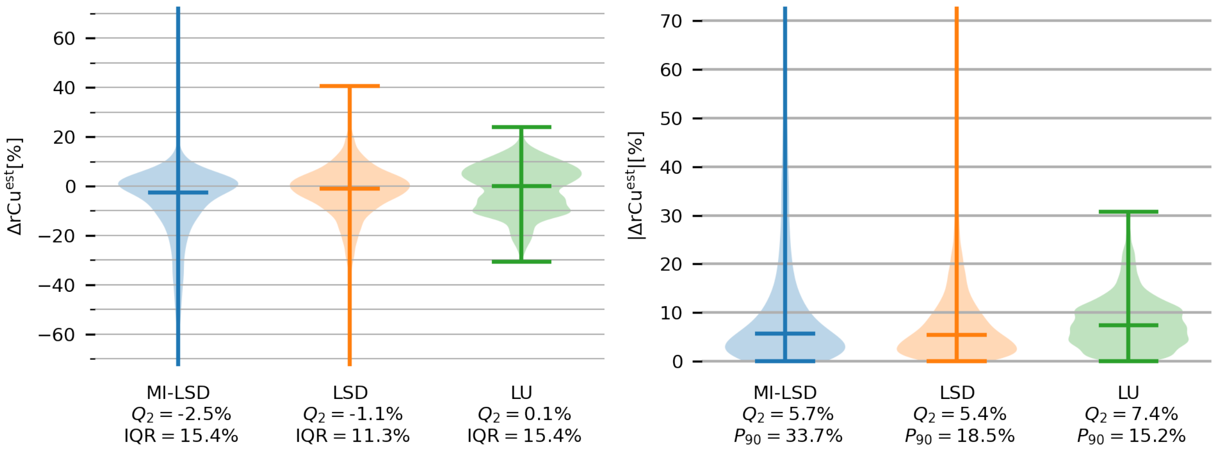

Figure 35: NN (with dropout) error distribution transversal phantom *test* set

2.3.0.1 Baseline – 0% sulfate volume fraction (svf)

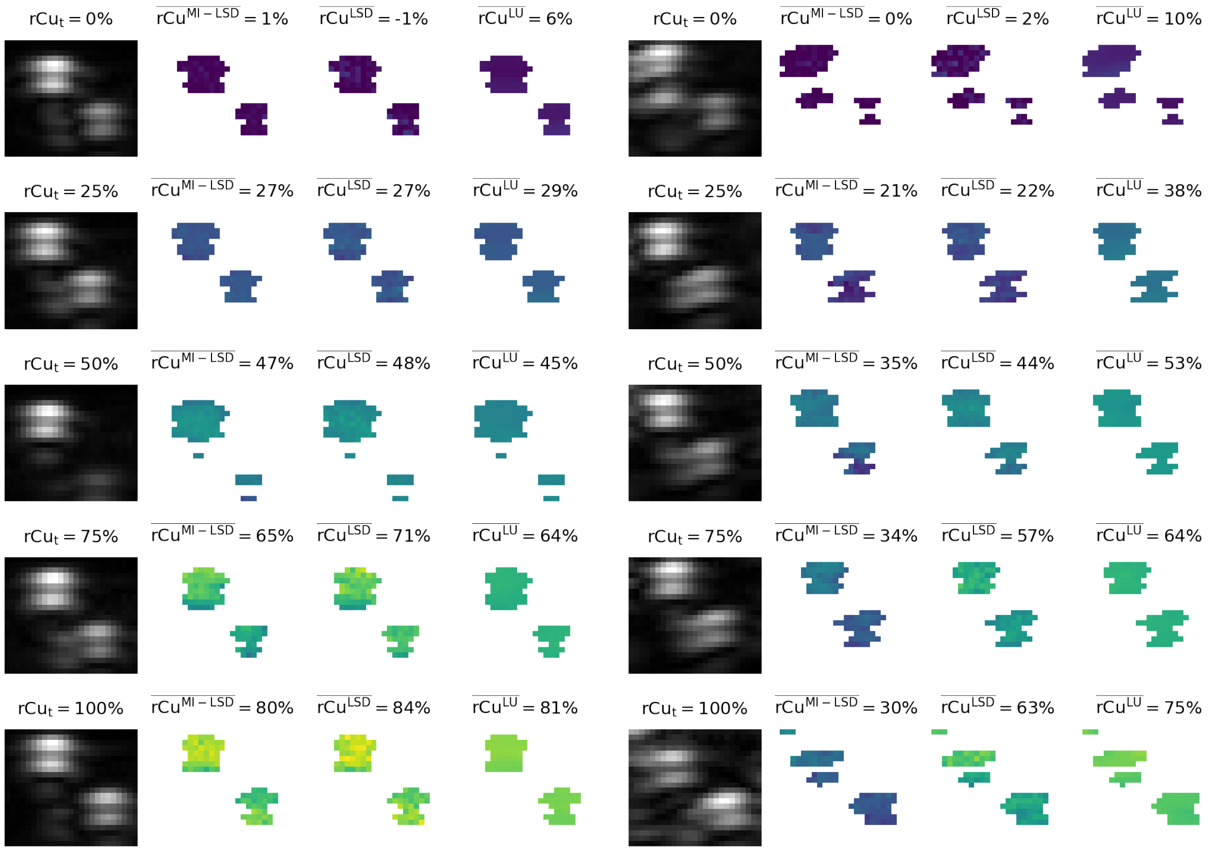

Figure 36: Baseline 0, NN with dropout – mean rCu estimates for Left: upper, Right: lower tubes. Showing mean signal and MI-LSD, LSD and LU estimates

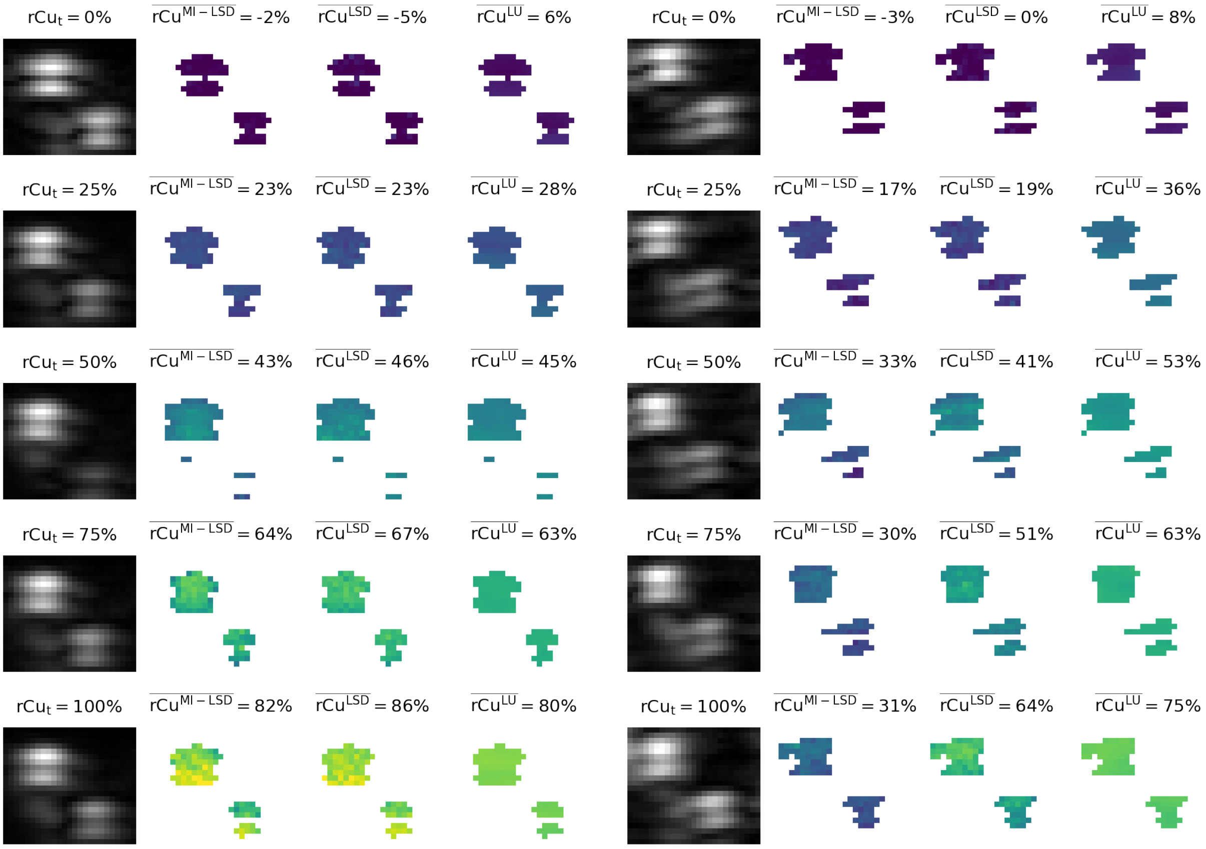

Figure 37: Baseline 1, NN with dropout – mean rCu estimates for Left: upper, Right: lower tubes. Showing mean signal and MI-LSD, LSD and LU estimates

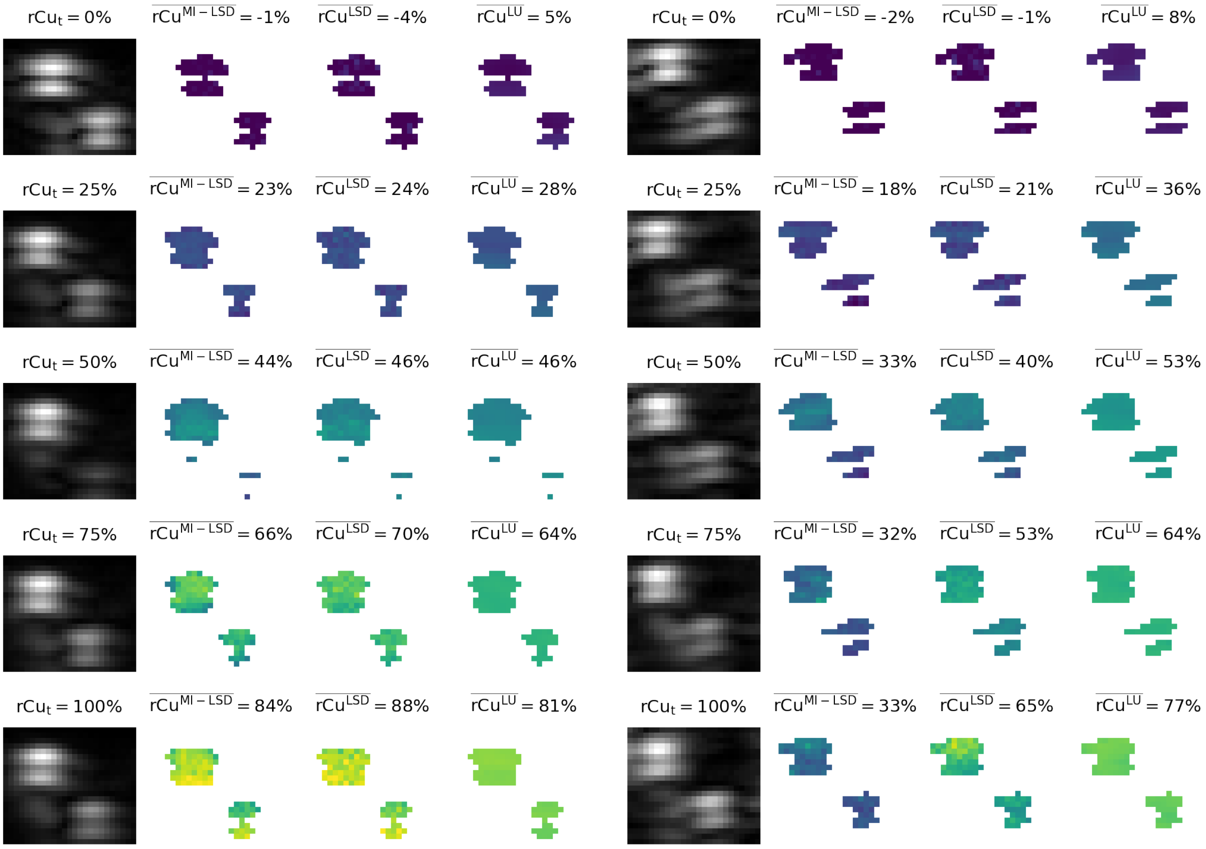

Figure 38: Baseline 2, NN with dropout – mean rCu estimates for Left: upper, Right: lower tubes. Showing mean signal and MI-LSD, LSD and LU estimates

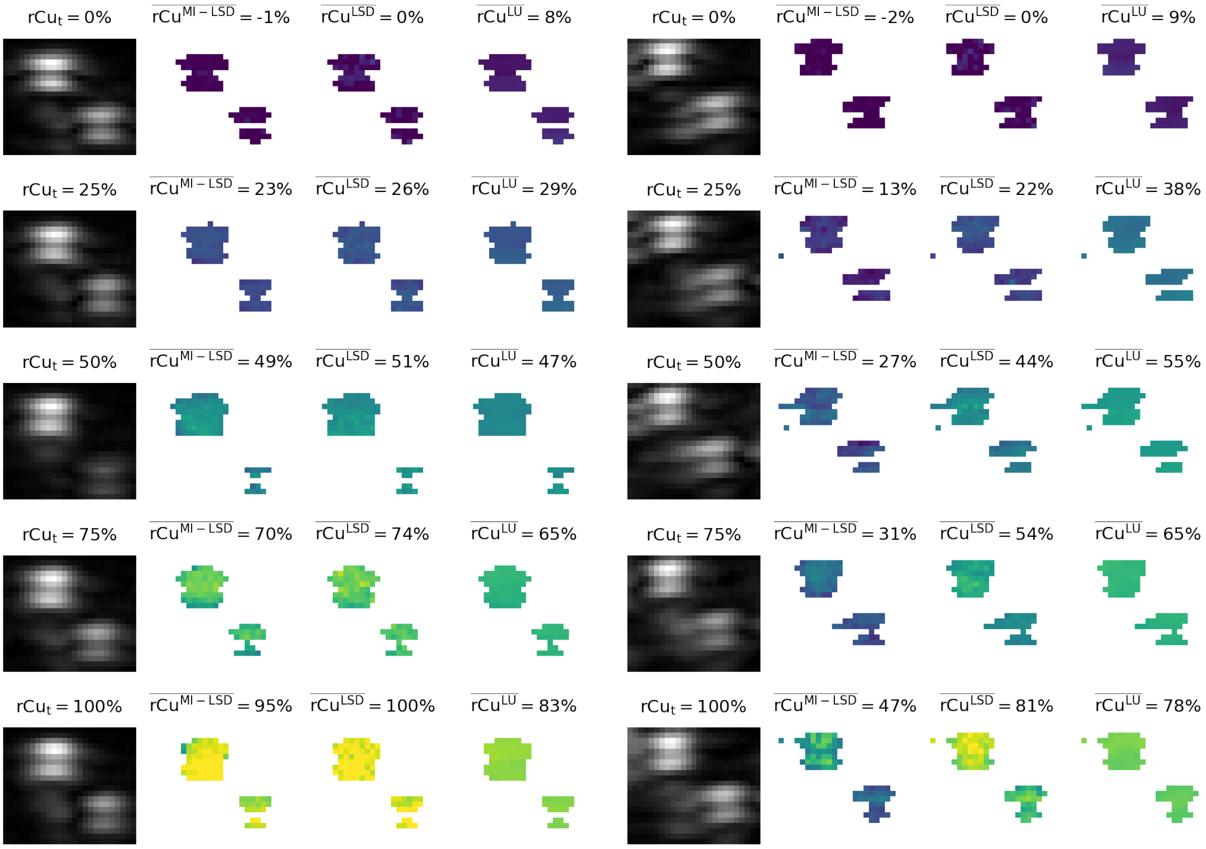

Figure 39: Baseline 3, NN with dropout – mean rCu estimates for Left: upper, Right: lower tubes. Showing mean signal and MI-LSD, LSD and LU estimates

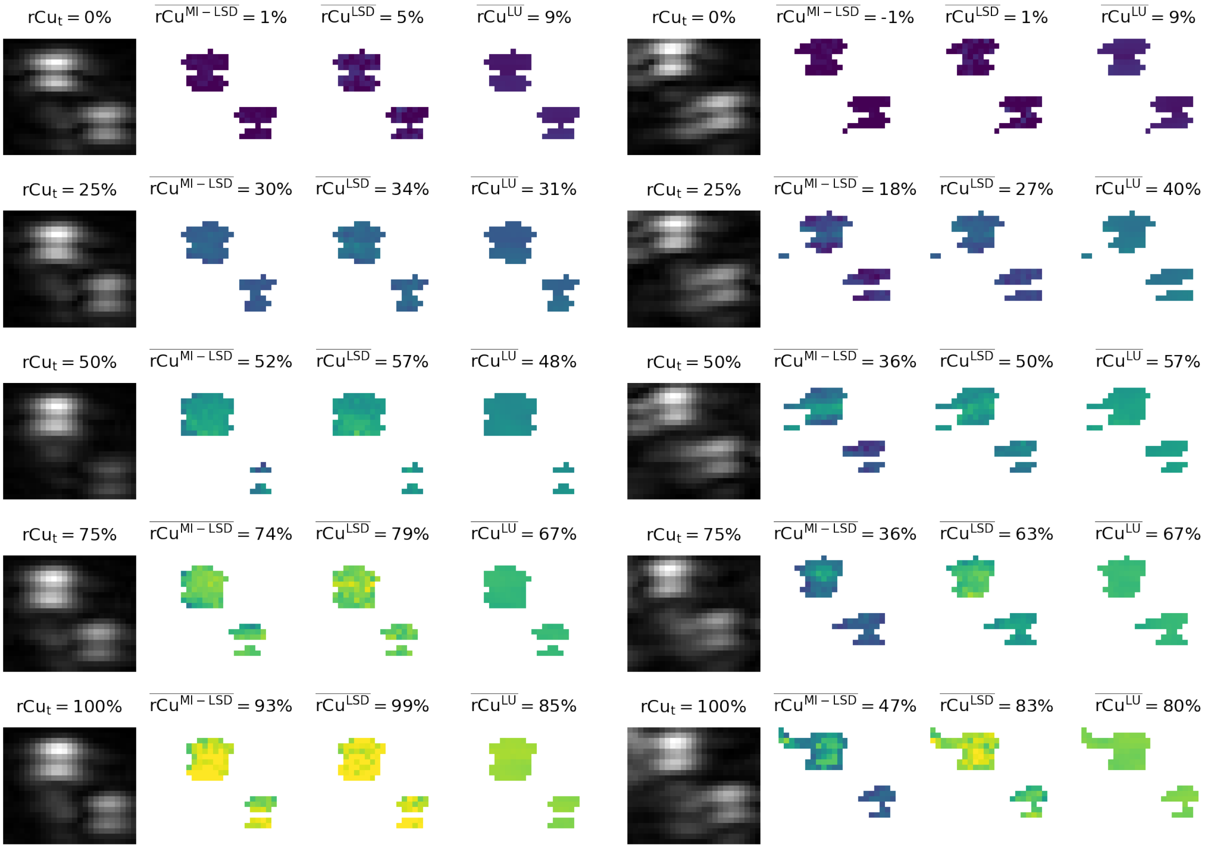

Figure 40: Baseline 4, NN with dropout – mean rCu estimates for Left: upper, Right: lower tubes. Showing mean signal and MI-LSD, LSD and LU estimates

2.3.0.2 0.5% sulfate volume fraction (svf)

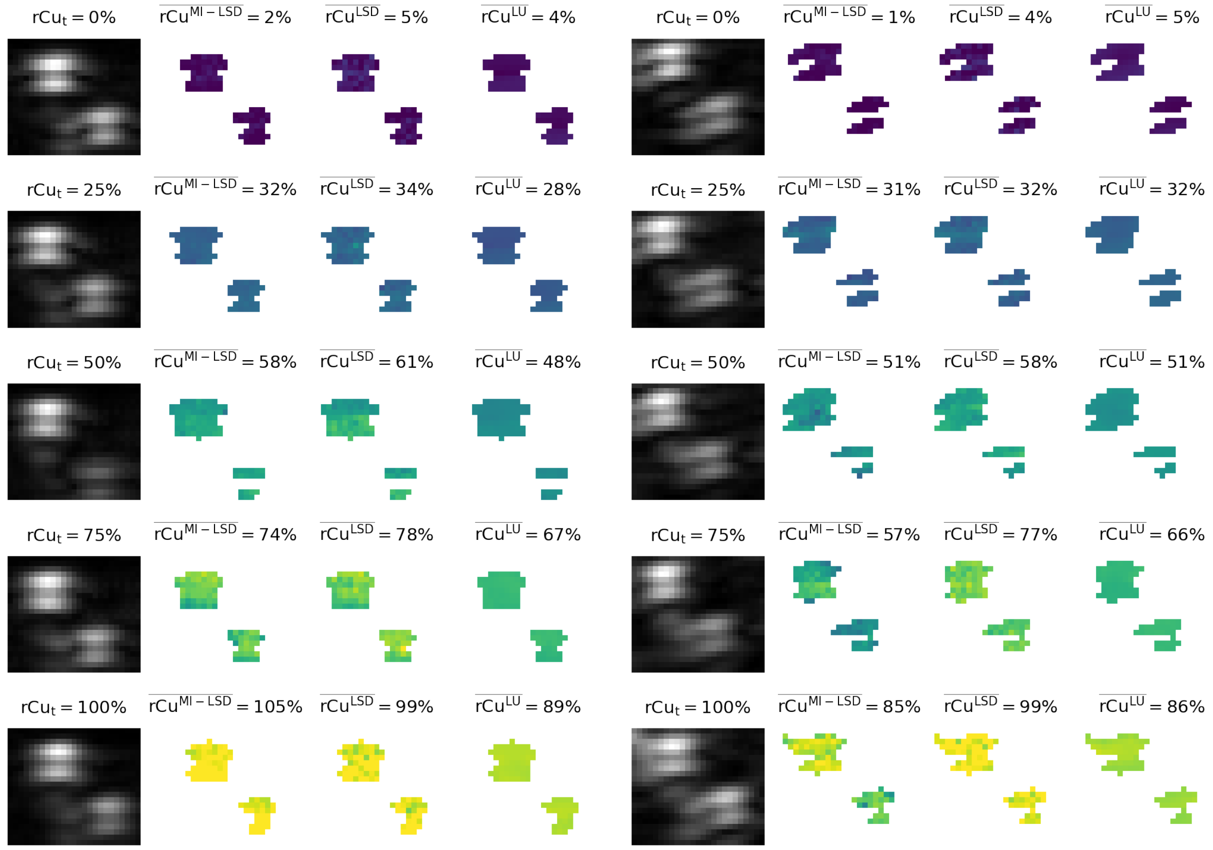

Figure 41: $rCu_{bg} = 100\%$, svf = 0.5%, NN with dropout – mean rCu estimates for Left: upper, Right: lower tubes. Showing mean signal and MI-LSD, LSD and LU estimates

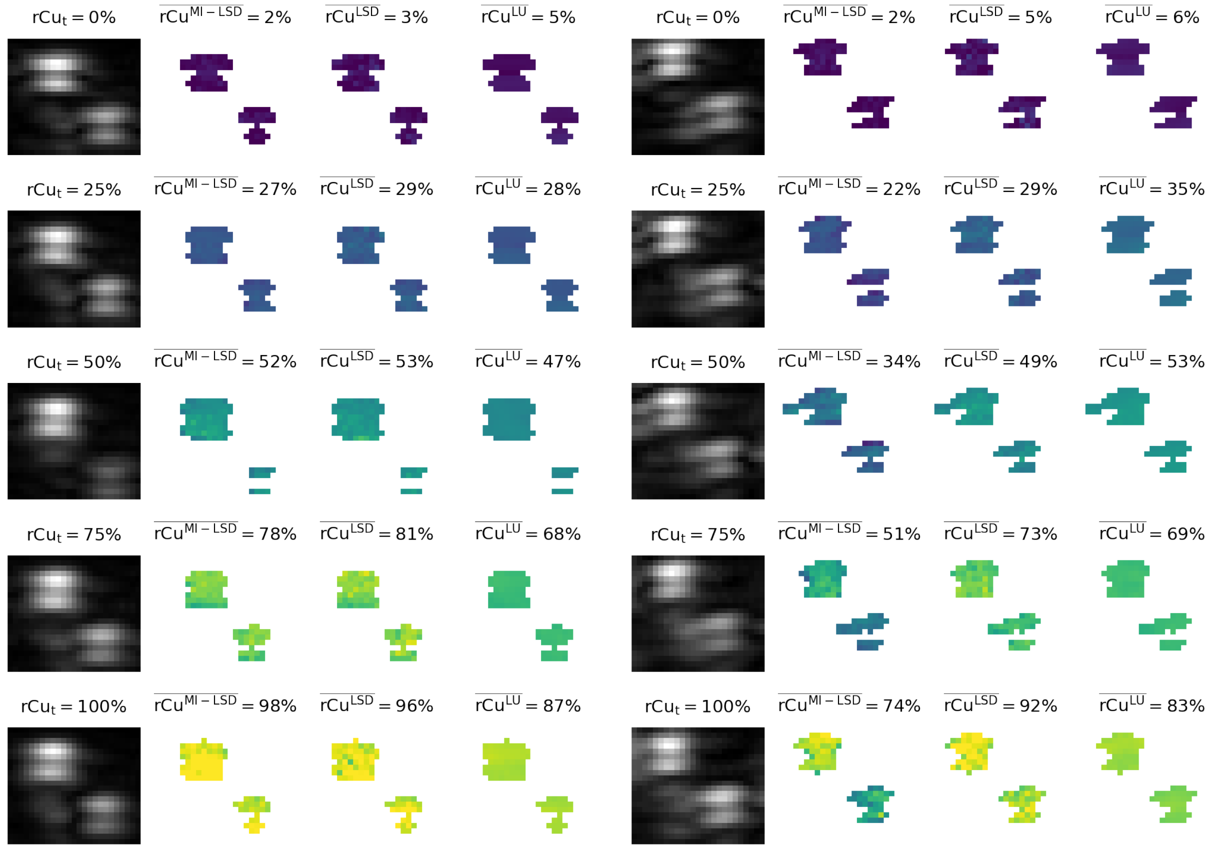

Figure 42: $rCu_{bg} = 75\%$, svf = 0.5%, NN with dropout – mean rCu estimates for Left: upper, Right: lower tubes. Showing mean signal and MI-LSD, LSD and LU estimates

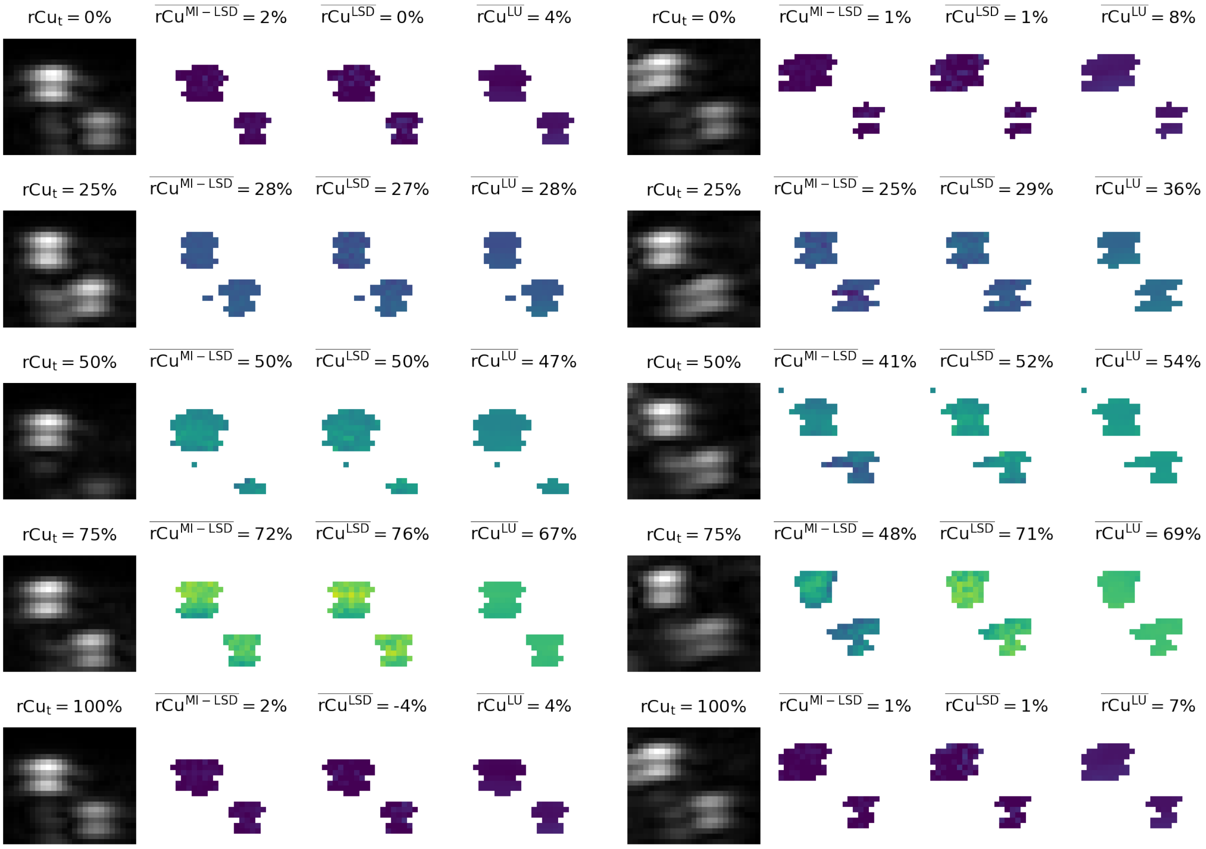

Figure 43: $rCu_{bg} = 50\%$, svf $= 0.5\%$, NN with dropout – mean rCu estimates for Left: upper, Right: lower tubes. Showing mean signal and MI-LSD, LSD and LU estimates. *Note: Due to a phantom positioning oversight, the* $rCu_t = 100\%$ *measurement had an actual rCu of 0% and was omitted from further analysis.*

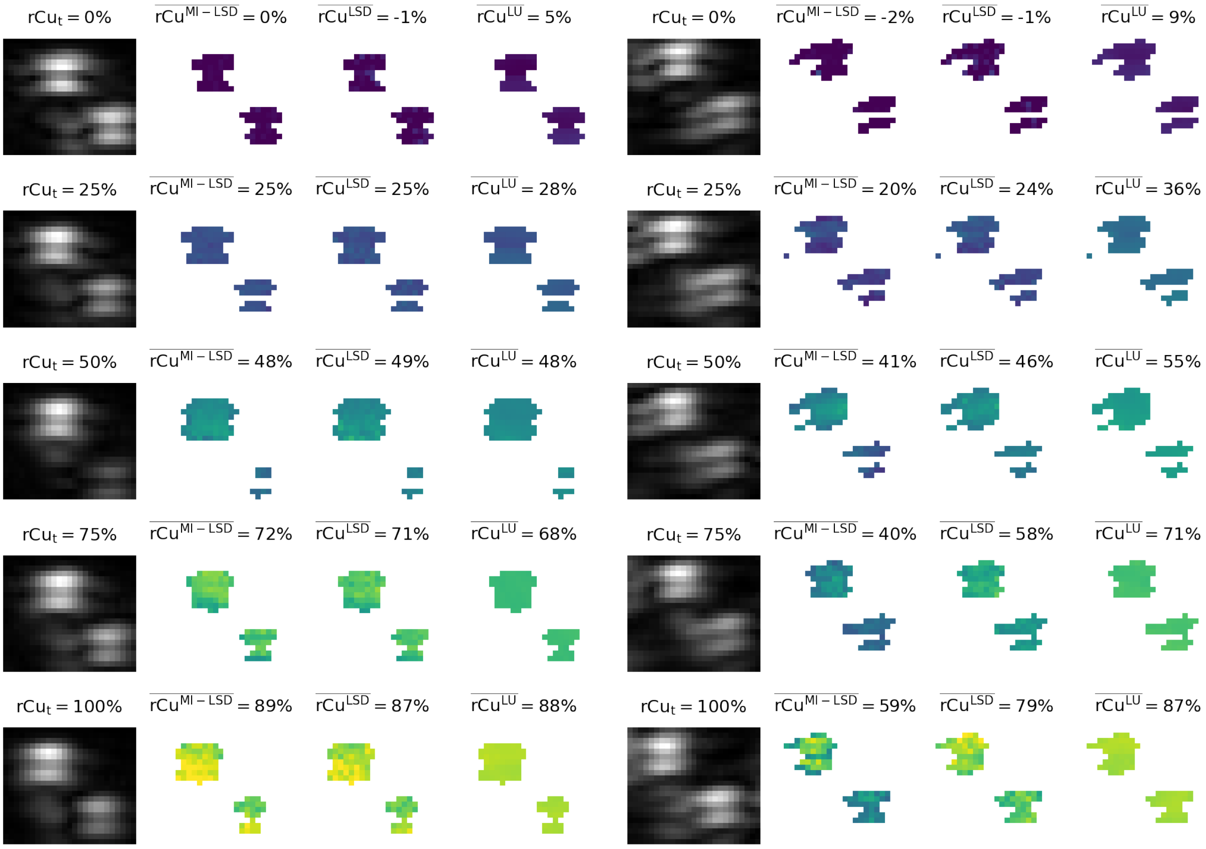

Figure 44: $rCu_{bg} = 25\%$, svf $= 0.5\%$, NN with dropout – mean rCu estimates for Left: upper, Right: lower tubes. Showing mean signal and MI-LSD, LSD and LU estimates

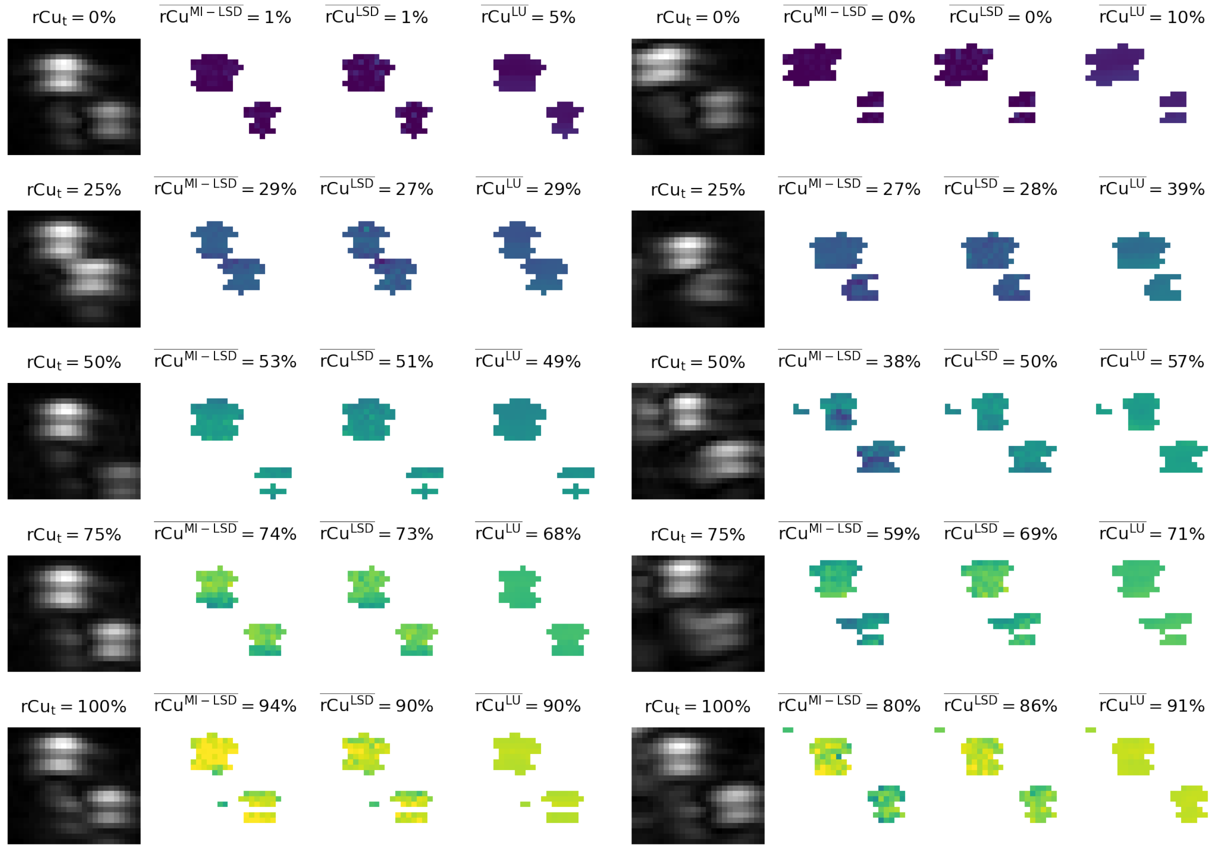

Figure 45: $rCu_{bg} = 0\%$, svf $= 0.5\%$, NN with dropout – mean rCu estimates for Left: upper, Right: lower tubes. Showing mean signal and MI-LSD, LSD and LU estimates

2.3.0.3 1.0% sulfate volume fraction (svf)

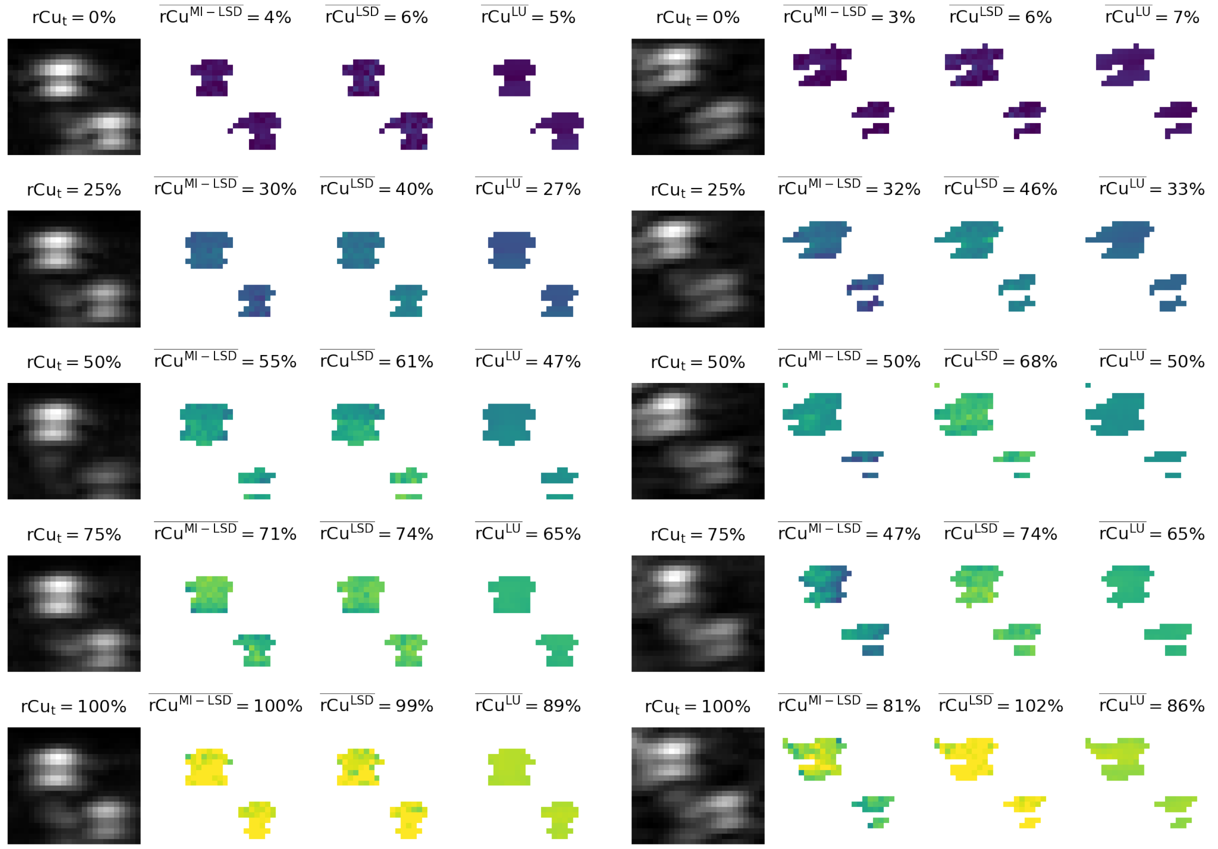

Figure 46: $rCu_{bg} = 100\%$, svf $= 1\%$, NN with dropout – mean rCu estimates for Left: upper, Right: lower tubes. Showing mean signal and MI-LSD, LSD and LU estimates

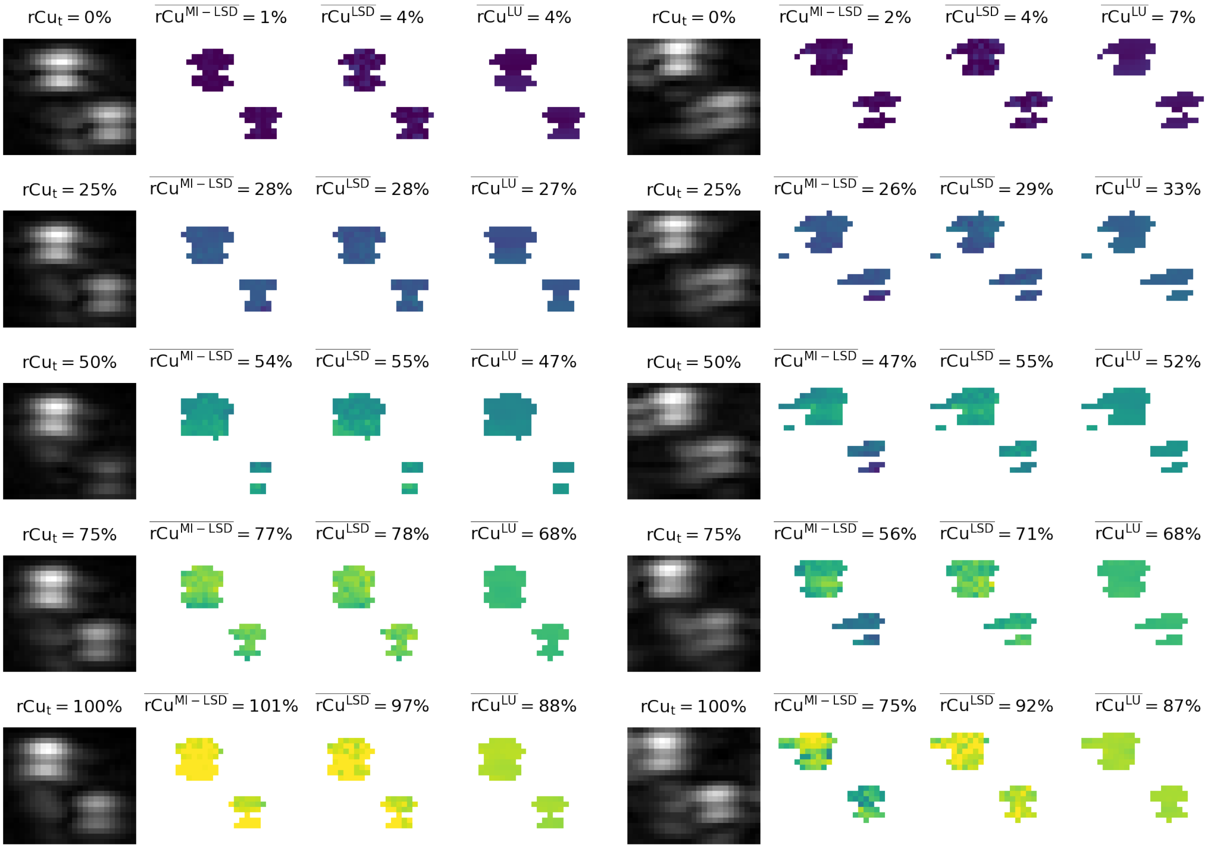

Figure 47: $rCu_{bg} = 75\%$, svf $= 1\%$, NN with dropout – mean rCu estimates for Left: upper, Right: lower tubes. Showing mean signal and MI-LSD, LSD and LU estimates

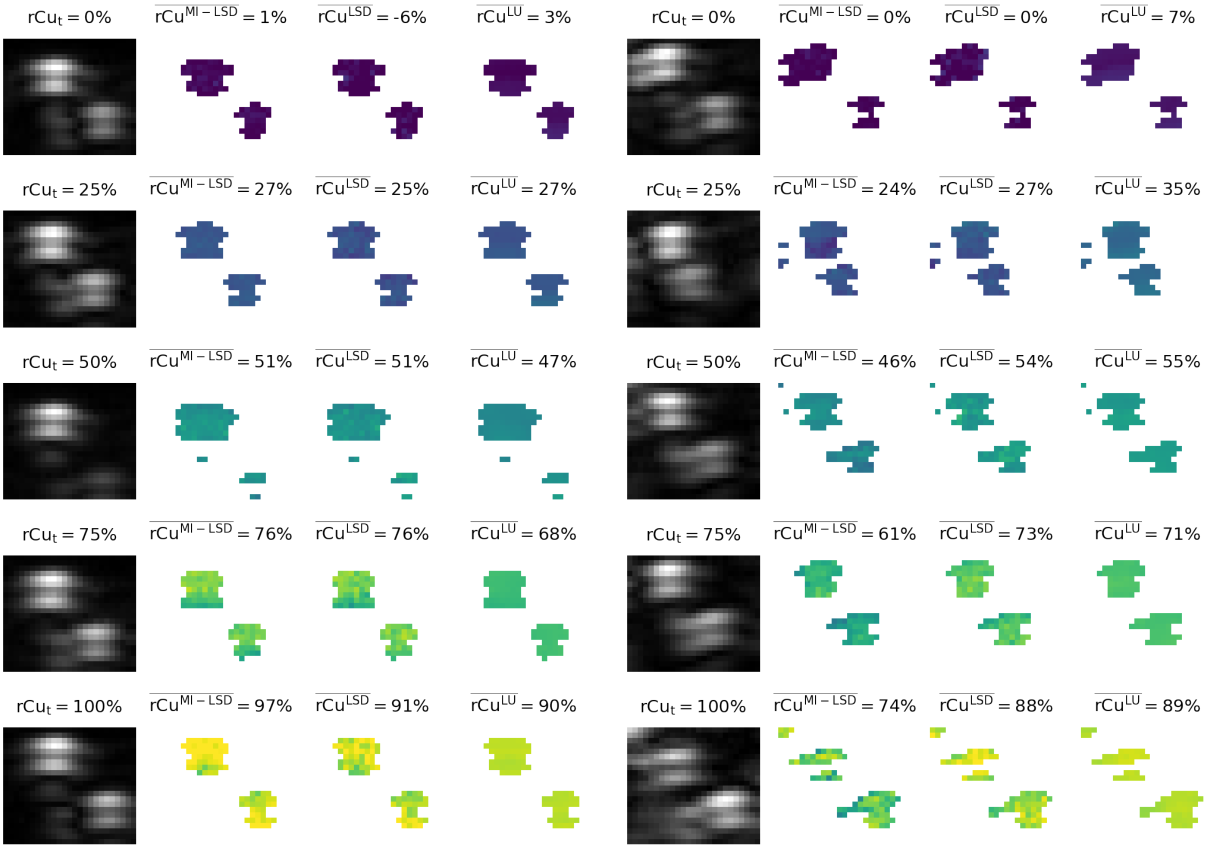

Figure 48: $rCu_{bg} = 50\%$, svf $= 1\%$, NN with dropout – mean rCu estimates for Left: upper, Right: lower tubes. Showing mean signal and MI-LSD, LSD and LU estimates

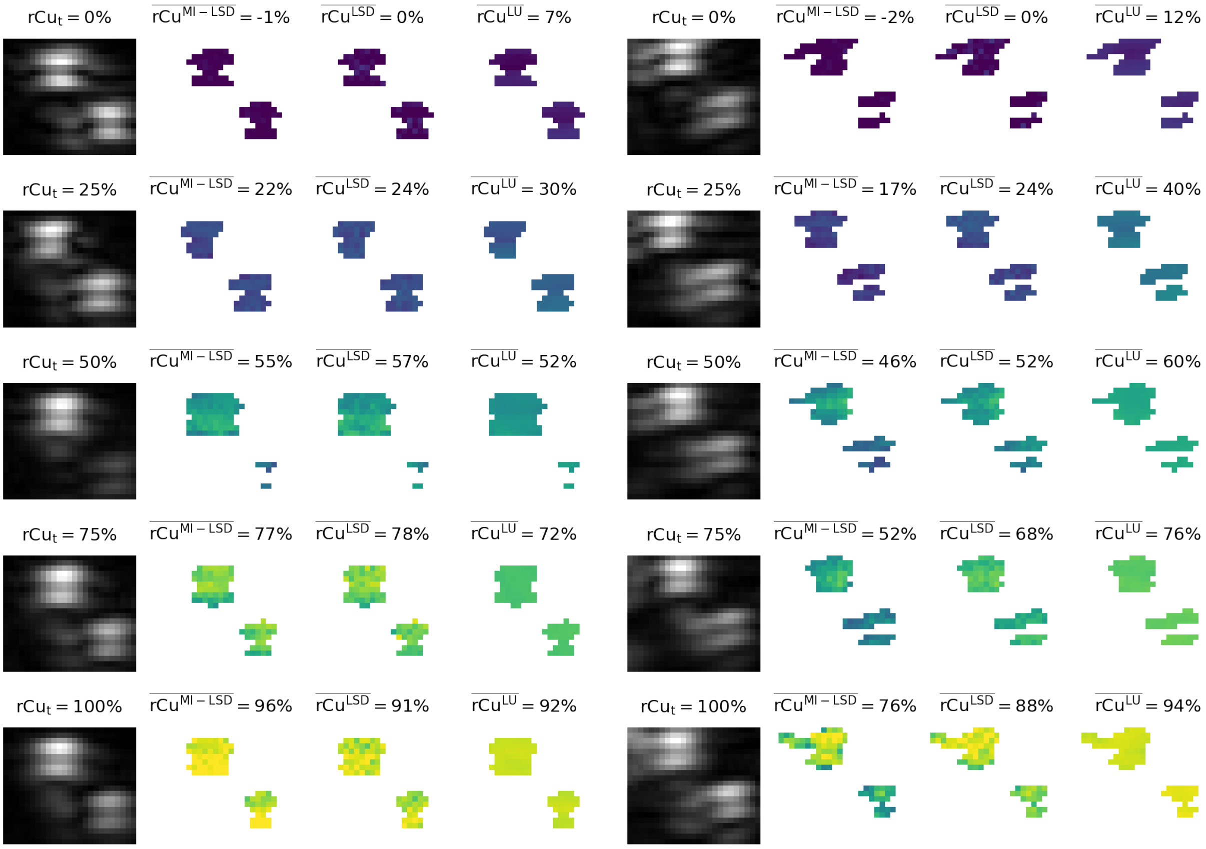

Figure 49: rCu_{bg} = 25%, svf = 1%, NN with dropout – mean rCu estimates for Left: upper, Right: lower tubes. Showing mean signal and MI-LSD, LSD and LU estimates

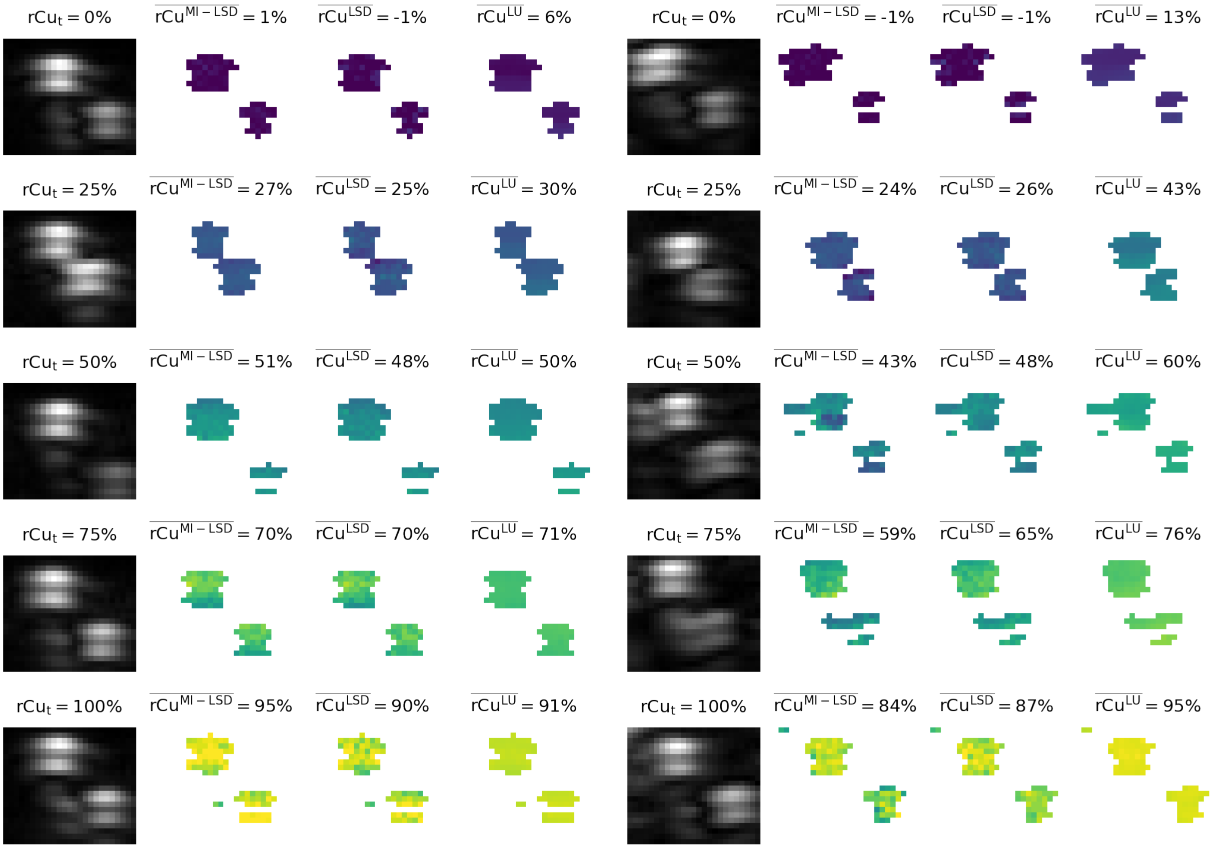

Figure 50: rCu_{bg} = 0%, svf = 1%, NN with dropout – mean rCu estimates for Left: upper, Right: lower tubes. Showing mean signal and MI-LSD, LSD and LU estimates

3 Phantom Test Set C

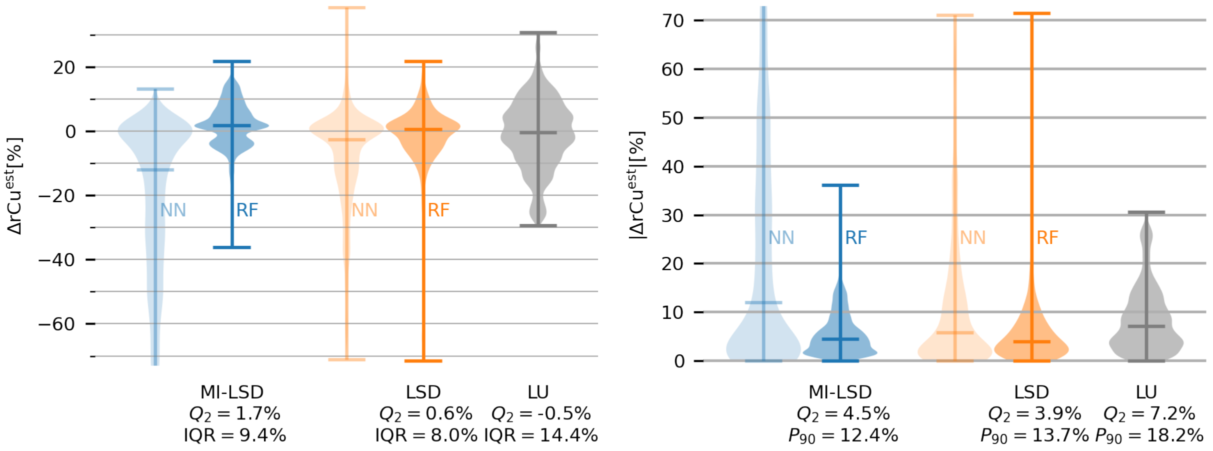

Figure 51: Estimation error distribution on the longitudinal phantom *test* set. rCu estimation errors ($\Delta \mathrm{rCu}^{\mathrm{est}}$) are shown left, their absolutes right. Blue shows the rCu estimators trained with multiple illumination learned spectral decoloring (MI-LSD), orange the estimators trained with learned spectral decoloring (LSD) and gray is the linear spectral unmixing (LU) reference. Listed: Medians Q_2, interquartile ranges (IQR) and 90 percentiles P_{90}.

3.1 Random Forest (RF)

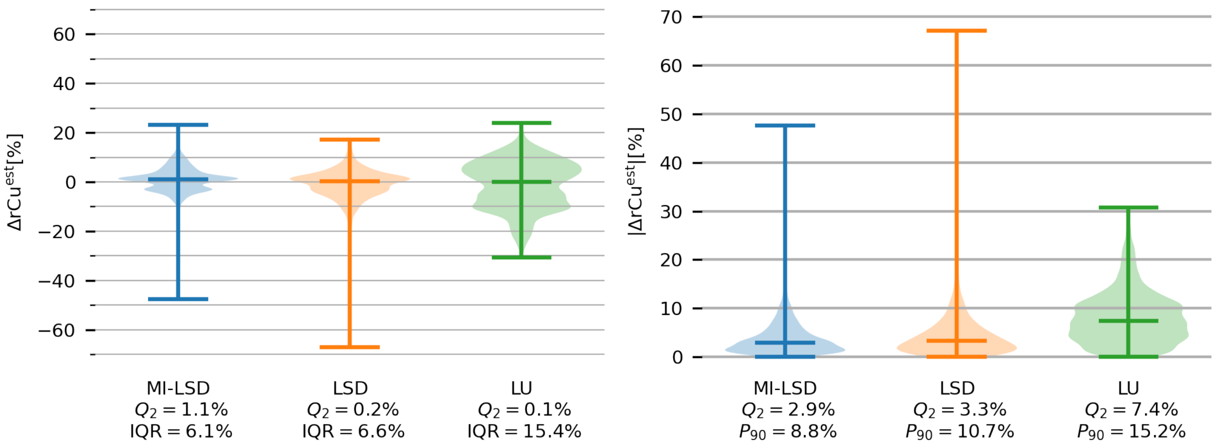

Figure 52: Random forest (RF) error distribution longitudinal phantom *test* set

3.1.0.1 Baseline – 0% sulfate volume fraction (svf)

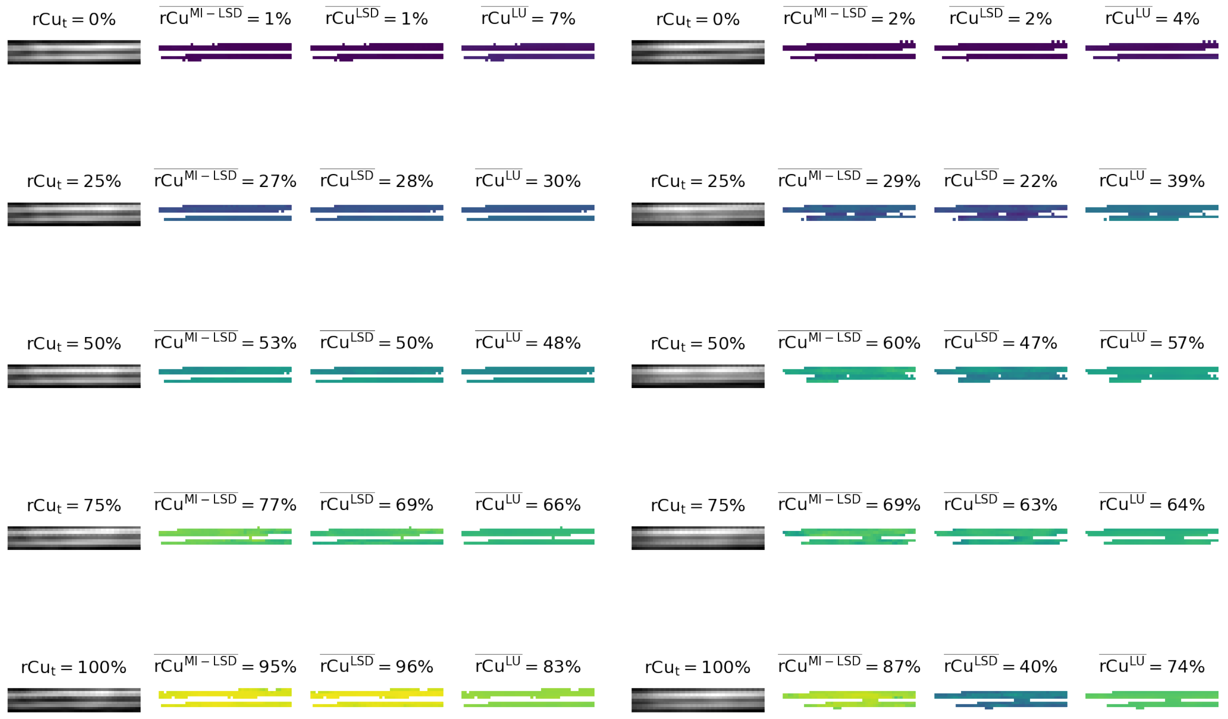

Figure 53: RF estimate on measurement 0, svf = 0% – mean rCu estimates for Left: upper, Right: lower tubes. Showing mean signal and MI-LSD, LSD and LU estimates.

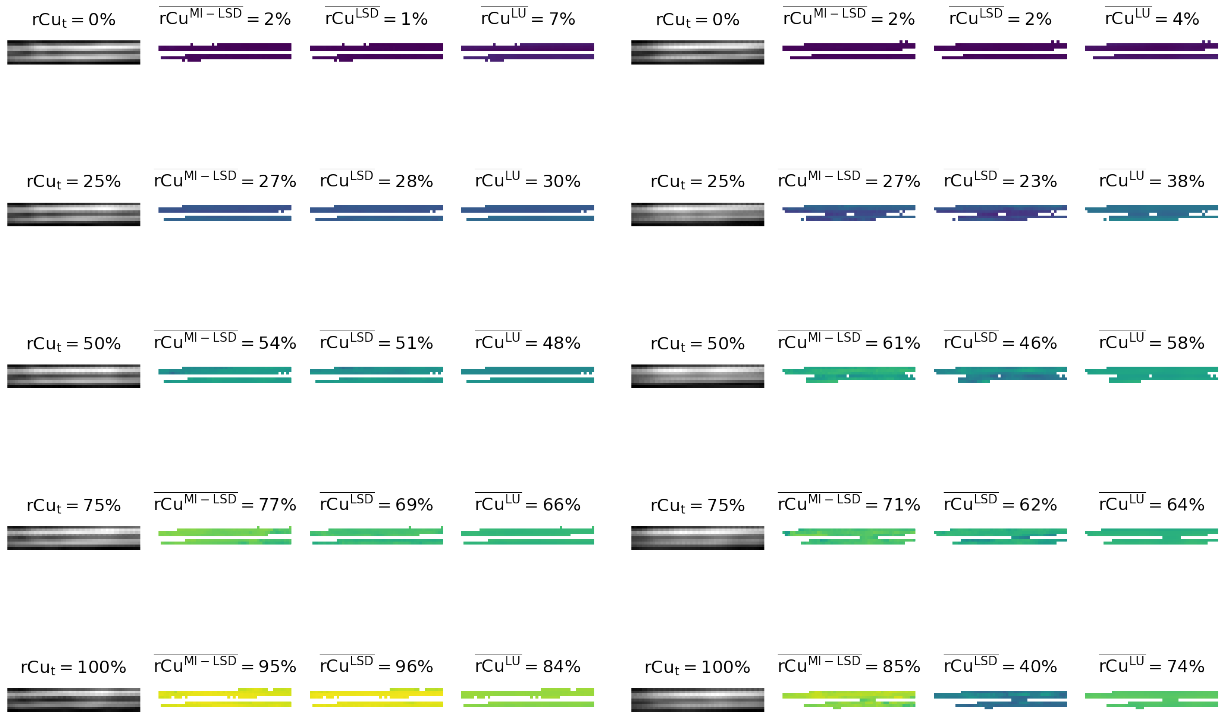

Figure 54: RF estimate on measurement 1, svf = 0% – mean rCu estimates for Left: upper, Right: lower tubes. Showing mean signal and MI-LSD, LSD and LU estimates.

3.1.0.2 1.0% sulfate volume fraction (svf)

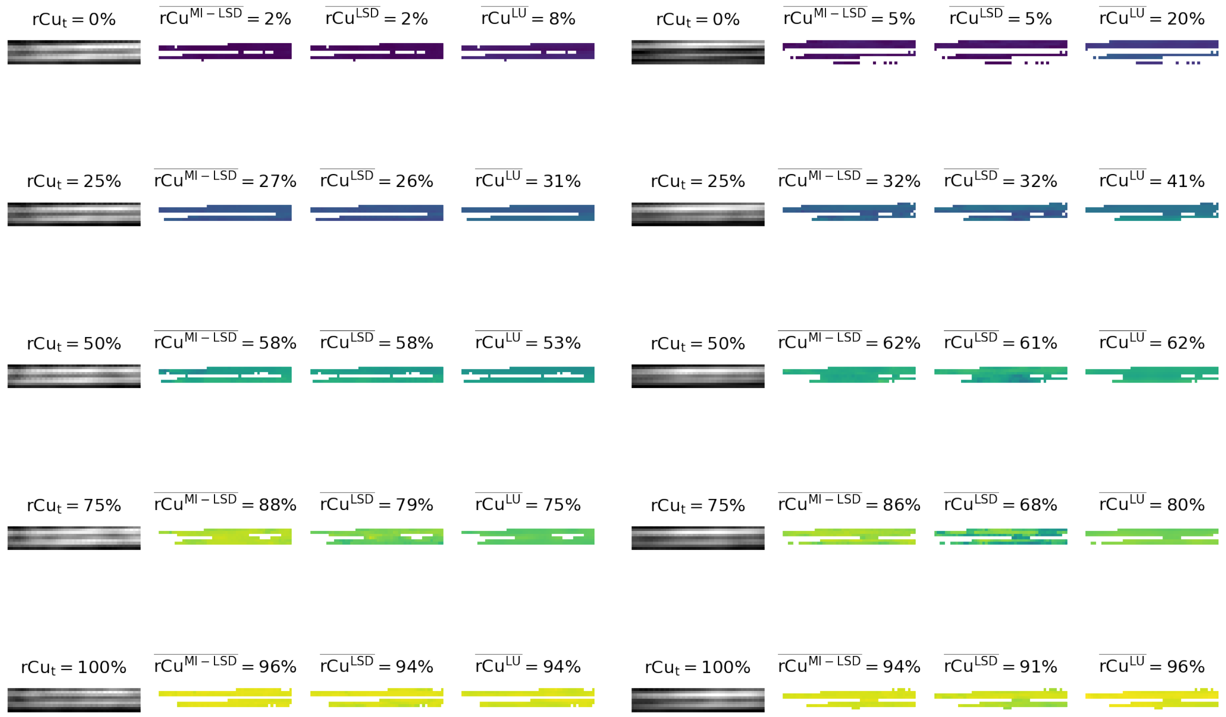

Figure 55: RF estimate on measurement 0, svf = 1.0%, background rCu=0% – mean rCu estimates for Left: upper, Right: lower tubes. Showing mean signal and MI-LSD, LSD and LU estimates.

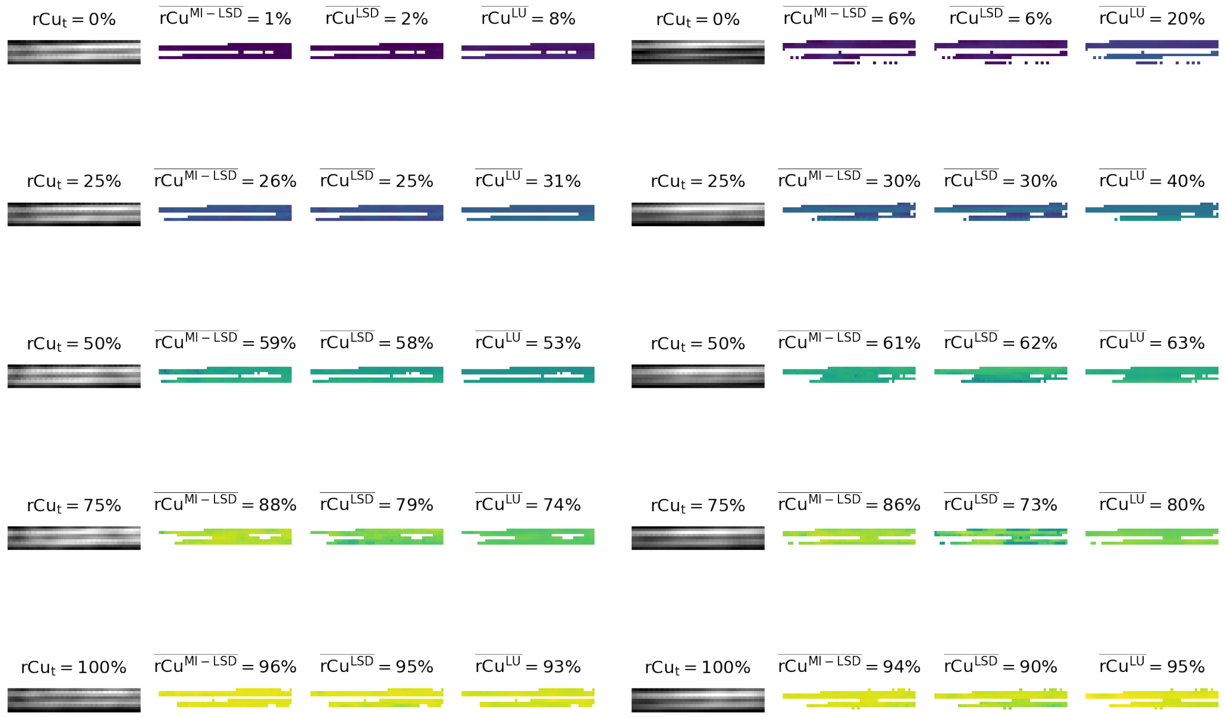

Figure 56: RF estimate on measurement 1, svf = 1.0%, background rCu=0% – mean rCu estimates for Left: upper, Right: lower tubes. Showing mean signal and MI-LSD, LSD and LU estimates.

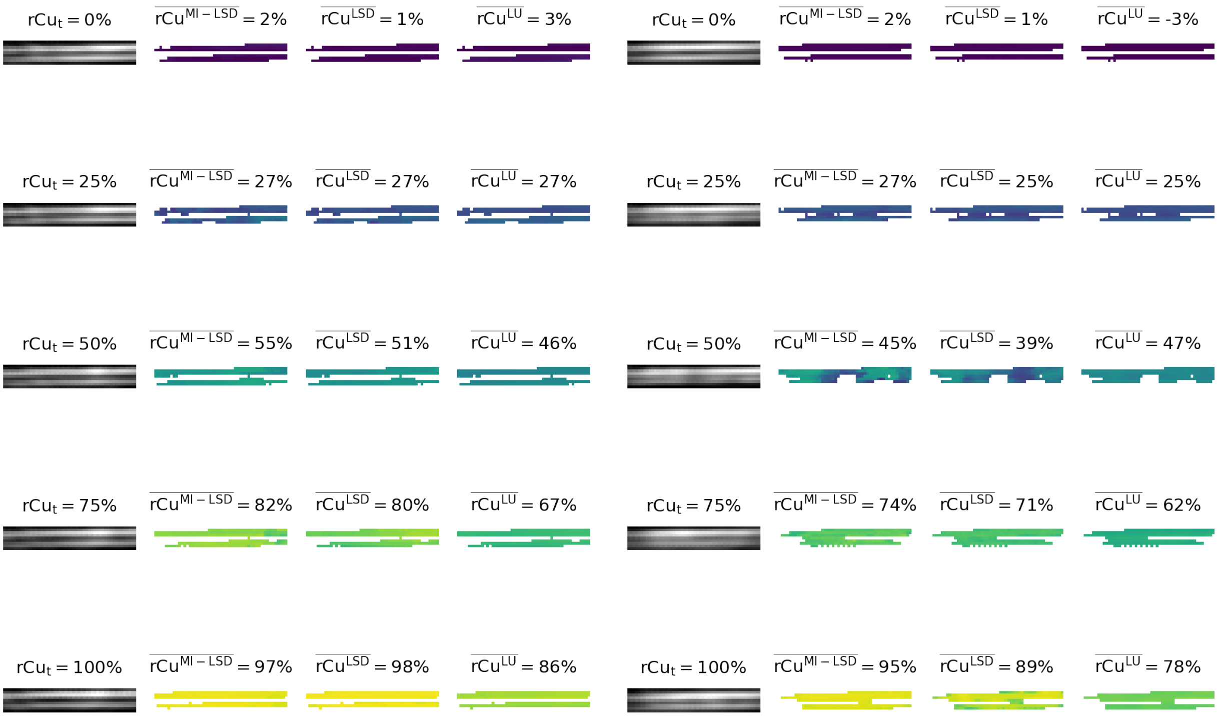

Figure 57: RF estimate on measurement 0, svf = 1.0%, background rCu=100% – mean rCu estimates for Left: upper, Right: lower tubes. Showing mean signal and MI-LSD, LSD and LU estimates

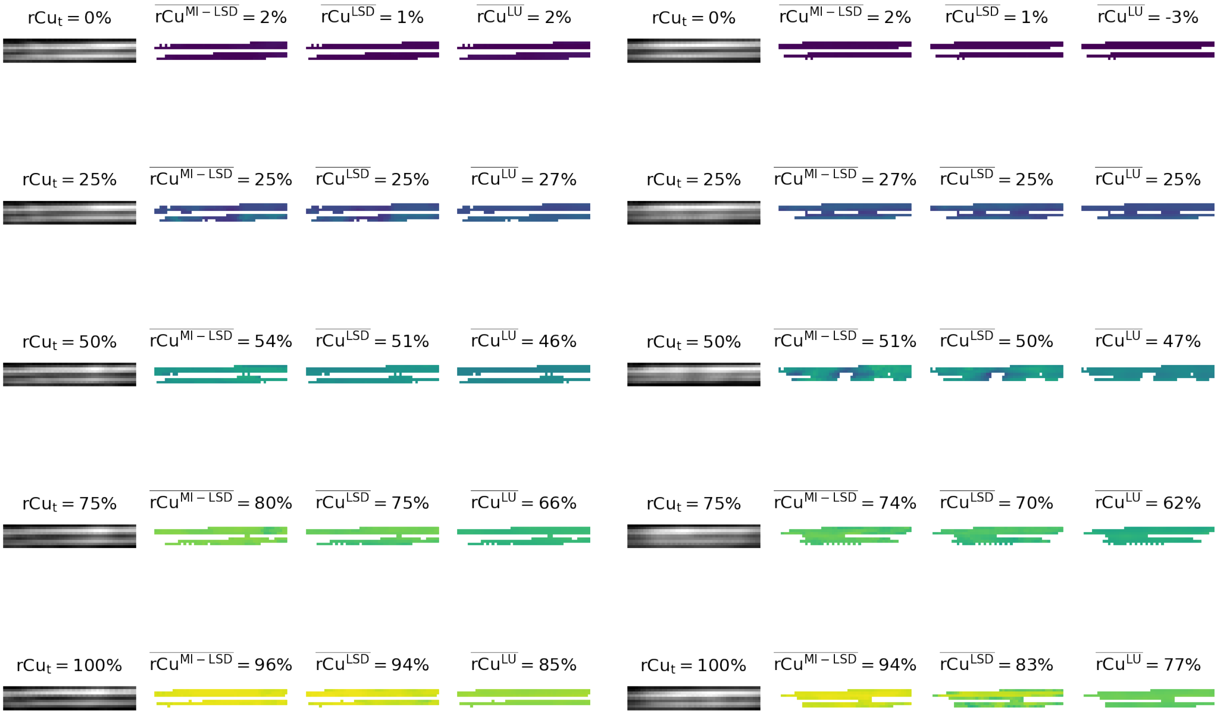

Figure 58: RF estimate on measurement 1, svf = 1.0%, background rCu=100% – mean rCu estimates for Left: upper, Right: lower tubes. Showing mean signal and MI-LSD, LSD and LU estimates.

3.2 Feed Forward Neural Network (NN) – without dropout

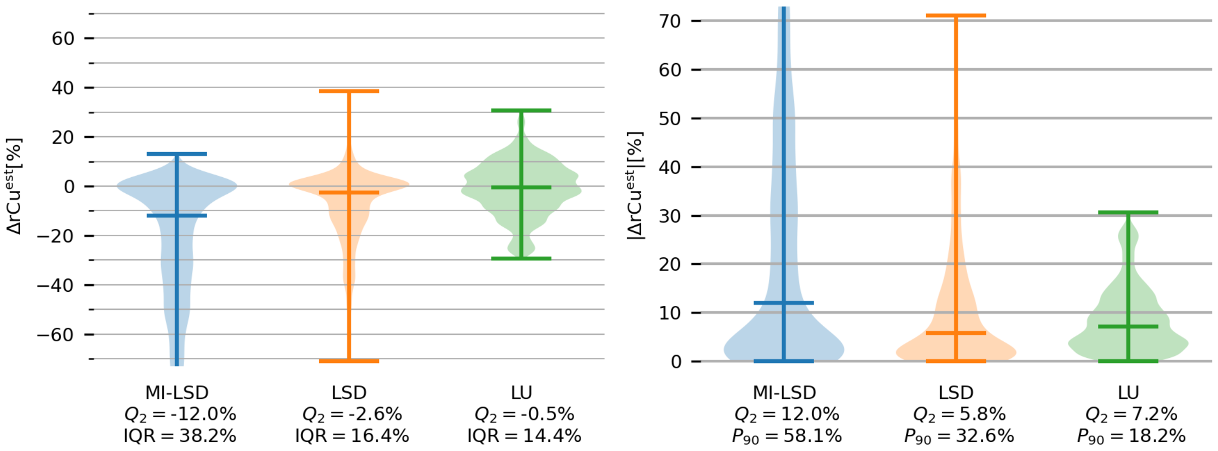

Figure 59: NN (no dropout) error distribution longitudinal phantom *test* set

3.2.0.1 Baseline – 0% sulfate volume fraction (svf)

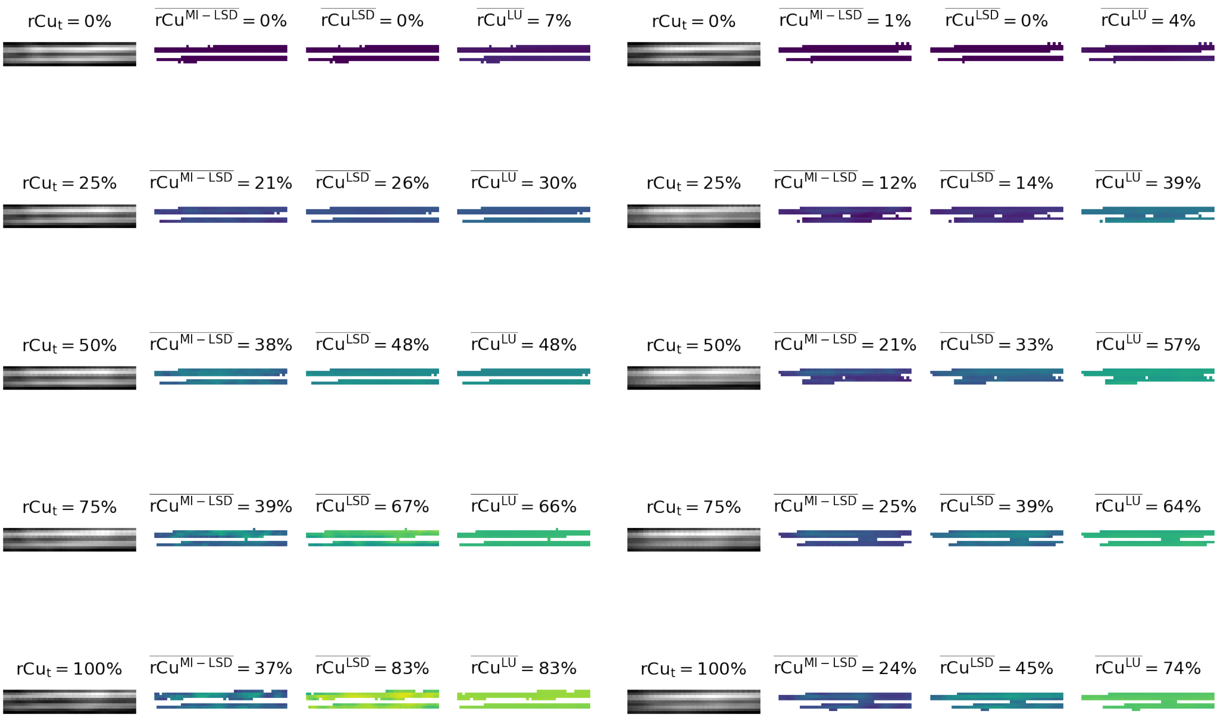

Figure 60: NN (no dropout) estimate on measurement 0, svf = 0% – mean rCu estimates for Left: upper, Right: lower tubes. Showing mean signal and MI-LSD, LSD and LU estimates.

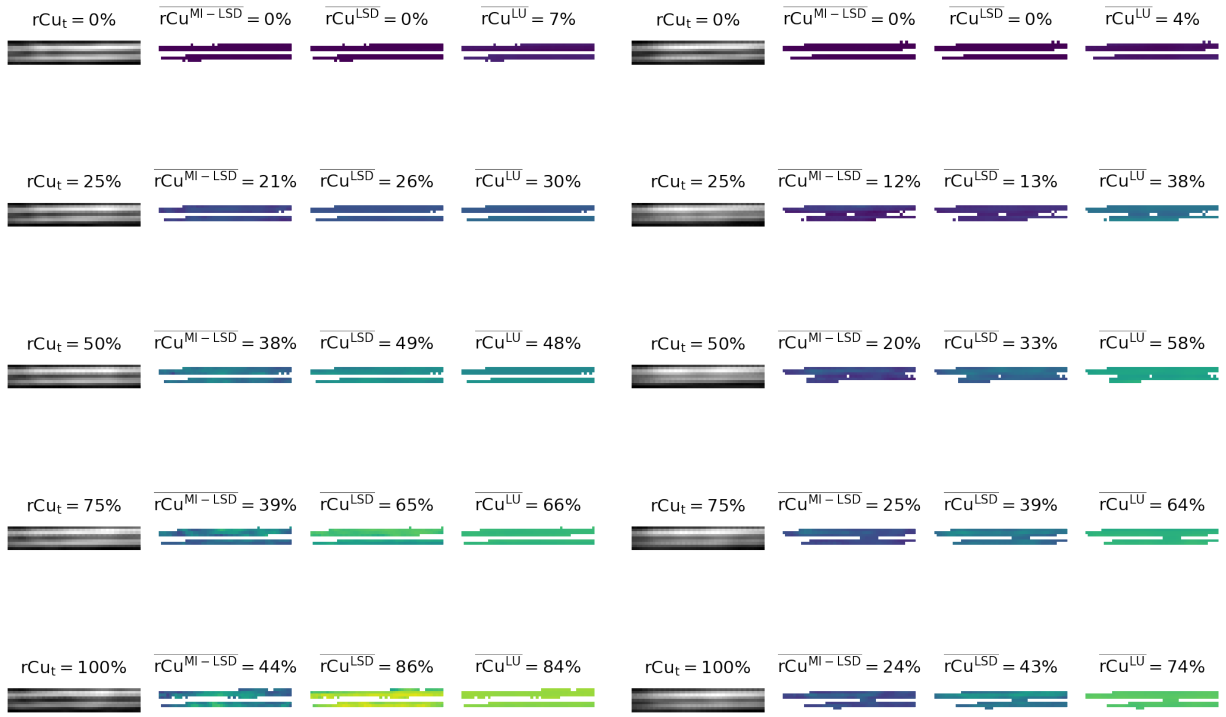

Figure 61: NN (no dropout) estimate on measurement 1, svf = 0% – mean rCu estimates for Left: upper, Right: lower tubes. Showing mean signal and MI-LSD, LSD and LU estimates.

3.2.0.2 1.0% sulfate volume fraction (svf)

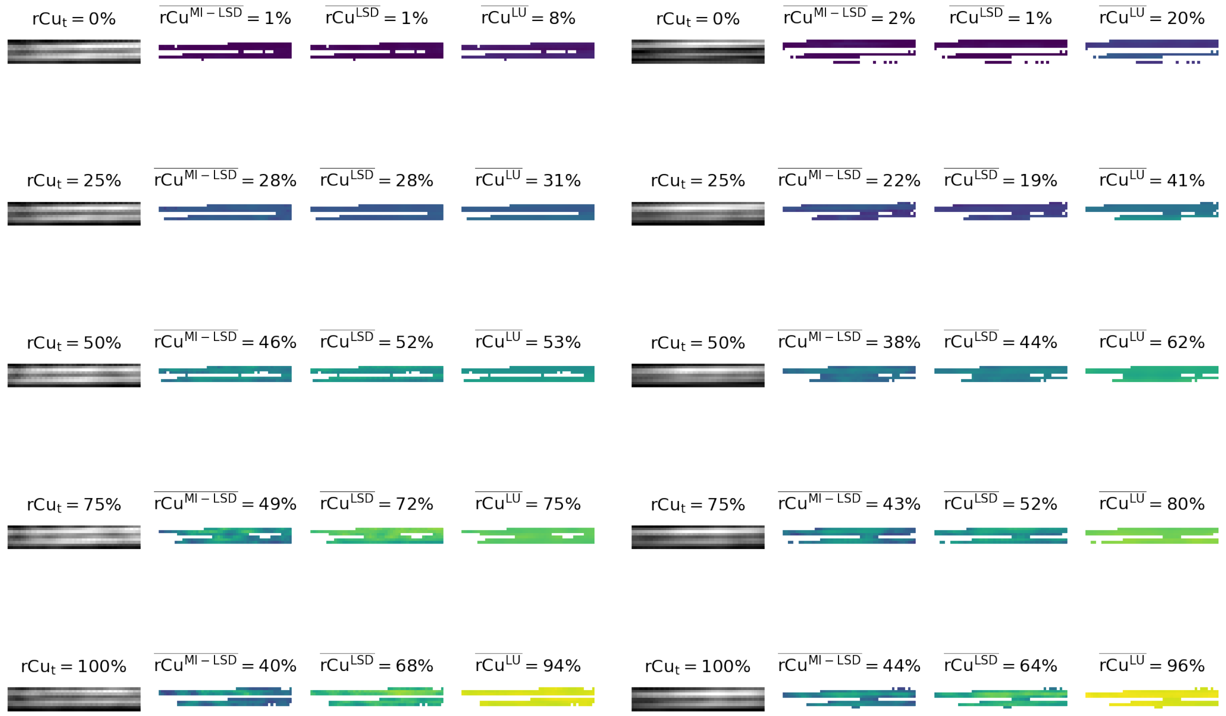

Figure 62: NN (no dropout) estimate on measurement 0, svf = 1.0%, background rCu=0% – mean rCu estimates for Left: upper, Right: lower tubes. Showing mean signal and MI-LSD, LSD and LU estimates.

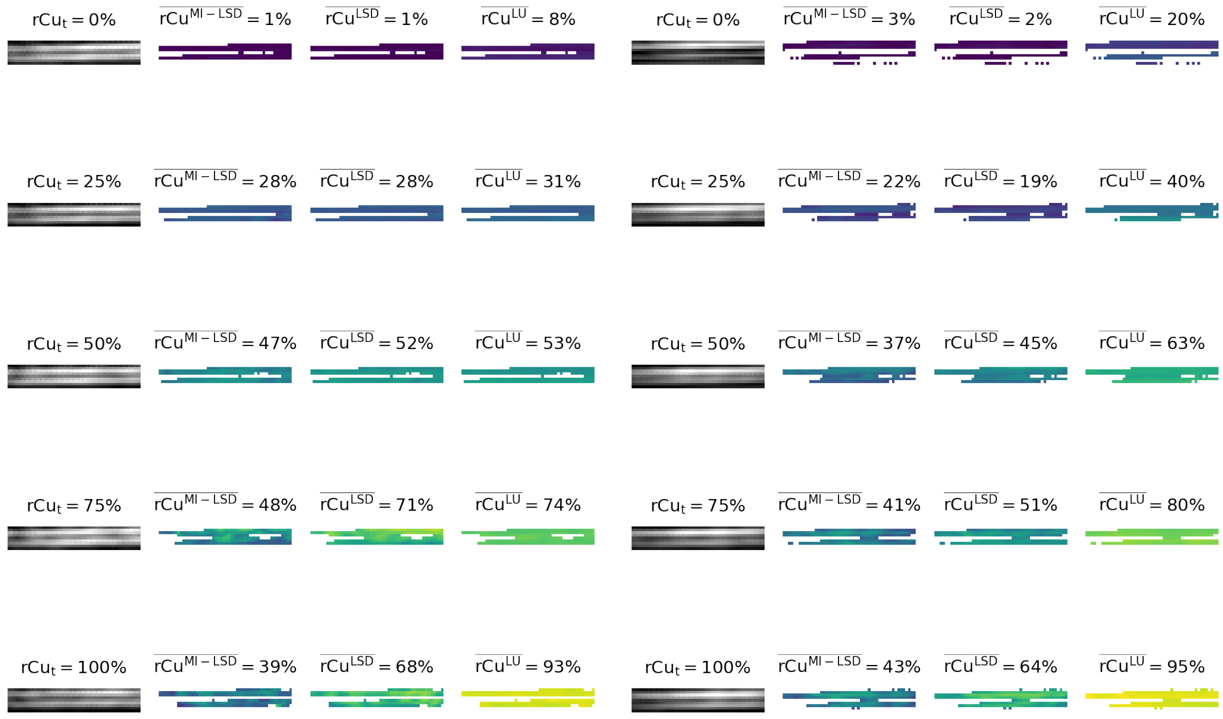

Figure 63: NN (no dropout) estimate on measurement 1, svf = 1.0%, background rCu=0% – mean rCu estimates for Left: upper, Right: lower tubes. Showing mean signal and MI-LSD, LSD and LU estimates.

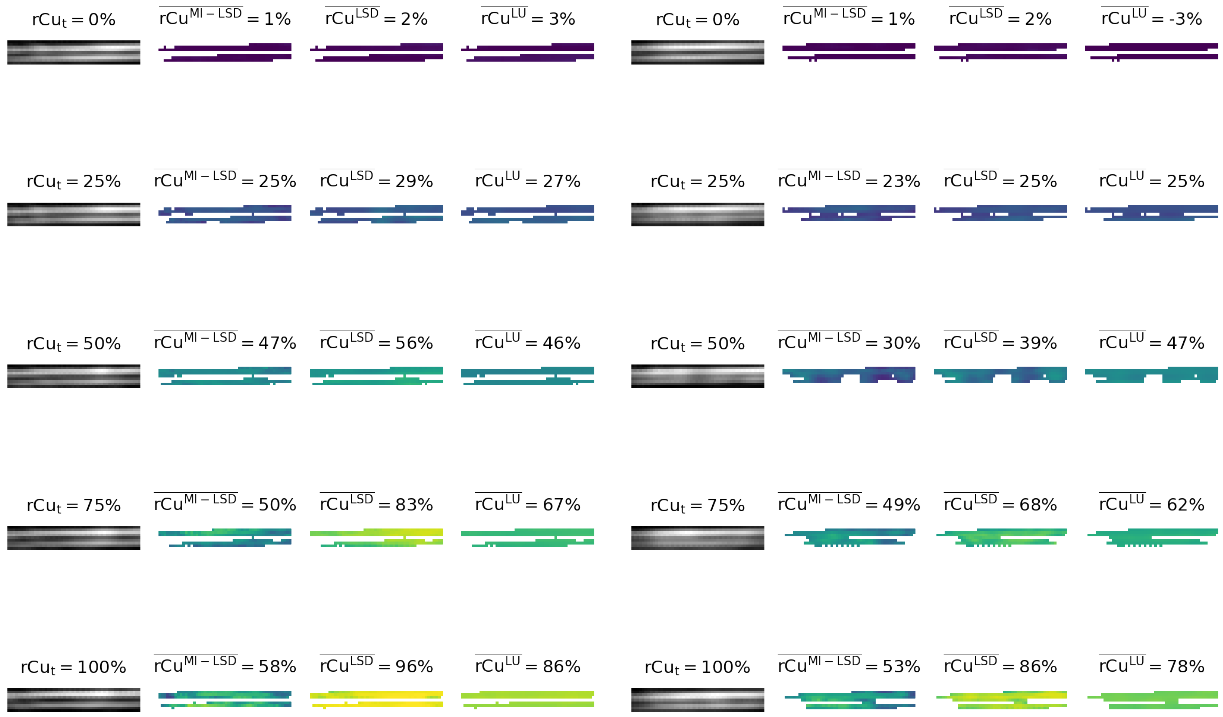

Figure 64: NN (no dropout) estimate on measurement 0, svf = 1.0%, background rCu=100% – mean rCu estimates for Left: upper, Right: lower tubes. Showing mean signal and MI-LSD, LSD and LU estimates

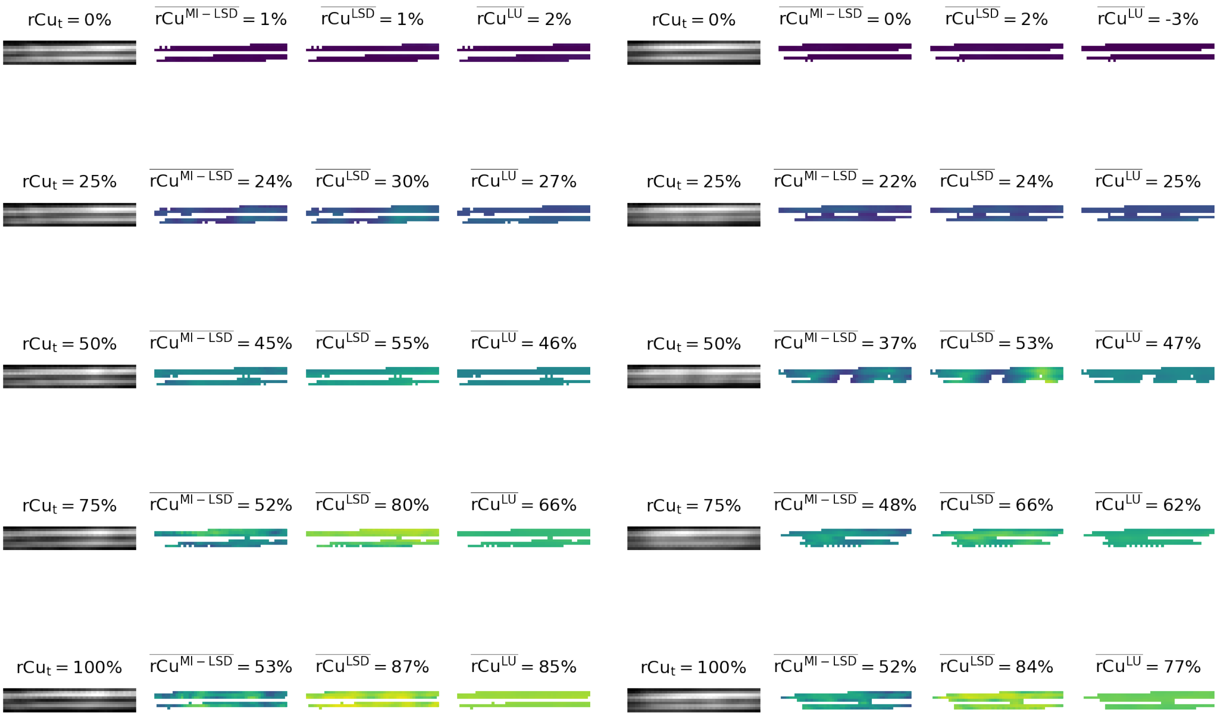

Figure 65: NN (no dropout) estimate on measurement 1, svf = 1.0%, background rCu=100% – mean rCu estimates for Left: upper, Right: lower tubes. Showing mean signal and MI-LSD, LSD and LU estimates.

3.3 Feed Forward Neural Network (NN) – with dropout

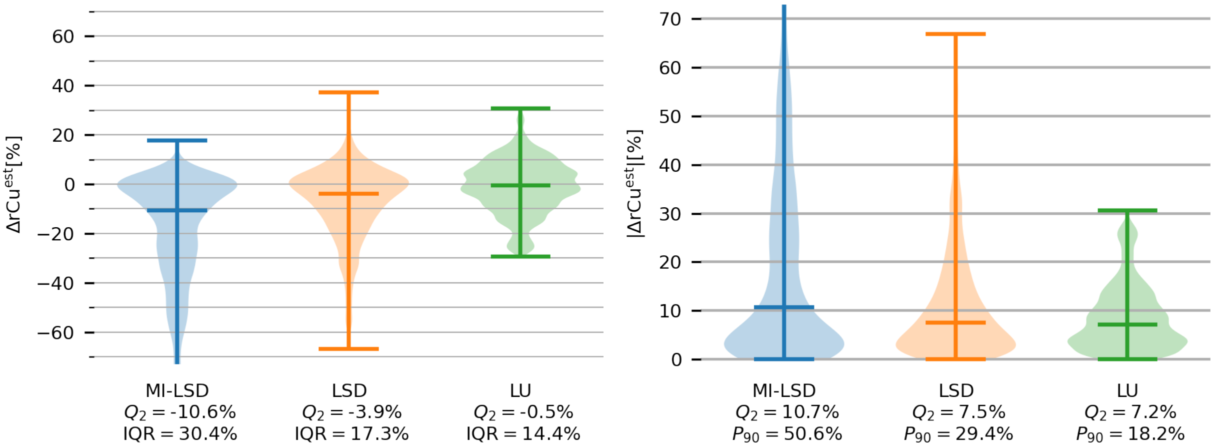

Figure 66: NN (with dropout) error distribution longitudinal phantom *test* set

3.3.0.1 Baseline – 0% sulfate volume fraction (svf)

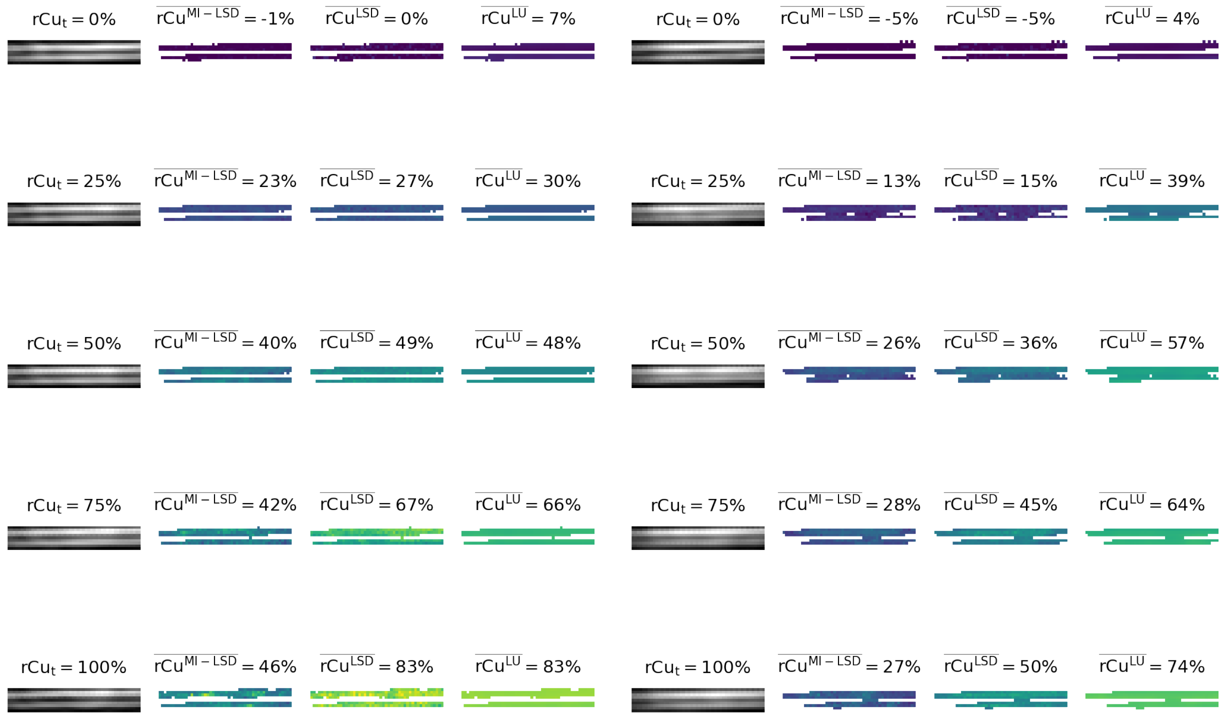

Figure 67: NN (with dropout) estimate on measurement 0, svf = 0% – mean rCu estimates for Left: upper, Right: lower tubes. Showing mean signal and MI-LSD, LSD and LU estimates.

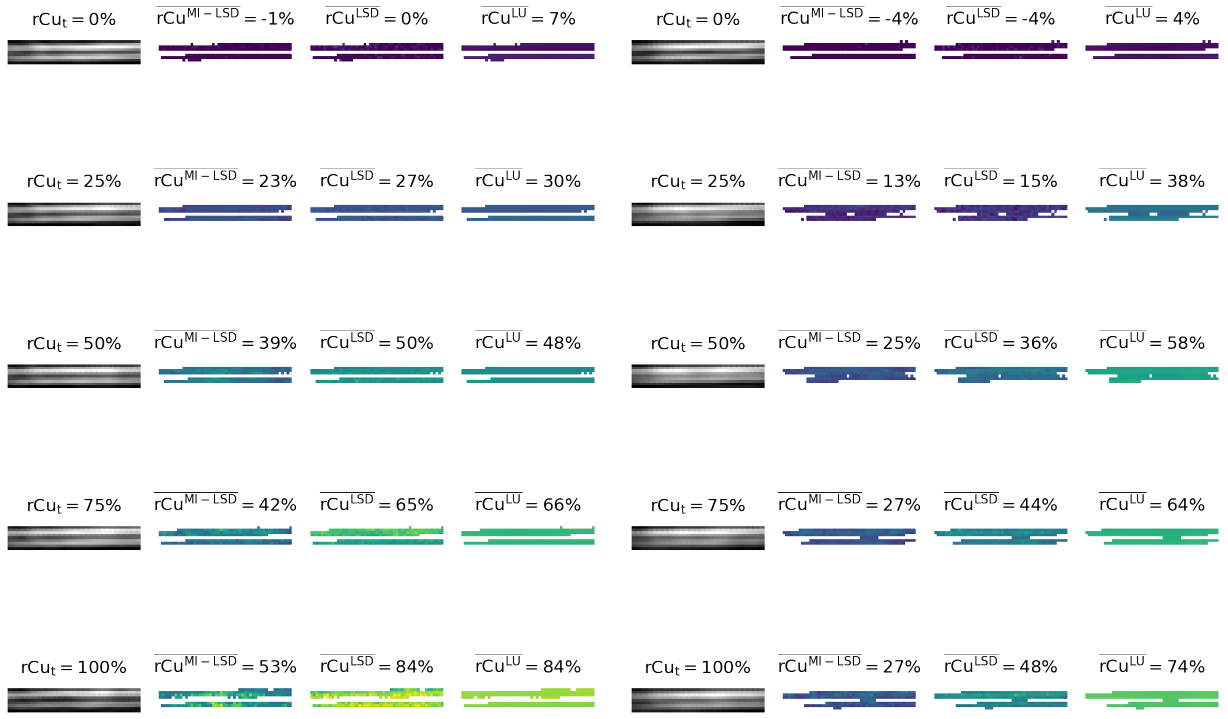

Figure 68: NN (with dropout) estimate on measurement 1, svf = 0% – mean rCu estimates for Left: upper, Right: lower tubes. Showing mean signal and MI-LSD, LSD and LU estimates.

3.3.0.2 1.0% sulfate volume fraction (svf)

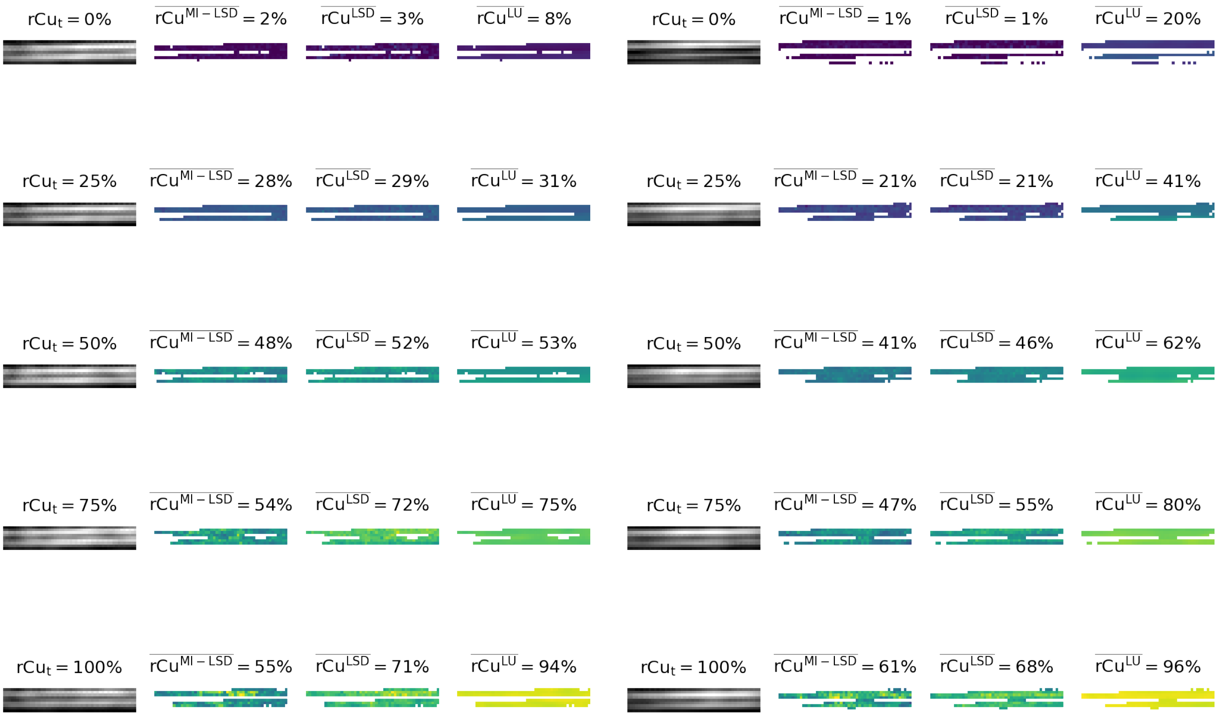

Figure 69: NN (with dropout) estimate on measurement 0, svf = 1.0%, background rCu=0% – mean rCu estimates for Left: upper, Right: lower tubes. Showing mean signal and MI-LSD, LSD and LU estimates.

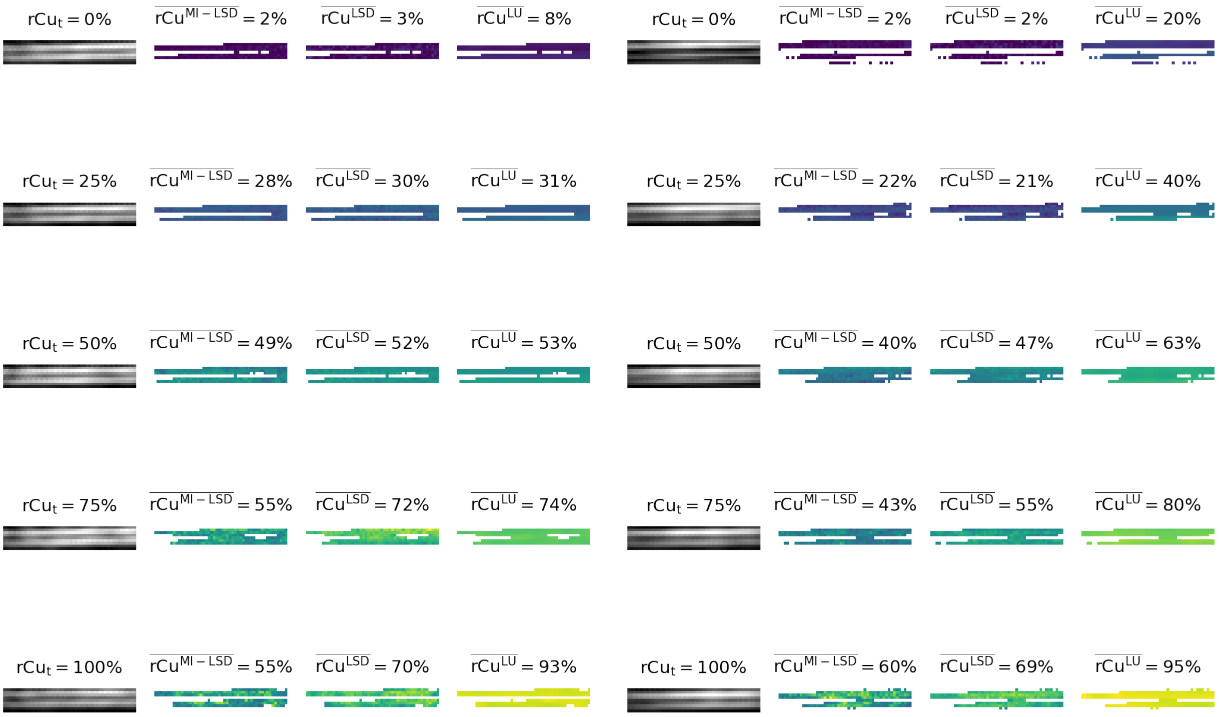

Figure 70: NN (with dropout) estimate on measurement 1, svf = 1.0%, background rCu=0% – mean rCu estimates for Left: upper, Right: lower tubes. Showing mean signal and MI-LSD, LSD and LU estimates.

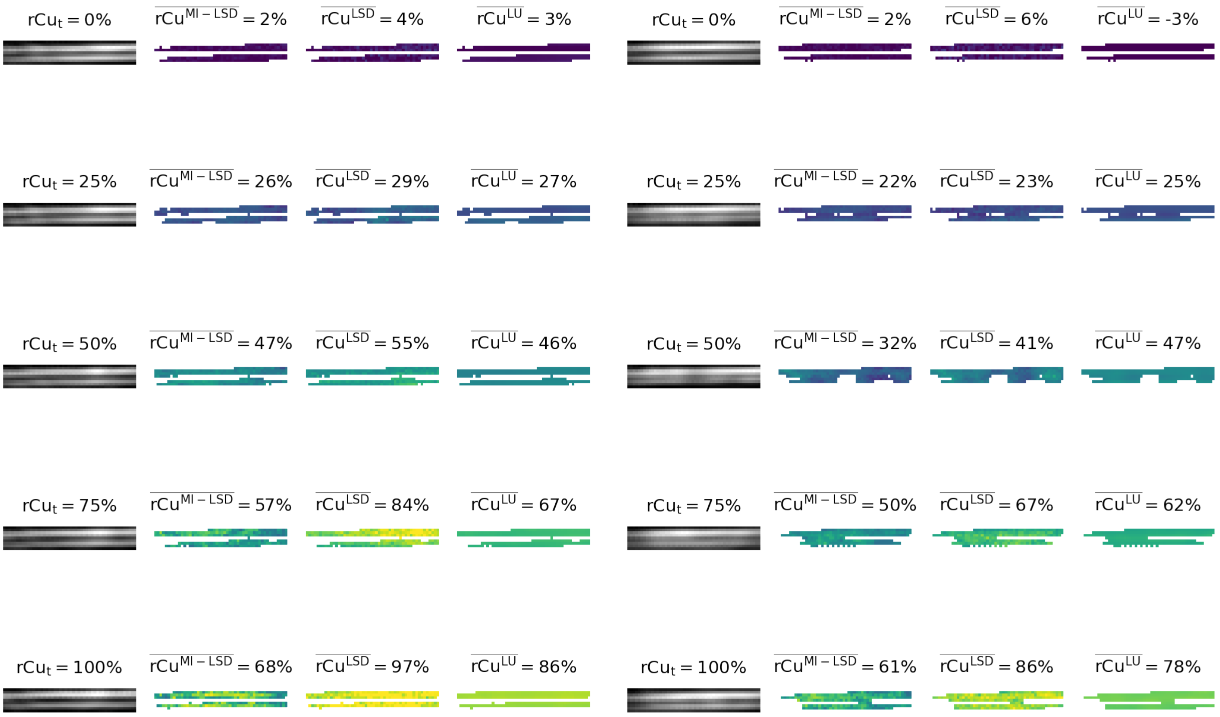

Figure 71: NN (with dropout) estimate on measurement 0, svf = 1.0%, background rCu=100% – mean rCu estimates for Left: upper, Right: lower tubes. Showing mean signal and MI-LSD, LSD and LU estimates

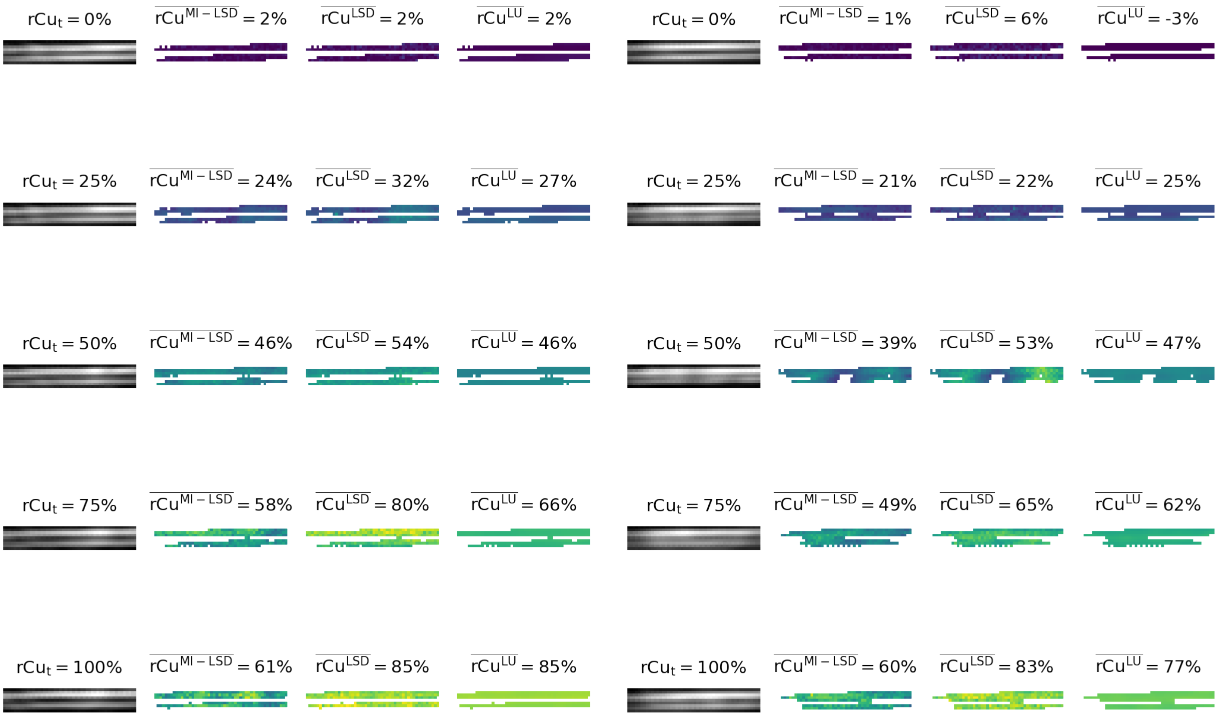

Figure 72: NN (with dropout) estimate on measurement 1, svf = 1.0%, background rCu=100% – mean rCu estimates for Left: upper, Right: lower tubes. Showing mean signal and MI-LSD, LSD and LU estimates.

4 Phantom Validation Set (A)

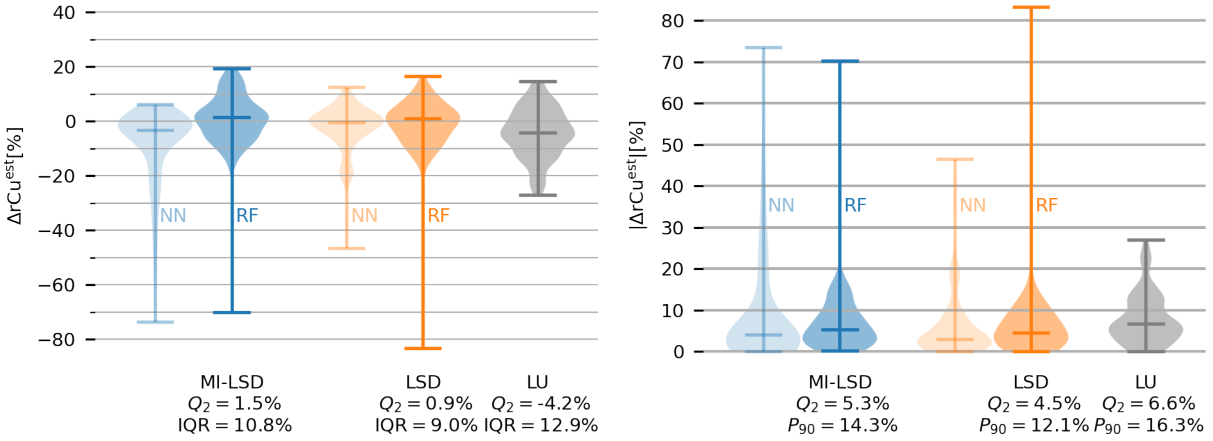

Figure 73: Estimation error distribution on the initial phantom *validation* set. rCu estimation errors ($\Delta \mathrm{rCu}^{\mathrm{est}}$) are shown left, their absolutes right. Blue shows the rCu estimators trained with multiple illumination learned spectral decoloring (MI-LSD), orange the estimators trained with learned spectral decoloring (LSD) and gray is the linear spectral unmixing (LU) reference. Listed: Medians Q_2, interquartile ranges (IQR) and 90 percentiles P_{90}.

4.1 Random Forest (RF)

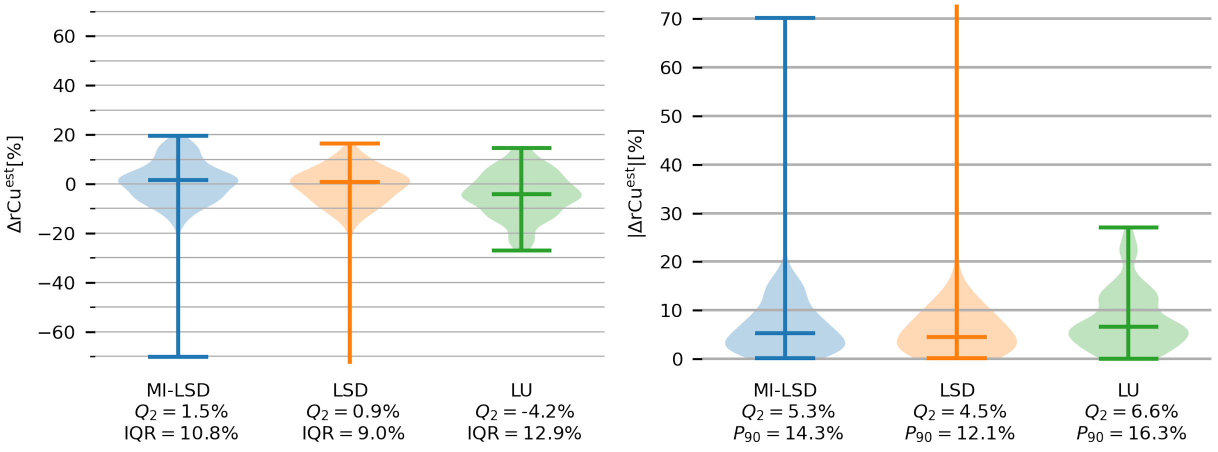

Figure 74: Estimation error distribution on the *validation* phantom, RF. rCu estimation errors ($\Delta \mathrm{rCu}^{\mathrm{est}}$) are shown left, their absolutes right. Blue shows the rCu estimators trained with multiple illumination learned spectral decoloring (MI-LSD), orange the estimators trained with learned spectral decoloring (LSD) and grey is the linear spectral unmixing (LU) reference. Listed: Medians Q_2, interquartile ranges (IQR) and 90 percentiles P_{90}.

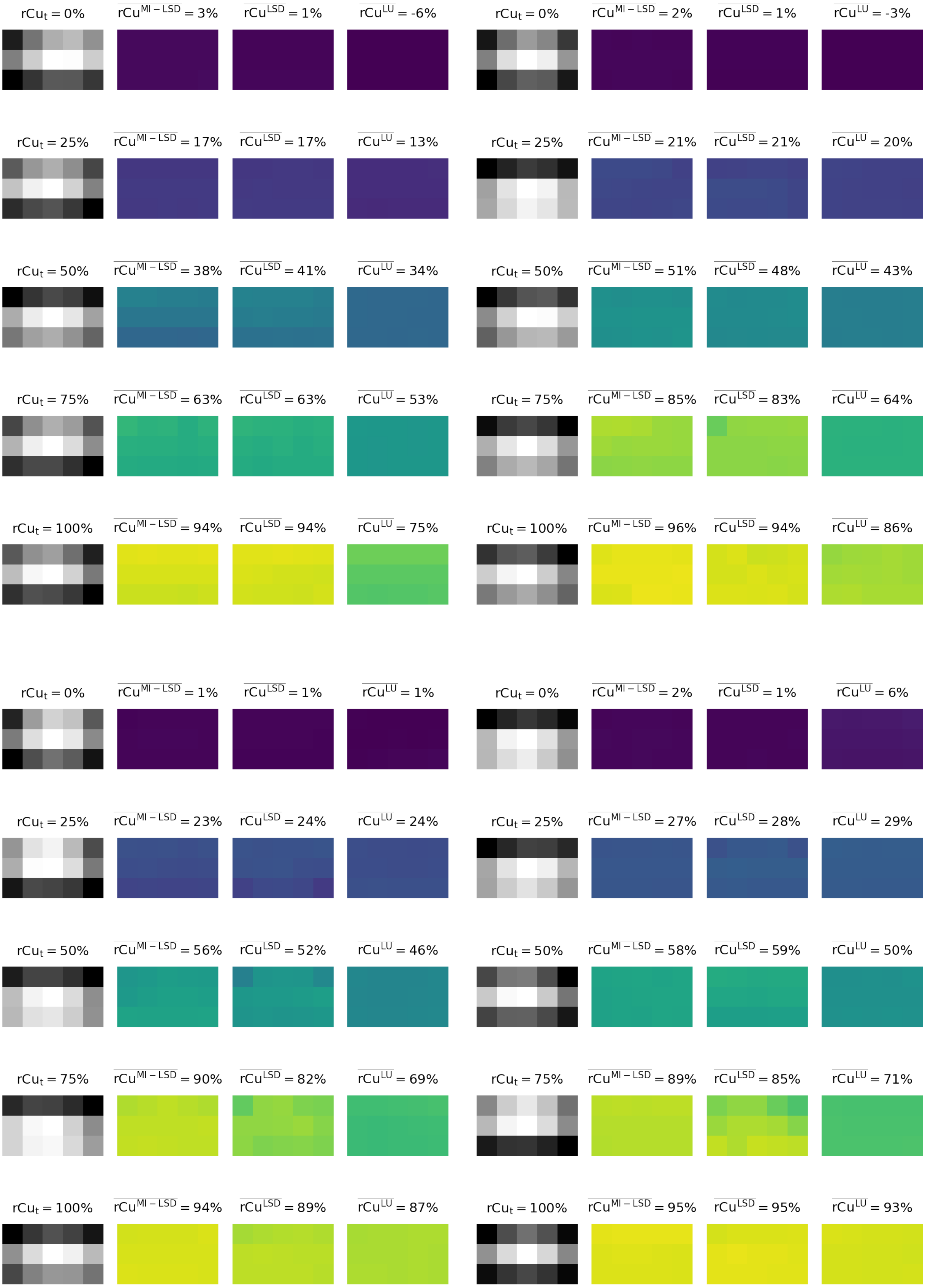

Figure 75: RF estimates on validation phantom with svf= 1.0%, background rCu=[100, 75, 50, 25]% – mean rCu estimates for tubes. Showing PA mean signal and MI-LSD, LSD and LU estimates.

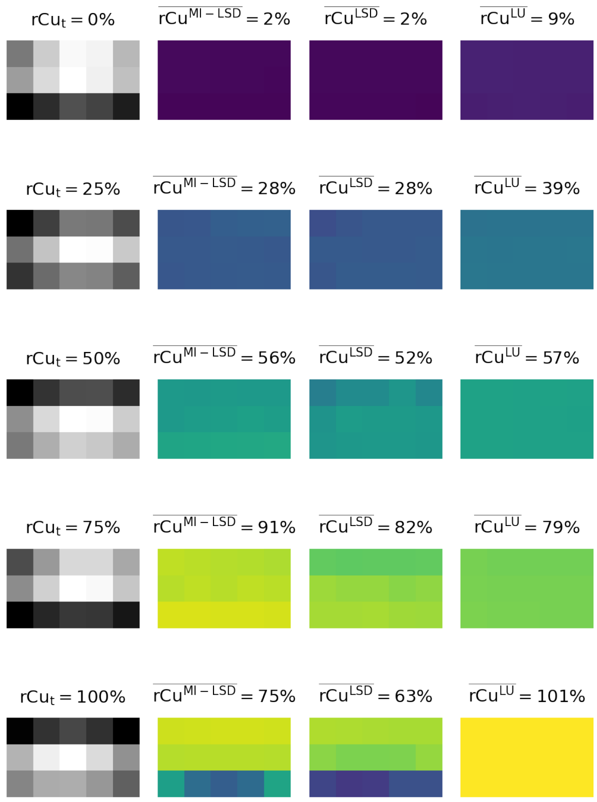

Figure 76: RF estimates on validation phantom with svf= 1.0%, background rCu=0% – mean rCu estimates for tubes. Showing PA mean signal and MI-LSD, LSD and LU estimates.

4.2 Feed Forward Neural Network (NN) – without dropout

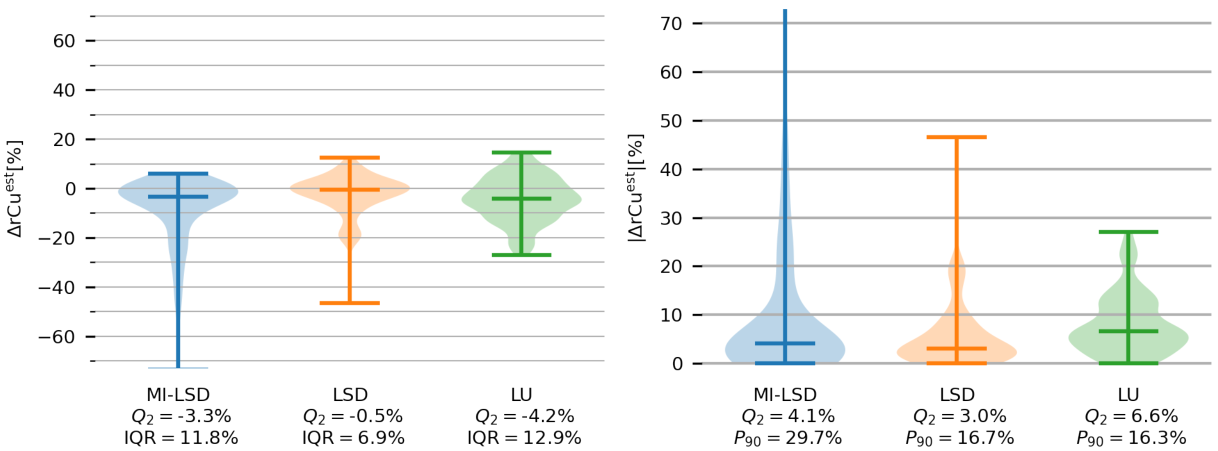

Figure 77: Estimation error distribution on the *validation* phantom, NN no dropout. rCu estimation errors ($\Delta \mathrm{rCu}^{\mathrm{est}}$) are shown left, their absolutes right. Blue shows the rCu estimators trained with multiple illumination learned spectral decoloring (MI-LSD), orange the estimators trained with learned spectral decoloring (LSD) and grey is the linear spectral unmixing (LU) reference. Listed: Medians Q_2, interquartile ranges (IQR) and 90 percentiles P_{90}.

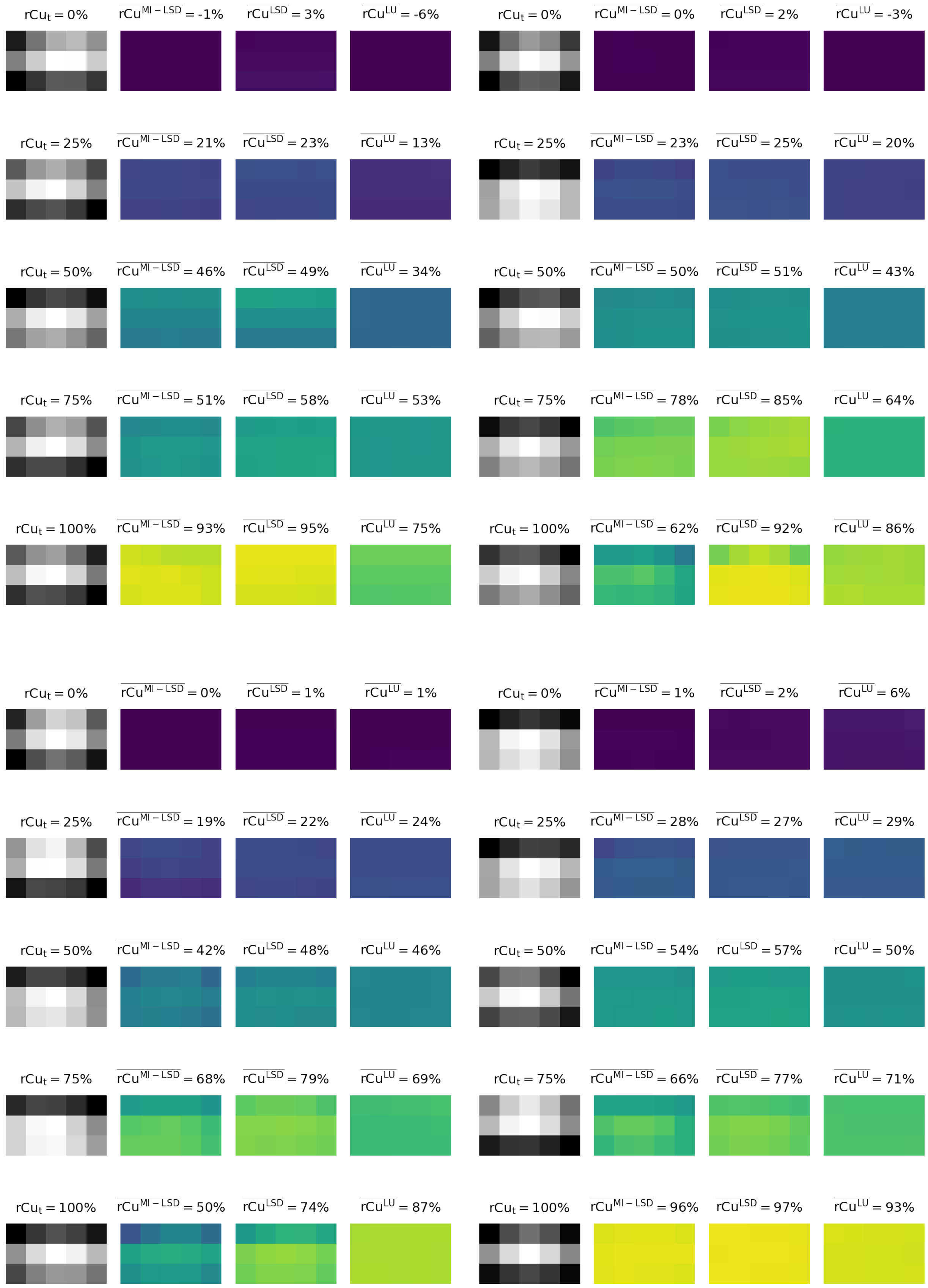

Figure 78: NN (no dropout) estimates on validation phantom with svf= 1.0%, background rCu=[100, 75, 50, 25]% – mean rCu estimates for tubes. Showing PA mean signal and MI-LSD, LSD and LU estimates.

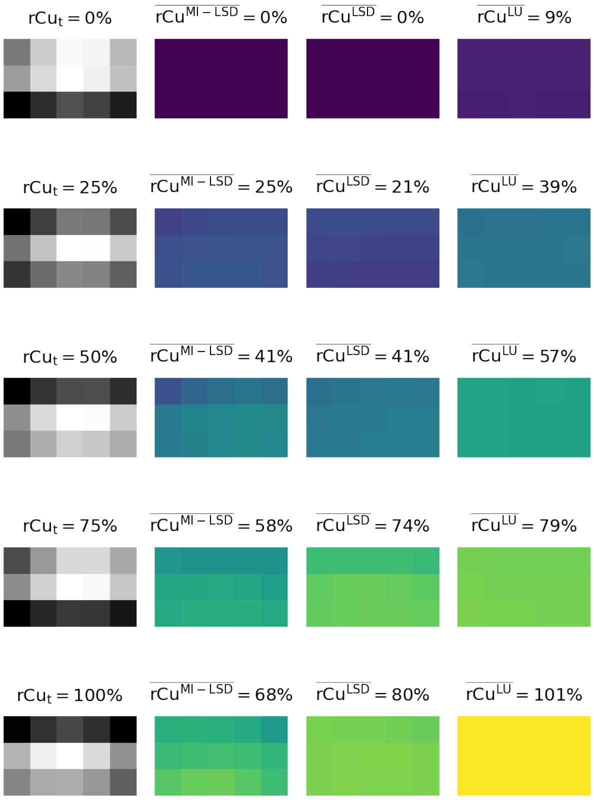

Figure 79: NN (no dropout) estimates on validation phantom with svf= 1.0%, background rCu=0% – mean rCu estimates for tubes. Showing PA mean signal and MI-LSD, LSD and LU estimates.

4.3 Feed Forward Neural Network (NN) – with dropout

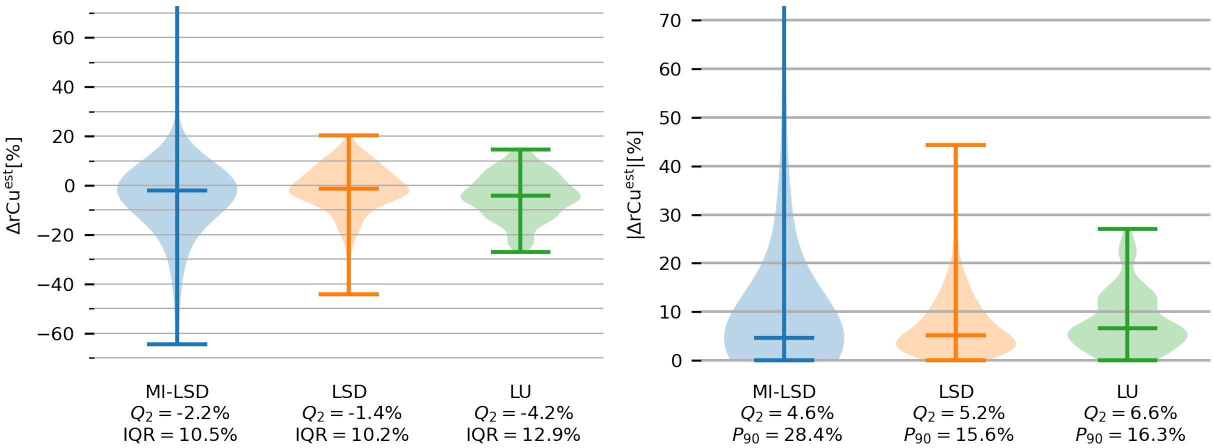

Figure 80: Estimation error distribution on the *validation* phantom, NN with dropout. rCu estimation errors (ΔrCu^{est}) are shown left, their absolutes right. Blue shows the rCu estimators trained with multiple illumination learned spectral decoloring (MI-LSD), orange the estimators trained with learned spectral decoloring (LSD) and grey is the linear spectral unmixing (LU) reference. Listed: Medians Q_2, interquartile ranges (IQR) and 90 percentiles P_{90}.

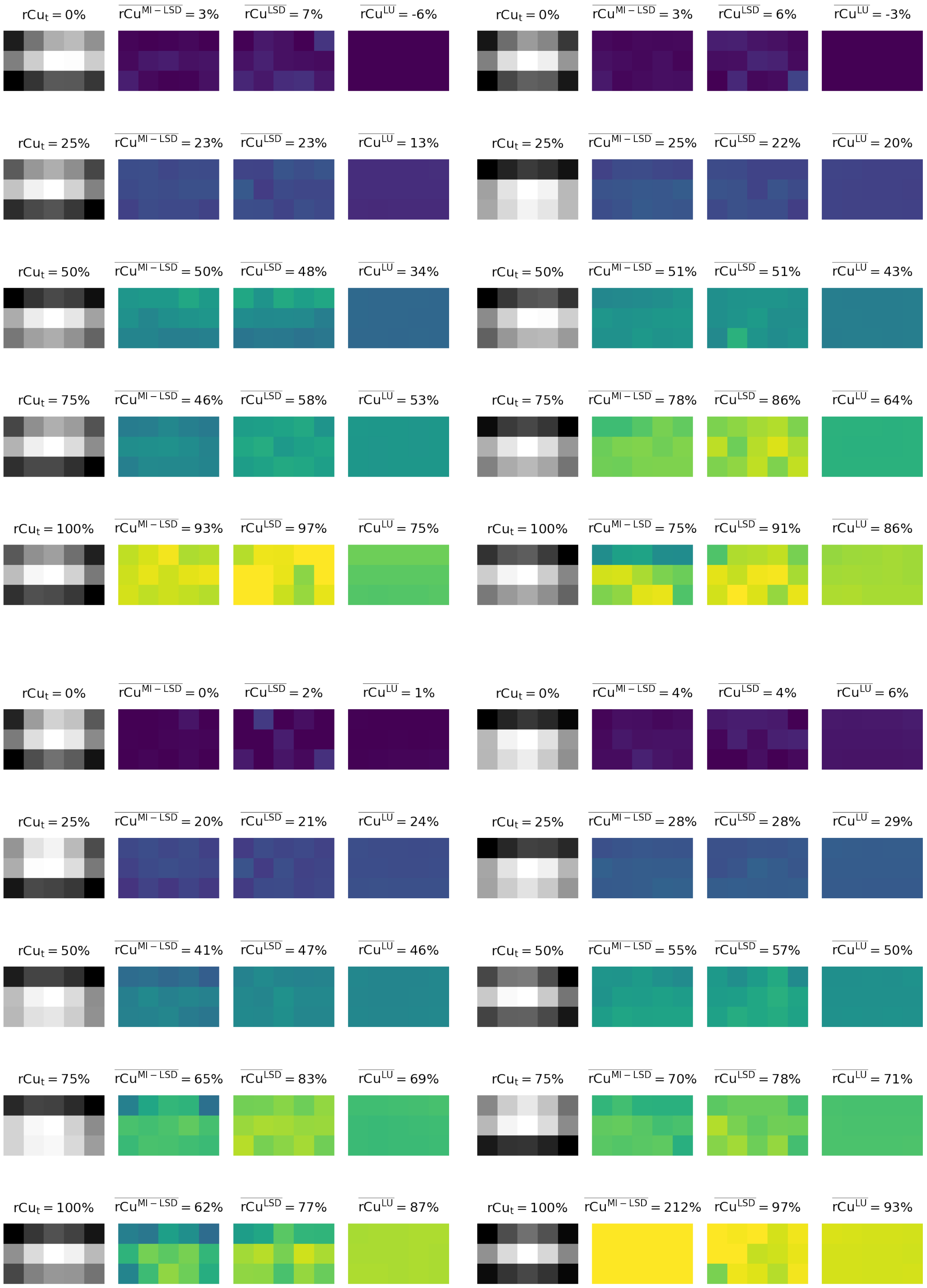

Figure 81: NN (with dropout) estimates on validation phantom with svf= 1.0%, background rCu=[100, 75, 50, 25]% – mean rCu estimates for tubes. Showing PA mean signal and MI-LSD, LSD and LU estimates.

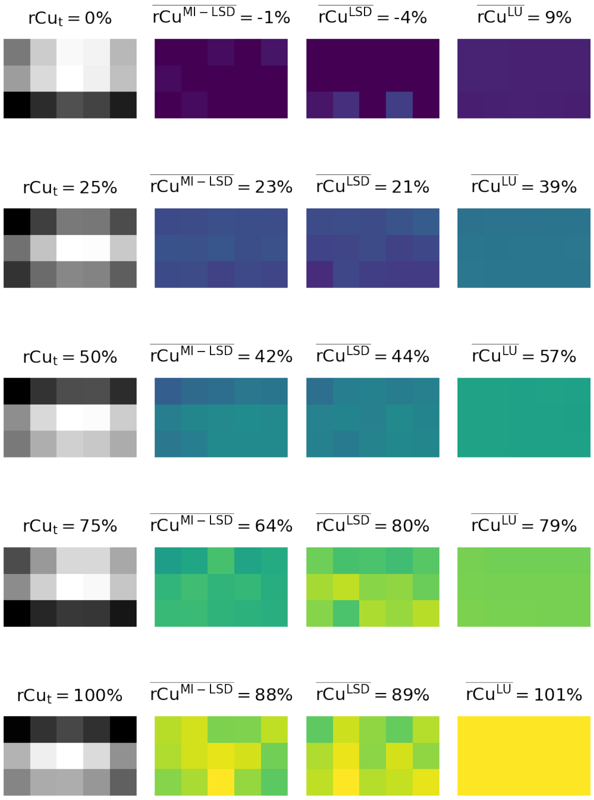

Figure 82: NN (with dropout) estimates on validation phantom with svf= 1.0%, background rCu=0% – mean rCu estimates for tubes. Showing PA mean signal and MI-LSD, LSD and LU estimates.
